# A Systematic Review and Meta‐Analysis of Oncologic Liver Resections in Low‐ and Middle‐Income Countries: Opportunities to Improve Evidence and Outcomes

**DOI:** 10.1002/jso.27928

**Published:** 2024-11-21

**Authors:** Adel H. Khan, Omar Mahmud, Asad Saulat Fatimi, Shaheer Ahmed, Alyssa A. Wiener, Madhuri V. Nishtala, Christopher C. Stahl, Leslie Christensen, Muhammad Rizwan Khan, Patrick B. Schwartz, Syed Nabeel Zafar

**Affiliations:** ^1^ Khyber Medical College Peshawar Pakistan; ^2^ Medical College, Aga Khan University Karachi Pakistan; ^3^ Islamabad Medical and Dental College Islamabad Pakistan; ^4^ Department of Surgery University of Wisconsin School of Medicine and Public Health Madison Wisconsin USA; ^5^ Ebling Library University of Wisconsin Madison Wisconsin USA; ^6^ Department of Surgery Aga Khan University Hospital Karachi Pakistan

**Keywords:** liver neoplasms, liver resection, LMICs, perioperative care, postoperative outcomes

## Abstract

**Background:**

Patients in low‐ and middle‐income countries (LMICs) are disproportionately affected by liver cancers but there is a lack of understanding of their postoperative outcomes. This study aimed to review the current status of research in LMICs regarding outcomes after oncologic hepatectomy and synthesize the data reported in the literature.

**Methods:**

The PubMed, Scopus, Embase, Web of Science, and World Health Organization (WHO) Global Index Medicus databases were searched from database inception to May 26th, 2022. Studies that reported outcomes after oncologic hepatectomy in LMIC settings were eligible for inclusion. Two independent reviewers performed record screening and data extraction. Risk of bias assessment was performed using the National Institutes of Health Study Quality Assessment tools. Pooled results with 95% confidence intervals (95% CIs) were calculated using a random effects model.

**Results:**

One hundred and thirty‐five studies and 16 985 patients were included. Most studies were of a “fair” quality. Two studies described pediatric patients. Only one study was from a low‐income country and most African regions were not represented. The rates of major and minor complications were 11% and 27%, respectively, while 30‐ and 90‐day mortality rates were 2% and 3% each. Postoperative liver failure (8%), surgical site infections (6%), and bile leaks (6%) were common complications.

**Conclusions:**

This review indicates a dearth of data from LMICs on outcomes after hepatectomy, particularly from African regions and low‐income countries.

## 1 Introduction

Liver cancer is the fourth most common cause of cancer‐related deaths and the sixth most prevalent malignancy globally [1]. Primary hepatic tumors include hepatocellular carcinoma, hepatoblastoma, and neoplasms of the biliary tree, such as intrahepatic cholangiocarcinoma. More commonly, the liver is a site of metastatic disease from tumors of other organs, such as the colon, breast, and pancreas [2, 3]. Hepatic tumors collectively account for a significant and growing burden of cancer‐related morbidity and mortality.

The management of liver tumors is typically multimodal, with surgery for local control and systemic therapy to eradicate microscopic or metastatic disease. Major strides have been made in decreasing morbidity and mortality through minimally invasive approaches and effective perioperative care. However, these advances have primarily occurred in high‐income countries (HICs) [4]. Furthermore, the burden of disease caused by liver cancers disproportionately affects low‐ and middle‐income countries (LMICs), with over 80% of liver cancers occurring in these regions [5, 6]. Factors such as a high prevalence of hepatitis B and C virus, delayed presentation of patients, and shortages of resources and trained personnel present continued obstacles to the delivery of optimal care in these settings [7, 8, 9]. These disparities are compounded by broad and enduring challenges faced primarily by LMICs in providing safe and accessible surgery to cancer patients [10].

While there are some large studies assessing outcomes after surgery for colorectal and cervical cancers in LMICs, there has been no comprehensive analysis of outcomes after the resection of malignancies of the liver in these settings [11, 12]. The goals of our systematic review and meta‐analysis were to review the current literature on postoperative outcomes of patients in LMICs undergoing oncologic liver resections, to synthesize the available outcomes data, and to highlight gaps in the available evidence.

## 2 Materials and Methods

### 2.1 Protocol and Registration

This systematic review was prospectively registered with PROSPERO (CRD42021232817) and performed in accordance with the Preferred Reporting Items for Systematic Reviews and Meta‐Analysis (PRISMA) 2020 guidelines [13]. A complete PRISMA checklist is reported in Supporting Information S1: Section 1.

### 2.2 Search Strategy

The PubMed, Scopus, Embase, Web of Science, and World Health Organization (WHO) Global Index Medicus databases were systematically searched for relevant articles published between January 1st, 2005, and May 26th, 2022. Studies published before this period were not included as their results may no longer be representative of practice and outcomes in the current era. The search strategies were developed by a librarian with extensive experience with systematic reviews (LC) and are reported in Supporting Information S1: Section 2. Additional studies were identified manually and through a snowball strategy where the references of related review articles were screened.

### 2.3 Study Eligibility Criteria

All observational studies and randomized controlled trials (RCTs) published in English were eligible for inclusion. Studies were included if they reported postoperative outcomes of adult or pediatric patients from LMICs who underwent liver resection for any cancer (i.e., either primary or metastatic disease). Country income status was identified as per the World Bank List of Economies (2020) [14]. In cases where the classification of a country changed during the study period, the longest classification duration for each study was selected. LMICs included low‐income countries (LICs), lower‐middle‐income countries (lowerMICs), and upper‐middle‐income countries (upperMICs).

Chinese studies were excluded from our analysis, despite China's status as an upperMIC, as the volume of research from academic centers in China is immense and these institutions are increasingly comparable to those in HICs rather than LMICs; the inclusion of these studies would have dominated the review and skewed the pooled results of the meta‐analysis [11, 15, 16]. Studies that analyzed less than 20 patients were also excluded as these are not considered representative. In addition, studies that included patients with benign disease or non‐hepatic resections were excluded.

### 2.4 Data Extraction and Screening

Screening of records by titles and abstracts was performed independently by two reviewers with disagreements resolved by a third reviewer or by consensus amongst the authors. Subsequently, shortlisted articles underwent full text review in a similar process. Ten of the included articles identified by full text screening were randomly assigned to be extracted by two independent reviewers to assess accuracy and concordance, and no errors were noted. As such, the remaining articles were extracted by one reviewer each. Extracted information included study design, participant characteristics, and postoperative outcomes at different time points (in‐hospital, 30‐day, and 90‐day). Postoperative outcomes included operative duration, margin positivity, return to the OR, unplanned intubation, need for endoscopic or percutaneous reintervention, readmission, morbidity and mortality, and specific postoperative complications, including liver failure, wound infection or dehiscence, bile leak, hemorrhage, myocardial infarction, thrombotic or thromboembolic event, cerebrovascular accident, pneumonia, urinary tract infection, liver abscess, bacteremia, and unspecified infections.

### 2.5 Risk of Bias Assessment

The National Institutes of Health (NIH) Study Quality Assessment tools were used as appropriate for each study design [17]. As per previous reviews, questions were each assigned a score of 1, and overall scores were then computed for each study to correspond with poor, fair, or good evaluations [18, 19]. Case series were scored out of 9 and evaluated as poor, fair, or good using score ranges of 0–3, 4–6, and 7–9. Case‐control studies were scored out of 12 and poor, fair, and good assessments were assigned using ranges of 0–4, 5–8, and 9–12. All other studies were scored out of 14 and the corresponding ranges for overall evaluation were 0–5, 6–10, and 11–14.

### 2.6 Statistical Analysis

Two of the analyzed variables, ‘operative time’ and ‘length of stay’, were generally reported as medians and inter‐quartile ranges or ranges. To maximize the data available for meta‐analysis, validated formulae were used to convert these variables to means and standard deviations that could be pooled [20]. However, while this method enabled pooled analysis of these variables, the results are positively skewed and should be interpreted as over‐estimates.

To avoid pooling patients multiple times in each analysis, different studies reported by the same groups during overlapping time periods were identified on a per‐outcome basis. In each instance, the study with the larger sample size was included in the meta‐analysis. This approach minimized the risk of bias due to overlapping cohorts in our results while maximizing the available data for each pooled outcome.

Pooled results, including rates and means, with 95% confidence intervals (95% CIs) were calculated using a Sidik‐Jonkman random effects model on Stata version 18.0 (Stata Corp., College Station, TX, USA). For the meta‐analysis of rates, the Freeman‐Tukey double‐arcsine transformation was applied to adjust for extremes of data, and results were reported as proportions with 95% confidence intervals (95% CIs). All outcomes included more than 10 studies except the rates of unplanned intubation, percutaneous/endoscopic intervention, readmission, 30‐day morbidity, and 90‐day morbidity. Subgroup analyses were performed based on country, national income status, and tumor type. The statistical significance of subgroup differences was assessed using the chi‐squared test. Statistical heterogeneity was measured using the *τ* ^2^ and *I* ^2^ statistics. Assessment of possible causes of heterogeneity included subgroup analyses based on income status, country, and indication. Values of *I* ^2^ above 50% and 75% were taken to indicate moderate and high heterogeneity respectively. Forest plots were generated to depict pooled results. Although funnel plots were generated, the assessment of publication bias was not deemed relevant to our analysis given that comparative data were not extracted or pooled. All statistical tests were two‐sided, with a *p* value of *p* < 0.05 used as the threshold for statistical significance.

## 3 Results

### 3.1 Literature Search

**Figure 1:**
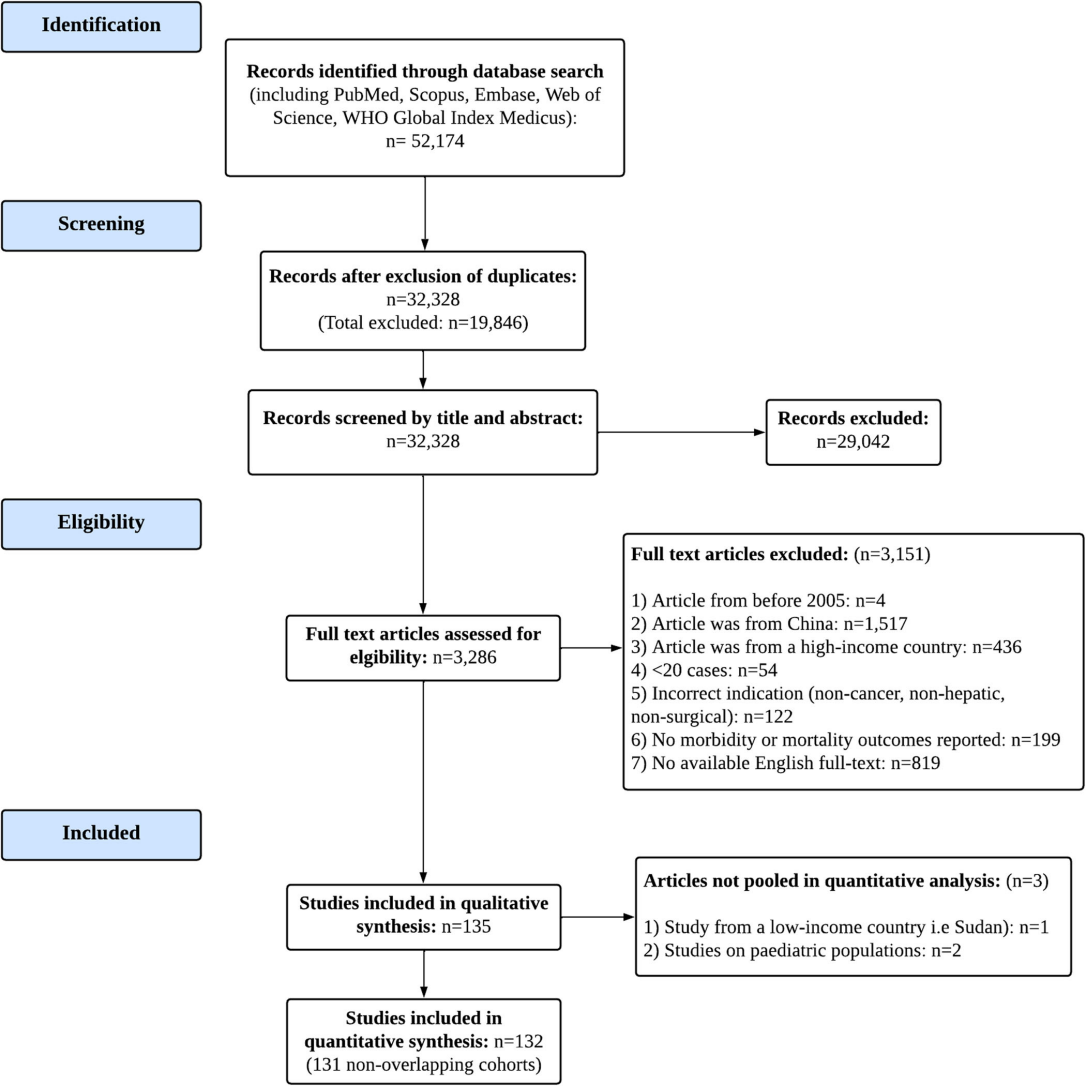
PRISMA flowchart: The flowchart depicts the search identification, screening, and selection process of the systematic review. The number of articles assessed at each stage is reported as are the reasons for filtering and excluding studies that did not satisfy the criteria for inclusion.

Our search strategy yielded 32 328 unique records, of which 135 relevant articles were included in our analysis (Figure 1) [21, 22, 23, 24, 25, 26, 27, 28, 29, 30, 31, 32, 33, 34, 35, 36, 37, 38, 39, 40, 41, 42, 43, 44, 45, 46, 47, 48, 49, 50, 51, 52, 53, 54, 55, 56, 57, 58, 59, 60, 61, 62, 63, 64, 65, 66, 67, 68, 69, 70, 71, 72, 73, 74, 75, 76, 77, 78, 79, 80, 81, 82, 83, 84, 85, 86, 87, 88, 89, 90, 91, 92, 93, 94, 95, 96, 97, 98, 99, 100, 101, 102, 103, 104, 105, 106, 107, 108, 109, 110, 111, 112, 113, 114, 115, 116, 117, 118, 119, 120, 121, 122, 123, 124, 125, 126, 127, 128, 129, 130, 131, 132, 133, 134, 135, 136, 137, 138, 139, 140, 141, 142, 143, 144, 145, 146, 147, 148, 149, 150, 151, 152, 153, 154].

### 3.2 Study Characteristics

**Figure 2:**
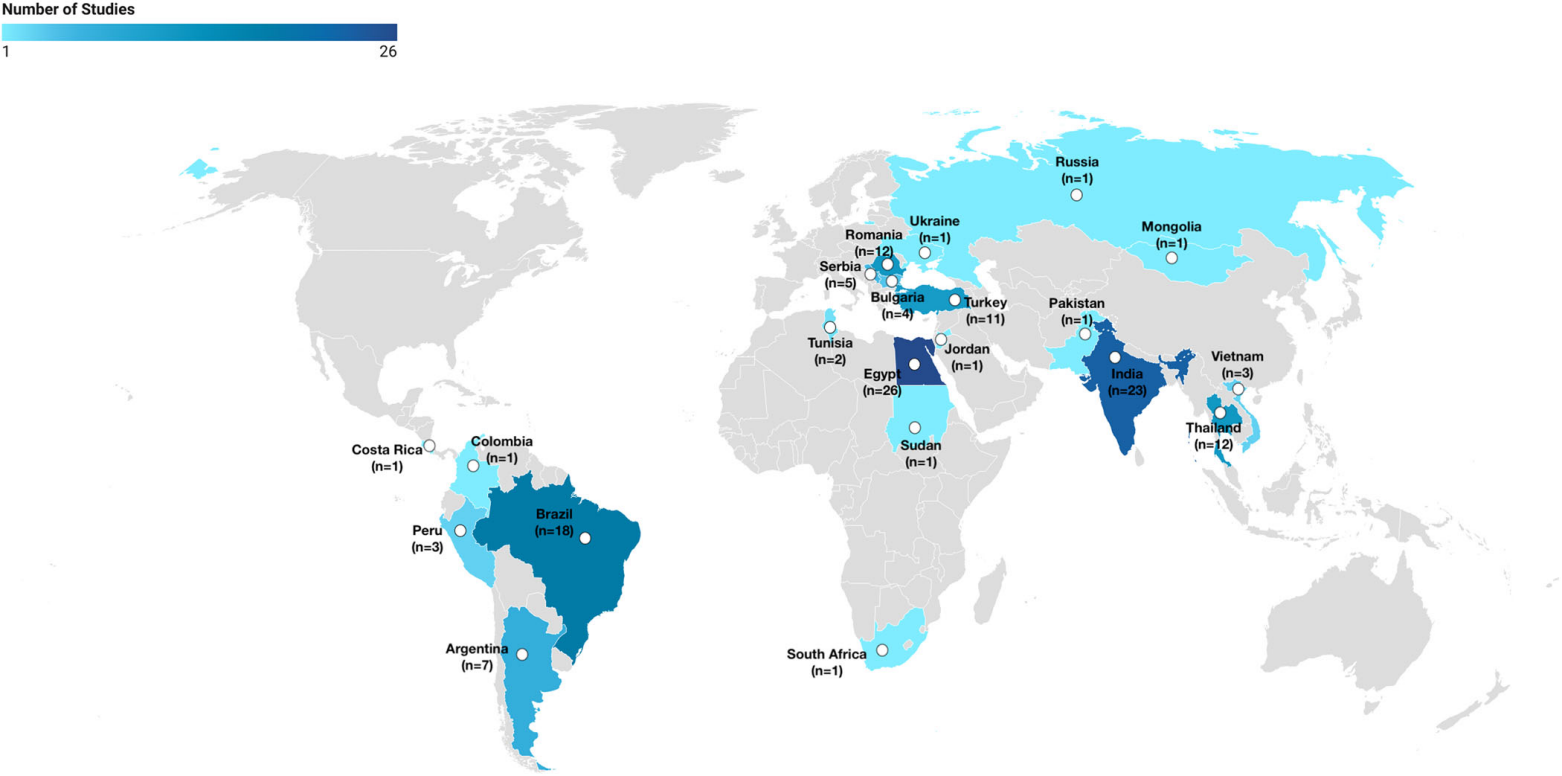
Distribution of studies by country: The number of studies included in the review from each country is depicted in this world map as the shade and intensity of color as indicated. The minimum number of studies per country was 1 and the maximum was 26.

The systematic review identified 24 (17.8%) case series, 100 (74.1%) cohort studies, five (3.7%) case‐control studies, and six (4.4%) quasi‐experimental studies or RCTs. Seventy‐seven (57.0%) studies were from upperMICs while 57 (42.2%) were from lowerMICs. Only one study was from an LIC (Sudan). Twenty‐one countries were represented, with most literature originating from Egypt with 26 (19.3%) studies, followed by India with 23 (17.0%) studies, and Brazil with 18 (13.3%) studies. The global distribution of studies included in this review is displayed in Figure 2. One hundred thirty‐two studies were included in the meta‐analysis. Only descriptive analysis was performed for the two studies which exclusively investigated pediatric patients and the single study from a low‐income country (LIC). These were not pooled in the meta‐analysis as their inclusion would dilute the applicability of the pooled results to the rest of the included studies without allowing for valid or meaningful comparisons.

### 3.3 Participant Characteristics

Eighty‐two studies investigated patients undergoing resection of primary hepatobiliary cancers, the majority of which were hepatocellular carcinoma (HCC) (52/85; 61.2%). Of the studies investigating primary hepatic malignancies, 19 (22.3%) reported the TNM stage and five (5.8%) reported the barcelona clinic liver cancer stage. Overall, 27 (20.0%) studies reported American Society of Anesthesiology (ASA) status, 39 (28.9%) reported Childs‐Pugh class, 11 (8.2%) reported Model for End‐Stage Liver Disease (MELD) scores, and five (3.7%) reported Eastern Cooperative Oncology Group (ECOG) performance status. Positivity for hepatitis B virus (HBV) was reported by 42 (31.1%) studies and Hepatitis C virus (HCV) was reported by 45 (33.3%) studies.

The use of neoadjuvant chemotherapy was reported by 32 (23.7%) studies while that of adjuvant chemotherapy was reported by 28 (20.7%) studies. Fifty‐nine (43.7%) studies explicitly stated the number of open procedures performed while 27 (20.0%) reported laparoscopic surgeries. Only one study reported the performance of robotic surgery. Seventy‐eight (57.8%) and 77 (57.0%) studies provided information on the number of major and minor hepatic resections performed, respectively.

Study and participant characteristics are summarized in Table 1, with additional details in Supporting Information S1: Section 3 **.** Additional results, including the list of studies pooled in each outcome, narrative information about studies in LICs and of pediatric patients, and exploratory subgroup analyses, are reported in Supporting Information S1: Section 4.

**Table 1:** Summary of study and participant characteristics.

Characteristic	Number of studies (%) (*n* = 135)	Total number of patients^a^
Study country—income status		
Upper middle income	77 (57.04%)	9727
Lower middle income	57 (42.22%)	7214
Lower income	1 (0.74%)	44
Surgical indication—primary hepatobiliary cancers (*n* = 85)		
Cholangiocarcinoma	15 (17.65%)	1635
Gallbladder adenocarcinoma	15 (17.65%)	1778
Hepatocellular carcinoma	52 (61.12%)	6255
Pediatric hepatoblastoma	2 (2.35%)	253
Mixed	1 (1.18%)	76
Surgical indication—non‐primary hepatobiliary cancers (*n* = 50)		
Breast cancer	2 (4.00%)	85
Colorectal cancer	34 (68.00%)	5448
Ovarian cancer	2 (4.00%)	70
Neuroendocrine tumors	1 (2.00%)	22
Other^b^	10 (20.00%)	1205
Unspecified	1 (2.00%)	158
Study designs		
Case series	24 (17.78%)	1644
Case‐control	5 (3.70%)	592
Prospective and retrospective cohort	3 (2.22%)	369
Prospective cohort	3 (2.22%)	623
Retrospective cohort	94 (69.63%)	13 424
Quasi‐experimental	1 (0.74%)	40
Randomized controlled trial	5 (3.70%)	293
Preoperative characteristics		
Biological sex	116 (85.93%)	8540^c^
TNM cancer stage^d^	19 (22.35%)	2196
BCLC stage^d^	5 (5.88%)	889
ASA status	27 (20.0%)	3775
Child–Pugh class	39 (28.89%)	4716
MELD score	11 (8.15%)	1040
Hepatitis B infection status	42 (31.10%)	5914
Hepatitis C infection status	45 (33.33%)	5828
ECOG performance status	5 (3.70%)	336
Treatment characteristics		
Neoadjuvant chemotherapy	32 (23.70%)	3849
Adjuvant chemotherapy	28 (20.74%)	3199
Open surgery	59 (43.70%)	6222^e^
Laparoscopic surgery	27 (20.0%)	1460^e^
Robotic surgery	1 (0.74%)	8^e^
Major resection	78 (57.78%)	9626
Minor resection	77 (57.04%)	10 289
Multivisceral resection	23 (17.04%)	3992
Intra/postoperative ablation	18 (13.33%)	2885

*Note:* Number of studies refers to the number of studies that reported the given characteristic/variable in the preoperative and treatment characteristics sections.

Abbreviations: ASA, American Society of Anesthesiologists; BCLC, Barcelona Clinic Liver Cancer; ECOG, Eastern Cooperative Oncology Group; MELD, Model for End‐Stage Liver Disease.

a For whom data were reported (both positive and negative status).

^b^ Indications included a mix of metastatic liver cancer with unspecified primary, colorectal carcinoma, gastric carcinoma, ovarian carcinoma, breast carcinoma, insulinoma, pancreatic ductal adenocarcinoma, esophageal carcinoma, leiomyosarcoma, hemangioendothelioma, angiomyolipoma, retroperitoneal liposarcoma, unspecified neuroendocrine tumors, and gastrointestinal stromal tumor.

^c^ Number of males.

d From studies that investigated patients undergoing resection of primary hepatobiliary cancers (*n* = 82).

e Number of patients who underwent specific kind of procedure.

### 3.4 Meta‐Analysis Results

The pooled in‐hospital morbidity rate was 27% [95% CI: 14%–42%]. The pooled 30‐day morbidity rate was 28% [95% CI: 16%–40%] while 90‐day morbidity was 34% [95% CI: 13%–58%]. The rate of minor complications was found to be 27% [95% CI: 19%–37%], while that of major complications was 11% [95% CI: 8%–14%]. The pooled in‐hospital mortality rate was 5% [95% CI: 2%–9%]. 30‐day mortality was 2% [95% CI: 1%–3%] while 90‐day mortality 3% [95% CI: 1%–5%].

Amongst rates of specific postoperative complications, postoperative liver failure had the highest number of pooled cohorts (64) and patients (8345) and occurred at an overall rate of 8% [95% CI: 6%–12%], making it the most common complication. Other common complications included wound infections (6% [95% CI: 4%–8%]) and bile leaks (6% [95% CI: 4%–8%]).

Of all pooled results, margin status had the highest number of pooled cohorts (66) and patients (8739). The rate of positive surgical margins was 9% [95% CI: 6%–12%]. This result did not significantly differ across upperMIC and lowerMIC subgroups. However, testing for subgroup differences showed significant variability across individual countries (*p *< 0.01) and tumor types (*p *< 0.01).

**Figure 3:**
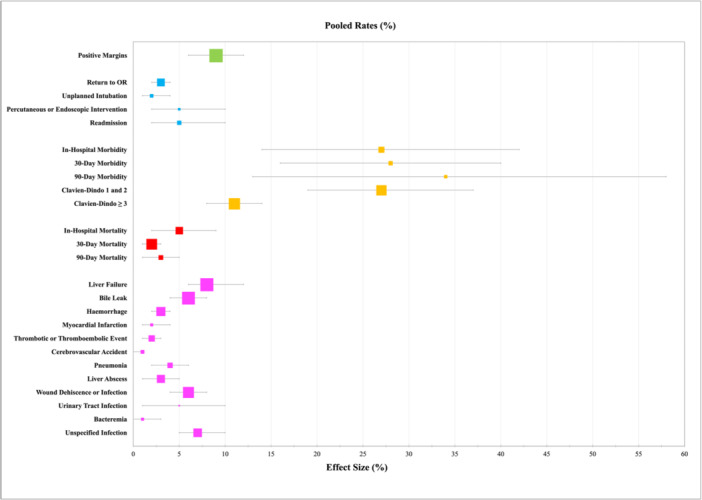
Summary of pooled rates of postoperative complications: The pooled point estimate for each rate (%) is depicted by the distance of the squares along the horizontal axis, while the size of each square depicts the number of patients pooled for the outcome. The error bars depict the 95% confidence interval.

These results are summarized in Table 2, and a forest plot of pooled rates is depicted in Figure 3. A complete breakdown of all analysis results, forest plots, and funnel plots for all outcomes included in the meta‐analysis are present in Supporting Information S1: Section 4.

**Table 2:** Summary of pooled outcomes.

Variables	Number of cohorts	Total patients	Pooled outcome (95% CI)	*I* ^2^	*τ* ^2^
Procedure time					
Operative duration (minutes)	45	5472	200.34 (173.02, 227.65)	81.07	4448.87
Margin status					
Positive margins	66	8739	0.09 (0.06, 0.12)	95.54	0.16
Postoperative course					
Return to OR	22	3002	0.03 (0.02, 0.04)	61.41	0.01
Unplanned intubation	2	632	0.02 (0.01, 0.04)	31.29	0.00
Percutaneous or endoscopic intervention	8	331	0.05 (0.02, 0.10)	43.98	0.02
Readmission	7	917	0.05 (0.02, 0.10)	73.56	0.03
Length of stay (days)	45	6950	8.33 (6.81, 9.85)	78.39	11.07
Morbidity					
In‐hospital morbidity	13	1802	0.27 (0.14, 0.42)	97.53	0.31
30‐day morbidity	4	921	0.28 (0.16, 0.40)	89.10	0.06
90‐day morbidity	5	586	0.34 (0.13, 0.58)	96.61	0.30
Clavien‐Dindo 1 and 2	34	5240	0.27 (0.19, 0.37)	97.96	0.32
≥Clavien‐Dindo 3	44	6279	0.11 (0.08, 0.14)	92.30	0.09
Mortality					
In‐hospital mortality	20	2752	0.05 (0.02, 0.09)	94.31	0.13
30‐day mortality	39	5633	0.02 (0.01, 0.03)	77.21	0.02
90‐day mortality	13	1097	0.03 (0.01, 0.05)	59.20	0.02
Specific complications					
Liver failure	64	8345	0.08 (0.06, 0.12)	95.46	0.16
Bile leak	64	8004	0.06 (0.04, 0.08)	89.67	0.07
Hemorrhage	40	4035	0.03 (0.02, 0.04)	62.29	0.02
Myocardial infarction	5	441	0.02 (0.01, 0.04)	15.54	0.00
Thrombotic or thromboembolic event	16	1972	0.02 (0.01, 0.03)	40.60	0.01
Cerebrovascular accident	3	709	0.01 (0.00, 0.01)	10.23	0.00
Pneumonia	17	1414	0.04 (0.02, 0.06)	66.51	0.03
Liver abscess	19	3210	0.03 (0.01, 0.05)	79.99	0.02
Wound dehiscence or infection	48	5922	0.06 (0.04, 0.08)	86.09	0.05
Urinary tract infection	4	119	0.05 (0.01, 0.10)	8.71	0.00
Bacteremia	6	448	0.01 (0.00, 0.03)	16.01	0.00
Unspecified infection	37	3629	0.07 (0.05, 0.10)	82.76	0.05

*Note:* Red indicates a significant *p* value (< 0.05).

Abbreviations: CI, confidence interval; OR, operating room.

### 3.5 Risk of Bias Assessment and Publication Bias

Ninety‐nine (73.3%) of the included studies were rated to be “fair” quality while 34 (25.2%) of studies were rated to be “good” quality. Only two studies (1.5%), both of which were retrospective cohorts, were rated to be “poor" quality. There were no significant differences between study ratings between upperMICs and lowerMICs (*p *= 0.187). Domain‐wise assessments for all included studies are present in Supporting Information S1: Section 5.

## 4 Discussion

This study aimed to systematically review patient outcomes after undergoing liver resection for malignant tumors in LMICs. It represents the first review to characterize the geographic distribution of relevant studies and evaluate rates of postoperative outcomes in LMICs. While these pooled results provide quantitative summaries of the available evidence, an important finding of this review is implied by what is absent. We found that the reporting of meaningful study and patient data remains poor and omits the crucial context of factors, such as patient comorbidities or the extent of resection, that influence patient outcomes. Furthermore, there was a conspicuous absence of data from LICs. Our review highlights the lack of high‐quality surgical outcomes research in many parts of the world. Pooled estimates are provided by country resource level, and these can be used as a benchmark to improve the quality of liver cancer surgery in LMICs.

Our meta‐analysis included 27 pooled outcomes that described operative duration, surgical margins, postoperative course, morbidity, and mortality. Across these, we found significant variation in outcomes. The three most common postoperative complications were liver failure (8%), wound infections (6%) and bile leaks (6%). Infectious complications, length of stay, and in‐hospital mortality were high in the pooled analyses and are areas worth targeting for improvement, as are the rates of minor (Clavien‐Dindo Grade 1 or 2, 27%) and major (Clavien‐Dindo Grade > 2, 11%) complications. It is worth noting that several studies did report complications rates similar to those reported by some HICs indicating that postoperative outcomes in LMIC settings can be optimized to reach those of an HIC. Although some results appeared to be better than those generally found in the literature, they must be interpreted with caution. For example, the overall readmission rate was 5% in our analysis, whereas large studies from HICs can report rates as high as 15% [155, 156]. Readmission rates are difficult to capture in LMIC settings and are subject to both selection bias and immortal time bias depending on how they are reported [157]. Moreover, it is important to bear in mind that the studies represented here may be more representative of urban, high‐performing, high‐volume academic centers within LMICs that have the resources and skillsets to perform and publish outcomes.

A consistent finding in the systematic review was the lack of a standardized reporting schema for patients undergoing hepatectomy. Factors that modify the risks of morbidity and mortality, such as ASA scores, comorbidities, and ECOG scores, are essential to contextualize the findings of a study. Moreover, aspects of treatment including open versus minimally‐invasive surgery, operative techniques, receipt of perioperative chemotherapy, and the extent of resection, can all affect outcomes [158, 159, 160, 161, 162]. Of particular importance is the measurement of the future liver remnant (FLR), which can indicate the extent of hepatic function that a patient is expected to retain after surgery [163]. This can guide both management and prognostication. For example, interventions such as trans‐arterial chemoembolization or the use of yttrium‐90 microsphere radioembolization are often utilized in patients who are not initially surgical candidates to increase FLR [163, 164, 165, 166]. In general, we found that few studies reported data pertaining to these relevant variables which precluded an analysis of risk‐stratified outcome rates. This also limited our ability to analyze the current utilization and availability of minimally invasive modalities or adjunct interventions in LMICs. It is crucial for future outcome studies to measure and report these variables to enhance the accuracy of outcome reporting and characterize the technical capacity of centers in LMIC settings [167].

We found that 6% of patients developed SSIs. SSIs are the most common healthcare‐associated infection in LMICs and a major cause of preventable morbidity and mortality that affects all patients, including those deemed “low‐risk” [168, 169, 170]. Previous studies have shown that improvements in the rates of postoperative infections in such settings can be achieved through the implementation of feasible preventive measures, such as the 2018 WHO guidelines for the prevention of SSIs [168, 171]. Providers in low‐resource settings may find our results to be helpful context when evaluating the scope for and success with the adoption of such evidence‐based practices.

Despite evaluating over 32 000 unique abstracts, only a single study in the current systematic review, from Sudan, was from an LIC. Thus, we were not able to incorporate LICs into our pooled analysis. Moreover, most African countries were not represented. A previous review on postoperative outcomes after the resection of cervical cancer in LMICs found a similar paucity of evidence from LICs [11]. These results indicate a clear lack of data on surgical quality from low‐income settings and efforts to develop research capacity are crucial. Studies like the African Surgical Outcomes Study demonstrate that such research is possible even in very low‐resource settings through concerted efforts and collaborations [172]. Academic partnerships between institutions in HICs and LICs, such as those between the American College of Surgeons and various partners in Africa, can play a crucial role in assisting developing countries in acquiring the capacity needed to investigate, report, and improve surgical quality and patient outcomes [173]. Future research may refer to the findings of our review to identify and target gaps in the literature.

There are several limitations to this study. Characterizing routine morbidity and mortality rates through study‐level data can be challenging due to publication bias and differences in definitions across studies. The clarity of certain outcomes, like 30‐ and 90‐day morbidity rates, was constrained by the limited volume of available data, which affected our understanding of the postoperative complication timeline. However, mortality outcomes were not affected by this limitation. We were able to pool a large number of studies, allowing us to analyze the severity and nature of morbidity—including minor and major complications, as well as specific complication rates—to adequately characterize postoperative complications. Subpar outcomes may be less likely to be published as opposed to series with unusually low rates of morbidity and mortality [174]. Moreover, the representation of different countries in the analysis was skewed, with minimal data available from sub‐Saharan Africa and nearly half of included studies originated from India, Egypt, and Brazil. We excluded studies with less than 20 patients to enable a more representative analysis. However, many low‐income or low‐middle‐income countries may have published smaller studies. These case series or case reports are not included in our analysis but given the paucity of data, these may provide additional valuable insight to the experience with hepatectomies in LMICs and could be considered in future reviews. We also only included patients with malignancies in our study. The inclusion of benign causes of hepatectomies in our review would increase the total number of eligible studies. However, this would also further increase the heterogeneity in the study. In our review, we aimed to highlight the outcomes and research gaps specific to cancer surgery and therefore decided to focus on studies that include only patients with cancers. Most outcomes also showed moderate to high heterogeneity. This type of heterogeneity is not uncommon in prevalence meta‐analyses and was accounted for by the use of the Sidik‐Jonkman random effects model to provide conservative estimates of precision in the results [175, 176, 177, 178]. Given that our results are pooled rates and not comparisons or hypothesis tests, the heterogeneity seen is not of major consequence to our findings and is impounded in the confidence intervals reported, which provide an index of the precision of the estimated rates. Finally, as mentioned previously, we were not able to stratify the results of the analysis by conventional patient (e.g., ASA score, Childs Pugh score) and operative (i.e., extent of hepatectomy and use of minimally invasive techniques) factors due to a lack of reporting.

## 5 Conclusion

To our knowledge, this is the first systematic review and meta‐analysis to examine surgical outcomes after hepatectomies in LMICs and we provide important context for global surgical oncology research. Our review highlights gaps in the literature from LMICs and demonstrates the need to enhance outcomes research in these settings, particularly in LICs and African regions. Our meta‐analysis pools a large volume of data on outcomes in MICs and identifies several postoperative outcomes that may be targeted for improvement. However, the absence of risk‐adjusted data and the scarce reporting of important confounders limits the certainty of these results. As the burden of hepatic malignancies continues to rise in LMICs, efforts to improve patient outcomes will benefit from increased scope, quality, and efficiency of evidence synthesis and reporting to provide actionable data in these settings.

## Conflicts of Interest

The authors declare no conflicts of interest.

## Synopsis

This systematic review and meta‐analysis examined nearly 33 000 published records to identify studies on outcomes after oncologic hepatectomy in LMICs. We identify a dearth of literature from low‐income countries and provide pooled rates of various indices of surgical quality, morbidity, and mortality.

## Supporting information

Supporting information.

## Data Availability

All data have been derived from previously published studies.
