# Supplementary material for: A Systematic Review and Meta‐Analysis of Oncologic Liver Resections in Low‐ and Middle‐Income Countries: Opportunities to Improve Evidence and Outcomes
Source: J Surg Oncol. 2024 Nov 21;131(5):865–78. doi: 10.1002/jso.27928 (PMC12120391; doi:10.1002/jso.27928)
Supplement: Supplementary file 1 — Supporting information. [file JSO-131-865-s001.docx]

**A Systematic Review and Meta-Analysis of Oncologic Liver Resections in Low- and Middle-Income Countries: Opportunities to Improve Evidence and Outcomes**

**Supplementary Material**

*Supplementary Section 1:* PRISMA Checklist (2020)

*Supplementary Section 2:* Detailed Search Strings

*Supplementary Section 3:* Study and Participant Characteristics

*Supplementary Section 4:* Extended Results, Forest Plots, and Funnel Plots

*Supplementary Section 5:* Risk of Bias Assessment

**Section 1: PRISMA Checklist (2020)**

| **Section and Topic** | **Item #** | **Checklist item** | **Reported on Page #** |
| --- | --- | --- | --- |
| **TITLE** | | |  |
| Title | 1 | Identify the report as a systematic review. | 1 |
| **ABSTRACT** | | |  |
| Abstract | 2 | See the PRISMA 2020 for Abstracts checklist. | 2 |
| **INTRODUCTION** | | |  |
| Rationale | 3 | Describe the rationale for the review in the context of existing knowledge. | 3 |
| Objectives | 4 | Provide an explicit statement of the objective(s) or question(s) the review addresses. | 3 |
| **METHODS** | | |  |
| Eligibility criteria | 5 | Specify the inclusion and exclusion criteria for the review and how studies were grouped for the syntheses. | 4 |
| Information sources | 6 | Specify all databases, registers, websites, organisations, reference lists and other sources searched or consulted to identify studies. Specify the date when each source was last searched or consulted. | 4, S5 |
| Search strategy | 7 | Present the full search strategies for all databases, registers and websites, including any filters and limits used. | 4, S5-14 |
| Selection process | 8 | Specify the methods used to decide whether a study met the inclusion criteria of the review, including how many reviewers screened each record and each report retrieved, whether they worked independently, and if applicable, details of automation tools used in the process. | 4-5 |
| Data collection process | 9 | Specify the methods used to collect data from reports, including how many reviewers collected data from each report, whether they worked independently, any processes for obtaining or confirming data from study investigators, and if applicable, details of automation tools used in the process. | 5 |
| Data items | 10a | List and define all outcomes for which data were sought. Specify whether all results that were compatible with each outcome domain in each study were sought (e.g. for all measures, time points, analyses), and if not, the methods used to decide which results to collect. | 5-6 |
|  | 10b | List and define all other variables for which data were sought (e.g. participant and intervention characteristics, funding sources). Describe any assumptions made about any missing or unclear information. | 5-6 |
| Study risk of bias assessment | 11 | Specify the methods used to assess risk of bias in the included studies, including details of the tool(s) used, how many reviewers assessed each study and whether they worked independently, and if applicable, details of automation tools used in the process. | 5 |
| Effect measures | 12 | Specify for each outcome the effect measure(s) (e.g. risk ratio, mean difference) used in the synthesis or presentation of results. | 6 |
| Synthesis methods | 13a | Describe the processes used to decide which studies were eligible for each synthesis (e.g. tabulating the study intervention characteristics and comparing against the planned groups for each synthesis (item #5)). | 6 |
|  | 13b | Describe any methods required to prepare the data for presentation or synthesis, such as handling of missing summary statistics, or data conversions. | 6 |
|  | 13c | Describe any methods used to tabulate or visually display results of individual studies and syntheses. | 6 |
|  | 13d | Describe any methods used to synthesize results and provide a rationale for the choice(s). If meta-analysis was performed, describe the model(s), method(s) to identify the presence and extent of statistical heterogeneity, and software package(s) used. | 6 |
|  | 13e | Describe any methods used to explore possible causes of heterogeneity among study results (e.g. subgroup analysis, meta-regression). | 6 |
|  | 13f | Describe any sensitivity analyses conducted to assess robustness of the synthesized results. | 6 |
| Reporting bias assessment | 14 | Describe any methods used to assess risk of bias due to missing results in a synthesis (arising from reporting biases). | 5 |
| Certainty assessment | 15 | Describe any methods used to assess certainty (or confidence) in the body of evidence for an outcome. | 6 |
| **RESULTS** | | |  |
| Study selection | 16a | Describe the results of the search and selection process, from the number of records identified in the search to the number of studies included in the review, ideally using a flow diagram. | 6-7, 31 |
|  | 16b | Cite studies that might appear to meet the inclusion criteria, but which were excluded, and explain why they were excluded. | 31 |
| Study characteristics | 17 | Cite each included study and present its characteristics. | 7, 27-28, 32, S15-29 |
| Risk of bias in studies | 18 | Present assessments of risk of bias for each included study. | 8-9, S70-81 |
| Results of individual studies | 19 | For all outcomes, present, for each study: (a) summary statistics for each group (where appropriate) and (b) an effect estimate and its precision (e.g. confidence/credible interval), ideally using structured tables or plots. | 27-28 |
| Results of syntheses | 20a | For each synthesis, briefly summarise the characteristics and risk of bias among contributing studies. | 7-9, 27-28, 32, S15-29, S70-81 |
|  | 20b | Present results of all statistical syntheses conducted. If meta-analysis was done, present for each the summary estimate and its precision (e.g. confidence/credible interval) and measures of statistical heterogeneity. If comparing groups, describe the direction of the effect. | 8-9, 28-29, 33, S30-69 |
|  | 20c | Present results of all investigations of possible causes of heterogeneity among study results. | S38-60 |
|  | 20d | Present results of all sensitivity analyses conducted to assess the robustness of the synthesized results. | 28-29, 33, S38-69 |
| Reporting biases | 21 | Present assessments of risk of bias due to missing results (arising from reporting biases) for each synthesis assessed. | 8-9 |
| Certainty of evidence | 22 | Present assessments of certainty (or confidence) in the body of evidence for each outcome assessed. | 8, 28-29, 33, S30-37 |
| **DISCUSSION** | | |  |
| Discussion | 23a | Provide a general interpretation of the results in the context of other evidence. | 10-11 |
|  | 23b | Discuss any limitations of the evidence included in the review. | 12 |
|  | 23c | Discuss any limitations of the review processes used. | 12 |
|  | 23d | Discuss implications of the results for practice, policy, and future research. | 11-13 |
| **OTHER INFORMATION** | | |  |
| Registration and protocol | 24a | Provide registration information for the review, including register name and registration number, or state that the review was not registered. | 3-4 |
|  | 24b | Indicate where the review protocol can be accessed, or state that a protocol was not prepared. | 3-4 |
|  | 24c | Describe and explain any amendments to information provided at registration or in the protocol. | - |
| Support | 25 | Describe sources of financial or non-financial support for the review, and the role of the funders or sponsors in the review. | 1 |
| Competing interests | 26 | Declare any competing interests of review authors. | 1 |
| Availability of data, code and other materials | 27 | Report which of the following are publicly available and where they can be found: template data collection forms; data extracted from included studies; data used for all analyses; analytic code; any other materials used in the review. | 1, 3-6, 14-26 |

**Section 2: Detailed Search Strings**

| **Original Searches / Databases Searched** | **Date Searched** | **Results** |
| --- | --- | --- |
| PubMed | 3/09/2021 | **16,393** |
| Embase (Scopus) | 3/09/2021 | **14,197** |
| SCI-Expanded, ESCI (Web of Science) | 3/09/2021 | **11,311** |
| Global Index Medicus (WHO) | 3/09/2021 | **3,798** |
| Total | | **45,699** |
| After librarian de-duplication | | **28,462** |
| After Covidence de-duplication | | **28,458** |

| **Search Update / Databases Searched** | **Date Searched** | **Results** |
| --- | --- | --- |
| PubMed | 5/26/2022 | **2,496** |
| Embase (Scopus) | 5/26/2022 | **2,128** |
| SCI-Expanded, ESCI (Web of Science) | 5/26/2022 | **1,607** |
| Global Index Medicus (WHO) | 5/26/2022 | **244** |
| Total | |  |
| After librarian de-duplication | | **3,873** |
| After Covidence de-duplication | | **3,870** |

**Detailed Search Methods**

The review team collaborated with a research librarian (LC) to develop and execute a comprehensive search of the literature. This search combined controlled vocabulary and keyword terms related to liver cancer surgery outcomes in low- and middle-income countries (LMIC). The search was developed in PubMed and translated for the following databases: Embase (Scopus), Global Index Medicus (World Health Organization), and Science Citation Index-Expanded and Emerging Sources Citation Index through Web of Science (Clarivate). A LMIC filter developed by the Cochrane Effective Practice and Organisation of Care (EPOC) group based on the 2019 World Bank list of economies was adapted to include current and retrospective low- and middle-income country names, general LMIC terms and demonyms. Additional filters were applied to remove animal studies, studies with fewer than 50 subjects, and the following non-primary research publication types from the results: editorials, commentaries, letters, systematic reviews, and meta-analyses. All databases were searched on March 9, 2021, with a date limit of January 1, 2005 - present to capture studies with current morbidity and mortality results. An additional search was run on May 26, 2022 from the original search date to present to identify new studies for inclusion. No language filter was applied to the results. Results were downloaded to a citation management software (EndNote) and underwent manual deduplication by the research librarian. Unique records were uploaded to a screening platform (Covidence) for independent review by team members using a pre-determined inclusion/exclusion criteria.

**Original Searches**

**PubMed: 16,393 results**

("Liver Neoplasms"[mesh] OR "Hepatoblastoma"[mesh] OR "Biliary Tract Neoplasms"[mesh] OR "Cholangiocarcinoma"[mesh] OR (hepatoblastoma*[tiab] OR hepatocell*[tiab] OR HCC[tiab] OR cholangiocarcinoma*[tiab] OR hepatocholangiocarcinoma*[tiab] OR CHCC[tiab] OR (("Liver"[mesh] OR "Biliary Tract"[mesh] OR (liver*[tiab] OR hepatic*[tiab] OR biliary[tiab] OR hepatobiliary[tiab] OR hepatopancrea*[tiab] OR pancreatobiliary[tiab] OR bile-canalicul*[tiab] OR gallbladder*[tiab] OR cholangiocellular*[tiab] OR klatskin*[tiab] OR bile-duct*[tiab])) AND (neoplas*[tiab] OR cancer*[tiab] OR adenoma*[tiab] OR carcinoma*[tiab] OR adenocarcinoma*[tiab] OR malignan*[tiab] OR tumor*[tiab] OR tumour*[tiab] OR metasta*[tiab] OR lesion*[tiab] OR mass*[tiab])))) AND ("Hepatectomy"[mesh] OR "Metastasectomy"[mesh] OR "Cholecystectomy"[mesh] OR (hepatectom*[tiab] OR posthepatectom*[tiab] OR post-hepatectom*[tiab] OR hepathectom*[tiab] OR hepatopancreatoduodenectom*[tiab] OR metastasectom*[tiab] OR postmetastasectom*[tiab] OR post-metastasectom*[tiab] OR cholecystectom*[tiab] OR lobectom*[tiab] OR postlobectom*[tiab] OR post-lobectom*[tiab] OR surger*[tiab] OR surgery[sh] OR surgical*[tiab] OR resection*[tiab] OR debulk*[tiab])) AND (“afghanistan”[mesh] OR “albania”[mesh] OR “algeria”[mesh] OR “american samoa”[mesh] OR “angola”[mesh] OR “argentina”[mesh] OR “armenia”[mesh] OR “azerbaijan”[mesh] OR “bangladesh”[mesh] OR “republic of belarus”[mesh] OR “belize”[mesh] OR “benin”[mesh] OR “bhutan”[mesh] OR “bolivia”[mesh] OR “bosnia and herzegovina”[mesh] OR “botswana”[mesh] OR “brazil”[mesh] OR “bulgaria”[mesh] OR “burkina faso”[mesh] OR “burundi”[mesh] OR “cabo verde”[mesh] OR “cambodia”[mesh] OR “cameroon”[mesh] OR “central african republic”[mesh] OR “chad”[mesh] OR “chile”[mesh] OR “china”[mesh] OR “colombia”[mesh] OR “comoros”[mesh] OR “democratic republic of the congo”[mesh] OR “congo”[mesh] OR “costa rica”[mesh] OR “cote d’ivoire”[mesh] OR “croatia”[mesh] OR “cuba”[mesh] OR “djibouti”[mesh] OR “dominica”[mesh] OR “dominican republic”[mesh] OR “ecuador”[mesh] OR “egypt”[mesh] OR “el salvador”[mesh] OR “equatorial guinea”[mesh] OR “eritrea”[mesh] OR “estonia”[mesh] OR "Eswatini"[Mesh] OR “ethiopia”[mesh] OR “fiji”[mesh] OR “gabon”[mesh] OR “gambia”[mesh] OR “georgia (republic)“[mesh] OR “ghana”[mesh] OR “gibraltar”[mesh] OR “grenada”[mesh] OR “guam”[mesh] OR “guatemala”[mesh] OR “guinea”[mesh] OR “guinea-bissau”[mesh] OR “guyana”[mesh] OR “haiti”[mesh] OR “honduras”[mesh] OR “hungary”[mesh] OR “india”[mesh] OR “indonesia”[mesh] OR “iran”[mesh] OR “iraq”[mesh] OR “jamaica”[mesh] OR “jordan”[mesh] OR “kazakhstan”[mesh] OR “kenya”[mesh] OR “democratic people’s republic of korea”[mesh] OR “kosovo”[mesh] OR “kyrgyzstan”[mesh] OR “laos”[mesh] OR “latvia”[mesh] OR “lebanon”[mesh] OR “lesotho”[mesh] OR “liberia”[mesh] OR “libya”[mesh] OR “lithuania”[mesh] OR “republic of north macedonia”[mesh] OR “madagascar”[mesh] OR “malawi”[mesh] OR “malaysia”[mesh] OR “indian ocean islands”[mesh] OR “mali”[mesh] OR “micronesia”[mesh] OR “palau”[mesh] OR “mauritania”[mesh] OR “mauritius”[mesh] OR “mexico”[mesh] OR “moldova”[mesh] OR “mongolia”[mesh] OR “montenegro”[mesh] OR “morocco”[mesh] OR “mozambique”[mesh] OR “myanmar”[mesh] OR “namibia”[mesh] OR “nepal”[mesh] OR “nicaragua”[mesh] OR “niger”[mesh] OR “nigeria”[mesh] OR “oman”[mesh] OR “pakistan”[mesh] OR “panama”[mesh] OR “papua new guinea”[mesh] OR “paraguay”[mesh] OR “peru”[mesh] OR “philippines”[mesh] OR “poland”[mesh] OR “romania”[mesh] OR “russia”[mesh] OR “rwanda”[mesh] OR “samoa”[mesh] OR “sao tome and principe”[mesh] OR “senegal”[mesh] OR “serbia”[mesh] OR “seychelles”[mesh] OR “sierra leone”[mesh] OR “slovakia”[mesh] OR “slovenia”[mesh] OR “melanesia”[mesh] OR “somalia”[mesh] OR “south africa”[mesh] OR “south sudan”[mesh] OR “sri lanka”[mesh] OR “saint kitts and nevis”[mesh] OR “saint lucia”[mesh] OR “saint vincent and the grenadines”[mesh] OR “sudan”[mesh] OR “suriname”[mesh] OR “syria”[mesh] OR “tajikistan”[mesh] OR “tanzania”[mesh] OR “thailand”[mesh] OR “timor-leste”[mesh] OR “togo”[mesh] OR “tonga”[mesh] OR “trinidad and tobago”[mesh] OR “tunisia”[mesh] OR “turkey”[mesh] OR “turkmenistan”[mesh] OR “uganda”[mesh] OR “ukraine”[mesh] OR “uruguay”[mesh] OR “uzbekistan”[mesh] OR “vanuatu”[mesh] OR “venezuela”[mesh] OR “vietnam”[mesh] OR “middle east”[mesh] OR “yemen”[mesh] OR “yugoslavia”[mesh] OR “zambia”[mesh] OR “zimbabwe”[mesh] OR “africa south of the sahara”[mesh] OR “africa, central”[mesh] OR “africa, northern”[mesh] OR “africa, southern”[mesh] OR “africa, eastern”[mesh] OR “africa, western”[mesh] OR “west indies”[mesh] OR “indian ocean islands”[mesh] OR “caribbean region”[mesh] OR “central america”[mesh] OR “latin america”[mesh] OR “south america”[mesh] OR “asia, central”[mesh] OR “asia, northern”[mesh] OR “asia, southeastern”[mesh] OR “asia, western”[mesh] OR “europe, eastern”[mesh] OR “developing countries”[mesh] OR (afghan*[tiab] OR africa*[tiab] OR albania*[tiab] OR algeria*[tiab] OR angola*[tiab] OR argentin*[tiab] OR armenia*[tiab] OR azerbaijan*[tiab] OR bangladesh*[tiab] OR bangalee*[tiab] OR bajan*[tiab] OR belarus*[tiab] OR byelarus*[tiab] OR belorus*[tiab] OR byelorus*[tiab] OR belize*[tiab] OR benin*[tiab] OR dahomey[tiab] OR bhutan*[tiab] OR bolivia*[tiab] OR bosnia*[tiab] OR herzegovin*[tiab] OR botswana*[tiab] OR batswana*[tiab] OR bechuanaland*[tiab] OR brazil*[tiab] OR brasil*[tiab] OR bulgaria*[tiab] OR burkina*[tiab] OR upper-volta[tiab] OR burundi*[tiab] OR urundi*[tiab] OR cabo-verde*[tiab] OR cape-verde*[tiab] OR cambodia*[tiab] OR kampuchea*[tiab] OR khmer*[tiab] OR cameroon*[tiab] OR cameron*[tiab] OR cameroun*[tiab] OR ubangi*[tiab] OR chad[tiab] OR chadian*[tiab] OR chile[tiab] OR chilean*[tiab] OR china[tiab] OR chinese[tiab] OR colombia*[tiab] OR comoro*[tiab] OR comorian*[tiab] OR mayotte*[tiab] OR congo[tiab] OR congolese[tiab] OR zaire*[tiab] OR costa-rica*[tiab] OR cote-d-ivoir*[tiab] OR ivory-coast*[tiab] OR ivorian*[tiab] OR croatia*[tiab] OR cuba*[tiab] OR djibouti*[tiab] OR dominica*[tiab] OR ecuador*[tiab] OR egypt*[tiab] OR united-arab-republic*[tiab] OR salvador*[tiab] OR equatoguinean*[tiab] OR eritrea*[tiab] OR estonia*[tiab] OR eswatin*[tiab] OR swazi*[tiab] OR swati*[tiab] OR ethiopia*[tiab] OR fiji*[tiab] OR gabon*[tiab] OR gambia*[tiab] OR georgia*[tiab] OR ghana*[tiab] OR gold-coast*[tiab] OR gibraltar*[tiab] OR grenad*[tiab] OR guam*[tiab] OR guatemala*[tiab] OR guinea*[tiab] OR guyan*[tiab] OR guiana*[tiab] OR haiti*[tiab] OR hispaniola[tiab] OR hondura*[tiab] OR hungar*[tiab] OR india*[tiab] OR indonesia*[tiab] OR timor*[tiab] OR iran*[tiab] OR iraq*[tiab] OR jamaica*[tiab] OR jordan*[tiab] OR kazakh*[tiab] OR kenya*[tiab] OR north-korea*[tiab] OR kosov*[tiab] OR kirgiz*[tiab] OR kyrgyz*[tiab] OR kirghiz*[tiab] OR lao[tiab] OR laos[tiab] OR loatian*[tiab] OR latvia*[tiab] OR lebanon*[tiab] OR lesoth*[tiab] OR basutoland*[tiab] OR mosotho*[tiab] OR basotho*[tiab] OR liberia*[tiab] OR libya*[tiab] OR lithuania*[tiab] OR macedonia*[tiab] OR madagasca*[tiab] OR malagasy*[tiab] OR malawi*[tiab] OR nyasaland[tiab] OR malaysia*[tiab] OR maldiv*[tiab] OR mali[tiab] OR malian*[tiab] OR malians[tiab] OR micronesia*[tiab] OR kiribati*[tiab] OR marshall-island*[tiab] OR marshallese[tiab] OR nauru*[tiab] OR mariana*[tiab] OR palau*[tiab] OR tuvalu*[tiab] OR mauritania*[tiab] OR mauritian*[tiab] OR mauritius[tiab] OR mexico[tiab] OR mexican*[tiab] OR moldov*[tiab] OR mongol*[tiab] OR montenegr*[tiab] OR morocc*[tiab] OR ifni[tiab] OR mozambi*[tiab] OR myanma*[tiab] OR burma*[tiab] OR burmese*[tiab] OR namibia*[tiab] OR nepal*[tiab] OR nicaragua*[tiab] OR niger[tiab] OR nigeria*[tiab] OR nigerien*[tiab] OR oman*[tiab] OR muscat[tiab] OR pakistan*[tiab] OR panama*[tiab] OR paraguay*[tiab] OR peru[tiab] OR peruvian*[tiab] OR philippin*[tiab] OR philipin*[tiab] OR phillipin*[tiab] OR phillippin*[tiab] OR filipin*[tiab] OR poland[tiab] OR polish[tiab] OR pole[tiab] OR poles[tiab] OR romania*[tiab] OR russia*[tiab] OR ussr[tiab] OR soviet-union*[tiab] OR union-of-soviet-socialist-republic*[tiab] OR rwand*[tiab] OR ruand*[tiab] OR samoa*[tiab] OR pacific-island*[tiab] OR polynesia*[tiab] OR sao-tome*[tiab] OR senegal*[tiab] OR serbia*[tiab] OR seychell*[tiab] OR sierra-leone*[tiab] OR slovak*[tiab] OR melanesia*[tiab] OR solomon-island*[tiab] OR norfolk-island*[tiab] OR somali*[tiab] OR sri-lanka*[tiab] OR ceylon*[tiab] OR saint-kitt*[tiab] OR st-kitt*[tiab] OR kittitian*[tiab] OR saint-lucia*[tiab] OR st-lucia*[tiab] OR saint-vincent*[tiab] OR st-vincent*[tiab] OR vincentian*[tiab] OR grenadin*[tiab] OR sudan*[tiab] OR surinam*[tiab] OR syria*[tiab] OR tadjik*[tiab] OR tadzhik*[tiab] OR tajik*[tiab] OR tanzania*[tiab] OR tanganyika*[tiab] OR thailand[tiab] OR thai[tiab] OR thais[tiab] OR siam[tiab] OR timor*[tiab] OR togo[tiab] OR togolese*[tiab] OR tonga*[tiab] OR trinidad*[tiab] OR tobago*[tiab] OR tunisia*[tiab] OR turkey[tiab] OR turk[tiab] OR turks[tiab] OR turkmen*[tiab] OR uganda*[tiab] OR ukrain*[tiab] OR uruguay*[tiab] OR uzbek*[tiab] OR vanuatu*[tiab] OR new-hebride*[tiab] OR venezuela*[tiab] OR vietnam*[tiab] OR viet-nam[tiab] OR viet-names*[tiab] OR middle-east*[tiab] OR west-bank[tiab] OR gaza[tiab] OR palestin*[tiab] OR yemen*[tiab] OR yugoslav*[tiab] OR zambia*[tiab] OR zimbabwe*[tiab] OR rhodesia*[tiab] OR global-south[tiab] OR magreb*[tiab] OR maghrib*[tiab] OR sahara*[tiab] OR west-indies[tiab] OR caribbean*[tiab] OR central-america*[tiab] OR latin-america*[tiab] OR south-america*[tiab] OR central-asia*[tiab] OR north-asia*[tiab] OR northern-asia*[tiab] OR southeastern-asia*[tiab] OR south-eastern-asia*[tiab] OR southeast-asia*[tiab] OR south-east-asia*[tiab] OR west-asia*[tiab] OR western-asia*[tiab] OR east-europe*[tiab] OR eastern-europe*[tiab] OR afghan*[ad] OR africa*[ad] OR albania*[ad] OR algeria*[ad] OR angola*[ad] OR argentin*[ad] OR armenia*[ad] OR azerbaijan*[ad] OR bangladesh*[ad] OR belarus[ad] OR belarus*[ad] OR byelarus*[ad] OR belorus*[ad] OR byelorus*[ad] OR belize*[ad] OR benin*[ad] OR dahomey[ad] OR bhutan*[ad] OR bolivia*[ad] OR bosnia*[ad] OR herzegovin*[ad] OR botswana*[ad] OR brazil*[ad] OR brasil*[ad] OR bulgaria*[ad] OR burkina*[ad] OR burundi*[ad] OR cabo-verde*[ad] OR cape-verde*[ad] OR cambodia*[ad] OR kampuchea*[ad] OR khmer*[ad] OR cameroon*[ad] OR cameron*[ad] OR cameroun*[ad] OR ubangi*[tiab] OR chad[ad] OR chadian*[ad] OR chile[ad] OR chilean*[ad] OR china[ad] OR chinese[ad] OR colombia*[ad] OR comoro*[ad] OR mayotte[ad] OR congo[ad] OR congolese[ad] OR zaire*[ad] OR costa-rica*[ad] OR cote-d-ivoir*[ad] OR ivory-coast*[ad] OR ivorian*[ad] OR croatia*[ad] OR cuba*[ad] OR djibouti*[ad] OR dominica*[ad] OR ecuador*[ad] OR egypt*[ad] OR united-arab-republic*[ad] OR salvador*[ad] OR eritrea*[ad] OR estonia*[ad] OR eswatin*[ad] OR swazi*[ad] OR swati*[ad] OR ethiopia*[ad] OR fiji*[ad] OR gabon*[ad] OR gambia*[ad] OR georgia*[ad] OR ghana*[ad] OR gold-coast*[ad] OR gibraltar*[ad] OR grenad*[ad] OR guam*[ad] OR guatemala*[ad] OR guinea*[ad] OR guyan*[ad] OR guiana*[ad] OR haiti*[ad] OR hispaniola[ad] OR hondura*[ad] OR hungar*[ad] OR india*[ad] OR indonesia*[ad] OR timor*[ad] OR iran*[ad] OR iraq*[ad] OR jamaica*[ad] OR jordan*[ad] OR kazakh*[ad] OR kenya*[ad] OR north-korea*[ad] OR kosov*[ad] OR kirgiz*[ad] OR kyrgyz*[ad] OR kirghiz*[ad] OR lao[ad] OR laos[ad] OR latvia*[ad] OR lebanon*[ad] OR lesoth*[ad] OR basotho*[ad] OR liberia*[ad] OR libya*[ad] OR lithuania*[ad] OR macedonia*[ad] OR madagasca*[ad] OR malagasy*[ad] OR malawi*[ad] OR malaysia*[ad] OR maldiv*[ad] OR mali[ad] OR malian[ad] OR malians[ad] OR micronesia*[ad] OR kiribati*[ad] OR marshall-island*[ad] OR marshallese[ad] OR nauru*[ad] OR mariana*[ad] OR palau[ad] OR tuvalu*[ad] OR mauritania*[ad] OR mauritian*[ad] OR mauritius[ad] OR mexico[ad] OR mexican*[ad] OR moldov*[ad] OR mongol*[ad] OR montenegr*[ad] OR morocc*[ad] OR mozambi*[ad] OR myanma*[ad] OR burma*[ad] OR burmese*[ad] OR namibia*[ad] OR nepal*[ad] OR nicaragua*[ad] OR niger[ad] OR nigeria*[ad] OR nigerien*[ad] OR oman*[ad] OR muscat[ad] OR pakistan*[ad] OR panama*[ad] OR paraguay*[ad] OR peru[ad] OR peruvian*[ad] OR philippin*[ad] OR philipin*[ad] OR phillipin*[ad] OR phillippin*[ad] OR filipin*[ad] OR poland[ad] OR polish[ad] OR rhodesia*[ad] OR romania*[ad] OR russia*[ad] OR ussr[ad] OR soviet-union[ad] OR union-of-soviet-socialist-republic*[ad] OR rwand*[ad] OR ruand*[ad] OR samoa*[ad] OR pacific-island*[ad] OR polynesia*[ad] OR sao-tome-and-principe[ad] OR sao-tome*[ad] OR senegal*[ad] OR serbia*[ad] OR seychell*[ad] OR sierra-leone*[ad] OR slovak*[ad] OR melanesia*[ad] OR solomon-island*[ad] OR norfolk-island*[ad] OR sri-lanka*[ad] OR ceylon*[ad] OR saint-kitt*[ad] OR st-kitt*[ad] OR saint-lucia*[ad] OR st-lucia*[ad] OR saint-vincent[ad] OR st-vincent[ad] OR vincentian*[ad] OR grenadin*[ad] OR sudan*[ad] OR surinam*[ad] OR syria*[ad] OR tadjik*[ad] OR tadzhik*[ad] OR tajik*[ad] OR tanzania*[ad] OR tanganyika*[ad] OR thailand[ad] OR thai[ad] OR siam[ad] OR timor*[ad] OR togo[ad] OR togolese*[ad] OR tonga*[ad] OR trinidad*[ad] OR tobago*[ad] OR tunisia*[ad] OR turkey[ad] OR turk[ad] OR turks[ad] OR turkmen*[ad] OR uganda*[ad] OR ukrain*[ad] OR uruguay*[ad] OR uzbek*[ad] OR vanuatu*[ad] OR venezuela*[ad] OR vietnam*[ad] OR viet-nam[ad] OR middle-east*[ad] OR west-bank[ad] OR gaza[ad] OR palestin*[ad] OR yemen*[ad] OR yugoslav*[ad] OR zambia*[ad] OR zimbabwe*[ad] OR global-south[ad] OR sahara*[ad] OR west-indies[ad] OR caribbean*[ad] OR central-america*[ad] OR latin-america*[ad] OR south-america*[ad] OR central-asia*[ad] OR north-asia*[ad] OR northern-asia*[ad] OR southeastern-asia*[ad] OR southeast-asia*[ad] OR south-east-asia*[ad] OR west-asia*[ad] OR western-asia*[ad] OR east-europe*[ad] OR eastern-europe*[ad] OR lmic[tiab] OR lmics[tiab] OR third-world[tiab] OR lami-countr*[tiab] OR transitional-countr*[tiab] OR democratic-people-s-republic-of-korea[tiab] OR ((developing[tiab] OR less-developed[tiab] OR lesser-developed[tiab] OR under-developed[tiab] OR underdeveloped[tiab] OR middle-income[tiab] OR low-income[tiab] OR lower-income[tiab] OR underserved[tiab] OR under-served[tiab] OR deprived[tiab] OR poor[tiab] OR poorer[tiab] OR improverished[tiab]) AND (countr*[tiab] OR nation*[tiab] OR population*[tiab] OR world*[tiab] OR econom*[tiab])) OR ((low[tiab] OR lower[tiab]) AND (gdp[tiab] OR gnp[tiab] OR gross-domestic[tiab] OR gross-national[tiab])) OR ((emerging[tiab]) AND (econom*[tiab] OR nation*[tiab])))) ("Liver Neoplasms"[mesh] OR "Hepatoblastoma"[mesh] OR "Biliary Tract Neoplasms"[mesh] OR "Cholangiocarcinoma"[mesh] OR (hepatoblastoma*[tiab] OR hepatocell*[tiab] OR HCC[tiab] OR cholangiocarcinoma*[tiab] OR hepatocholangiocarcinoma*[tiab] OR CHCC[tiab] OR (("Liver"[mesh] OR "Biliary Tract"[mesh] OR (liver*[tiab] OR hepatic*[tiab] OR biliary[tiab] OR hepatobiliary[tiab] OR hepatopancrea*[tiab] OR pancreatobiliary[tiab] OR bile-canalicul*[tiab] OR gallbladder*[tiab] OR cholangiocellular*[tiab] OR klatskin*[tiab] OR bile-duct*[tiab])) AND (neoplas*[tiab] OR cancer*[tiab] OR adenoma*[tiab] OR carcinoma*[tiab] OR adenocarcinoma*[tiab] OR malignan*[tiab] OR tumor*[tiab] OR tumour*[tiab] OR metasta*[tiab] OR lesion*[tiab] OR mass*[tiab])))) AND ("Hepatectomy"[mesh] OR "Metastasectomy"[mesh] OR "Cholecystectomy"[mesh] OR (hepatectom*[tiab] OR posthepatectom*[tiab] OR post-hepatectom*[tiab] OR hepathectom*[tiab] OR hepatopancreatoduodenectom*[tiab] OR metastasectom*[tiab] OR postmetastasectom*[tiab] OR post-metastasectom*[tiab] OR cholecystectom*[tiab] OR lobectom*[tiab] OR postlobectom*[tiab] OR post-lobectom*[tiab] OR surger*[tiab] OR surgery[sh] OR surgical*[tiab] OR resection*[tiab] OR debulk*[tiab])) AND

(“afghanistan”[mesh] OR “albania”[mesh] OR “algeria”[mesh] OR “american samoa”[mesh] OR “angola”[mesh] OR “argentina”[mesh] OR “armenia”[mesh] OR “azerbaijan”[mesh] OR “bangladesh”[mesh] OR “republic of belarus”[mesh] OR “belize”[mesh] OR “benin”[mesh] OR “bhutan”[mesh] OR “bolivia”[mesh] OR “bosnia and herzegovina”[mesh] OR “botswana”[mesh] OR “brazil”[mesh] OR “bulgaria”[mesh] OR “burkina faso”[mesh] OR “burundi”[mesh] OR “cabo verde”[mesh] OR “cambodia”[mesh] OR “cameroon”[mesh] OR “central african republic”[mesh] OR “chad”[mesh] OR “chile”[mesh] OR “china”[mesh] OR “colombia”[mesh] OR “comoros”[mesh] OR “democratic republic of the congo”[mesh] OR “congo”[mesh] OR “costa rica”[mesh] OR “cote d’ivoire”[mesh] OR “croatia”[mesh] OR “cuba”[mesh] OR “djibouti”[mesh] OR “dominica”[mesh] OR “dominican republic”[mesh] OR “ecuador”[mesh] OR “egypt”[mesh] OR “el salvador”[mesh] OR “equatorial guinea”[mesh] OR “eritrea”[mesh] OR “estonia”[mesh] OR "Eswatini"[Mesh] OR “ethiopia”[mesh] OR “fiji”[mesh] OR “gabon”[mesh] OR “gambia”[mesh] OR “georgia (republic)“[mesh] OR “ghana”[mesh] OR “gibraltar”[mesh] OR “grenada”[mesh] OR “guam”[mesh] OR “guatemala”[mesh] OR “guinea”[mesh] OR “guinea-bissau”[mesh] OR “guyana”[mesh] OR “haiti”[mesh] OR “honduras”[mesh] OR “hungary”[mesh] OR “india”[mesh] OR “indonesia”[mesh] OR “iran”[mesh] OR “iraq”[mesh] OR “jamaica”[mesh] OR “jordan”[mesh] OR “kazakhstan”[mesh] OR “kenya”[mesh] OR “democratic people’s republic of korea”[mesh] OR “kosovo”[mesh] OR “kyrgyzstan”[mesh] OR “laos”[mesh] OR “latvia”[mesh] OR “lebanon”[mesh] OR “lesotho”[mesh] OR “liberia”[mesh] OR “libya”[mesh] OR “lithuania”[mesh] OR “republic of north macedonia”[mesh] OR “madagascar”[mesh] OR “malawi”[mesh] OR “malaysia”[mesh] OR “indian ocean islands”[mesh] OR “mali”[mesh] OR “micronesia”[mesh] OR “palau”[mesh] OR “mauritania”[mesh] OR “mauritius”[mesh] OR “mexico”[mesh] OR “moldova”[mesh] OR “mongolia”[mesh] OR “montenegro”[mesh] OR “morocco”[mesh] OR “mozambique”[mesh] OR “myanmar”[mesh] OR “namibia”[mesh] OR “nepal”[mesh] OR “nicaragua”[mesh] OR “niger”[mesh] OR “nigeria”[mesh] OR “oman”[mesh] OR “pakistan”[mesh] OR “panama”[mesh] OR “papua new guinea”[mesh] OR “paraguay”[mesh] OR “peru”[mesh] OR “philippines”[mesh] OR “poland”[mesh] OR “romania”[mesh] OR “russia”[mesh] OR “rwanda”[mesh] OR “samoa”[mesh] OR “sao tome and principe”[mesh] OR “senegal”[mesh] OR “serbia”[mesh] OR “seychelles”[mesh] OR “sierra leone”[mesh] OR “slovakia”[mesh] OR “slovenia”[mesh] OR “melanesia”[mesh] OR “somalia”[mesh] OR “south africa”[mesh] OR “south sudan”[mesh] OR “sri lanka”[mesh] OR “saint kitts and nevis”[mesh] OR “saint lucia”[mesh] OR “saint vincent and the grenadines”[mesh] OR “sudan”[mesh] OR “suriname”[mesh] OR “syria”[mesh] OR “tajikistan”[mesh] OR “tanzania”[mesh] OR “thailand”[mesh] OR “timor-leste”[mesh] OR “togo”[mesh] OR “tonga”[mesh] OR “trinidad and tobago”[mesh] OR “tunisia”[mesh] OR “turkey”[mesh] OR “turkmenistan”[mesh] OR “uganda”[mesh] OR “ukraine”[mesh] OR “uruguay”[mesh] OR “uzbekistan”[mesh] OR “vanuatu”[mesh] OR “venezuela”[mesh] OR “vietnam”[mesh] OR “middle east”[mesh] OR “yemen”[mesh] OR “yugoslavia”[mesh] OR “zambia”[mesh] OR “zimbabwe”[mesh] OR “africa south of the sahara”[mesh] OR “africa, central”[mesh] OR “africa, northern”[mesh] OR “africa, southern”[mesh] OR “africa, eastern”[mesh] OR “africa, western”[mesh] OR “west indies”[mesh] OR “indian ocean islands”[mesh] OR “caribbean region”[mesh] OR “central america”[mesh] OR “latin america”[mesh] OR “south america”[mesh] OR “asia, central”[mesh] OR “asia, northern”[mesh] OR “asia, southeastern”[mesh] OR “asia, western”[mesh] OR “europe, eastern”[mesh] OR “developing countries”[mesh] OR (afghan*[tiab] OR africa*[tiab] OR albania*[tiab] OR algeria*[tiab] OR angola*[tiab] OR argentin*[tiab] OR armenia*[tiab] OR azerbaijan*[tiab] OR bangladesh*[tiab] OR bangalee*[tiab] OR bajan*[tiab] OR belarus*[tiab] OR byelarus*[tiab] OR belorus*[tiab] OR byelorus*[tiab] OR belize*[tiab] OR benin*[tiab] OR dahomey[tiab] OR bhutan*[tiab] OR bolivia*[tiab] OR bosnia*[tiab] OR herzegovin*[tiab] OR botswana*[tiab] OR batswana*[tiab] OR bechuanaland*[tiab] OR brazil*[tiab] OR brasil*[tiab] OR bulgaria*[tiab] OR burkina*[tiab] OR upper-volta[tiab] OR burundi*[tiab] OR urundi*[tiab] OR cabo-verde*[tiab] OR cape-verde*[tiab] OR cambodia*[tiab] OR kampuchea*[tiab] OR khmer*[tiab] OR cameroon*[tiab] OR cameron*[tiab] OR cameroun*[tiab] OR ubangi*[tiab] OR chad[tiab] OR chadian*[tiab] OR chile[tiab] OR chilean*[tiab] OR china[tiab] OR chinese[tiab] OR colombia*[tiab] OR comoro*[tiab] OR comorian*[tiab] OR mayotte*[tiab] OR congo[tiab] OR congolese[tiab] OR zaire*[tiab] OR costa-rica*[tiab] OR cote-d-ivoir*[tiab] OR ivory-coast*[tiab] OR ivorian*[tiab] OR croatia*[tiab] OR cuba*[tiab] OR djibouti*[tiab] OR dominica*[tiab] OR ecuador*[tiab] OR egypt*[tiab] OR united-arab-republic*[tiab] OR salvador*[tiab] OR equatoguinean*[tiab] OR eritrea*[tiab] OR estonia*[tiab] OR eswatin*[tiab] OR swazi*[tiab] OR swati*[tiab] OR ethiopia*[tiab] OR fiji*[tiab] OR gabon*[tiab] OR gambia*[tiab] OR georgia*[tiab] OR ghana*[tiab] OR gold-coast*[tiab] OR gibraltar*[tiab] OR grenad*[tiab] OR guam*[tiab] OR guatemala*[tiab] OR guinea*[tiab] OR guyan*[tiab] OR guiana*[tiab] OR haiti*[tiab] OR hispaniola[tiab] OR hondura*[tiab] OR hungar*[tiab] OR india*[tiab] OR indonesia*[tiab] OR timor*[tiab] OR iran*[tiab] OR iraq*[tiab] OR jamaica*[tiab] OR jordan*[tiab] OR kazakh*[tiab] OR kenya*[tiab] OR north-korea*[tiab] OR kosov*[tiab] OR kirgiz*[tiab] OR kyrgyz*[tiab] OR kirghiz*[tiab] OR lao[tiab] OR laos[tiab] OR loatian*[tiab] OR latvia*[tiab] OR lebanon*[tiab] OR lesoth*[tiab] OR basutoland*[tiab] OR mosotho*[tiab] OR basotho*[tiab] OR liberia*[tiab] OR libya*[tiab] OR lithuania*[tiab] OR macedonia*[tiab] OR madagasca*[tiab] OR malagasy*[tiab] OR malawi*[tiab] OR nyasaland[tiab] OR malaysia*[tiab] OR maldiv*[tiab] OR mali[tiab] OR malian*[tiab] OR malians[tiab] OR micronesia*[tiab] OR kiribati*[tiab] OR marshall-island*[tiab] OR marshallese[tiab] OR nauru*[tiab] OR mariana*[tiab] OR palau*[tiab] OR tuvalu*[tiab] OR mauritania*[tiab] OR mauritian*[tiab] OR mauritius[tiab] OR mexico[tiab] OR mexican*[tiab] OR moldov*[tiab] OR mongol*[tiab] OR montenegr*[tiab] OR morocc*[tiab] OR ifni[tiab] OR mozambi*[tiab] OR myanma*[tiab] OR burma*[tiab] OR burmese*[tiab] OR namibia*[tiab] OR nepal*[tiab] OR nicaragua*[tiab] OR niger[tiab] OR nigeria*[tiab] OR nigerien*[tiab] OR oman*[tiab] OR muscat[tiab] OR pakistan*[tiab] OR panama*[tiab] OR paraguay*[tiab] OR peru[tiab] OR peruvian*[tiab] OR philippin*[tiab] OR philipin*[tiab] OR phillipin*[tiab] OR phillippin*[tiab] OR filipin*[tiab] OR poland[tiab] OR polish[tiab] OR pole[tiab] OR poles[tiab] OR romania*[tiab] OR russia*[tiab] OR ussr[tiab] OR soviet-union*[tiab] OR union-of-soviet-socialist-republic*[tiab] OR rwand*[tiab] OR ruand*[tiab] OR samoa*[tiab] OR pacific-island*[tiab] OR polynesia*[tiab] OR sao-tome*[tiab] OR senegal*[tiab] OR serbia*[tiab] OR seychell*[tiab] OR sierra-leone*[tiab] OR slovak*[tiab] OR melanesia*[tiab] OR solomon-island*[tiab] OR norfolk-island*[tiab] OR somali*[tiab] OR sri-lanka*[tiab] OR ceylon*[tiab] OR saint-kitt*[tiab] OR st-kitt*[tiab] OR kittitian*[tiab] OR saint-lucia*[tiab] OR st-lucia*[tiab] OR saint-vincent*[tiab] OR st-vincent*[tiab] OR vincentian*[tiab] OR grenadin*[tiab] OR sudan*[tiab] OR surinam*[tiab] OR syria*[tiab] OR tadjik*[tiab] OR tadzhik*[tiab] OR tajik*[tiab] OR tanzania*[tiab] OR tanganyika*[tiab] OR thailand[tiab] OR thai[tiab] OR thais[tiab] OR siam[tiab] OR timor*[tiab] OR togo[tiab] OR togolese*[tiab] OR tonga*[tiab] OR trinidad*[tiab] OR tobago*[tiab] OR tunisia*[tiab] OR turkey[tiab] OR turk[tiab] OR turks[tiab] OR turkmen*[tiab] OR uganda*[tiab] OR ukrain*[tiab] OR uruguay*[tiab] OR uzbek*[tiab] OR vanuatu*[tiab] OR new-hebride*[tiab] OR venezuela*[tiab] OR vietnam*[tiab] OR viet-nam[tiab] OR viet-names*[tiab] OR middle-east*[tiab] OR west-bank[tiab] OR gaza[tiab] OR palestin*[tiab] OR yemen*[tiab] OR yugoslav*[tiab] OR zambia*[tiab] OR zimbabwe*[tiab] OR rhodesia*[tiab] OR global-south[tiab] OR magreb*[tiab] OR maghrib*[tiab] OR sahara*[tiab] OR west-indies[tiab] OR caribbean*[tiab] OR central-america*[tiab] OR latin-america*[tiab] OR south-america*[tiab] OR central-asia*[tiab] OR north-asia*[tiab] OR northern-asia*[tiab] OR southeastern-asia*[tiab] OR south-eastern-asia*[tiab] OR southeast-asia*[tiab] OR south-east-asia*[tiab] OR west-asia*[tiab] OR western-asia*[tiab] OR east-europe*[tiab] OR eastern-europe*[tiab] OR afghan*[ad] OR africa*[ad] OR albania*[ad] OR algeria*[ad] OR angola*[ad] OR argentin*[ad] OR armenia*[ad] OR azerbaijan*[ad] OR bangladesh*[ad] OR belarus[ad] OR belarus*[ad] OR byelarus*[ad] OR belorus*[ad] OR byelorus*[ad] OR belize*[ad] OR benin*[ad] OR dahomey[ad] OR bhutan*[ad] OR bolivia*[ad] OR bosnia*[ad] OR herzegovin*[ad] OR botswana*[ad] OR brazil*[ad] OR brasil*[ad] OR bulgaria*[ad] OR burkina*[ad] OR burundi*[ad] OR cabo-verde*[ad] OR cape-verde*[ad] OR cambodia*[ad] OR kampuchea*[ad] OR khmer*[ad] OR cameroon*[ad] OR cameron*[ad] OR cameroun*[ad] OR ubangi*[tiab] OR chad[ad] OR chadian*[ad] OR chile[ad] OR chilean*[ad] OR china[ad] OR chinese[ad] OR colombia*[ad] OR comoro*[ad] OR mayotte[ad] OR congo[ad] OR congolese[ad] OR zaire*[ad] OR costa-rica*[ad] OR cote-d-ivoir*[ad] OR ivory-coast*[ad] OR ivorian*[ad] OR croatia*[ad] OR cuba*[ad] OR djibouti*[ad] OR dominica*[ad] OR ecuador*[ad] OR egypt*[ad] OR united-arab-republic*[ad] OR salvador*[ad] OR eritrea*[ad] OR estonia*[ad] OR eswatin*[ad] OR swazi*[ad] OR swati*[ad] OR ethiopia*[ad] OR fiji*[ad] OR gabon*[ad] OR gambia*[ad] OR georgia*[ad] OR ghana*[ad] OR gold-coast*[ad] OR gibraltar*[ad] OR grenad*[ad] OR guam*[ad] OR guatemala*[ad] OR guinea*[ad] OR guyan*[ad] OR guiana*[ad] OR haiti*[ad] OR hispaniola[ad] OR hondura*[ad] OR hungar*[ad] OR india*[ad] OR indonesia*[ad] OR timor*[ad] OR iran*[ad] OR iraq*[ad] OR jamaica*[ad] OR jordan*[ad] OR kazakh*[ad] OR kenya*[ad] OR north-korea*[ad] OR kosov*[ad] OR kirgiz*[ad] OR kyrgyz*[ad] OR kirghiz*[ad] OR lao[ad] OR laos[ad] OR latvia*[ad] OR lebanon*[ad] OR lesoth*[ad] OR basotho*[ad] OR liberia*[ad] OR libya*[ad] OR lithuania*[ad] OR macedonia*[ad] OR madagasca*[ad] OR malagasy*[ad] OR malawi*[ad] OR malaysia*[ad] OR maldiv*[ad] OR mali[ad] OR malian[ad] OR malians[ad] OR micronesia*[ad] OR kiribati*[ad] OR marshall-island*[ad] OR marshallese[ad] OR nauru*[ad] OR mariana*[ad] OR palau[ad] OR tuvalu*[ad] OR mauritania*[ad] OR mauritian*[ad] OR mauritius[ad] OR mexico[ad] OR mexican*[ad] OR moldov*[ad] OR mongol*[ad] OR montenegr*[ad] OR morocc*[ad] OR mozambi*[ad] OR myanma*[ad] OR burma*[ad] OR burmese*[ad] OR namibia*[ad] OR nepal*[ad] OR nicaragua*[ad] OR niger[ad] OR nigeria*[ad] OR nigerien*[ad] OR oman*[ad] OR muscat[ad] OR pakistan*[ad] OR panama*[ad] OR paraguay*[ad] OR peru[ad] OR peruvian*[ad] OR philippin*[ad] OR philipin*[ad] OR phillipin*[ad] OR phillippin*[ad] OR filipin*[ad] OR poland[ad] OR polish[ad] OR rhodesia*[ad] OR romania*[ad] OR russia*[ad] OR ussr[ad] OR soviet-union[ad] OR union-of-soviet-socialist-republic*[ad] OR rwand*[ad] OR ruand*[ad] OR samoa*[ad] OR pacific-island*[ad] OR polynesia*[ad] OR sao-tome-and-principe[ad] OR sao-tome*[ad] OR senegal*[ad] OR serbia*[ad] OR seychell*[ad] OR sierra-leone*[ad] OR slovak*[ad] OR melanesia*[ad] OR solomon-island*[ad] OR norfolk-island*[ad] OR sri-lanka*[ad] OR ceylon*[ad] OR saint-kitt*[ad] OR st-kitt*[ad] OR saint-lucia*[ad] OR st-lucia*[ad] OR saint-vincent[ad] OR st-vincent[ad] OR vincentian*[ad] OR grenadin*[ad] OR sudan*[ad] OR surinam*[ad] OR syria*[ad] OR tadjik*[ad] OR tadzhik*[ad] OR tajik*[ad] OR tanzania*[ad] OR tanganyika*[ad] OR thailand[ad] OR thai[ad] OR siam[ad] OR timor*[ad] OR togo[ad] OR togolese*[ad] OR tonga*[ad] OR trinidad*[ad] OR tobago*[ad] OR tunisia*[ad] OR turkey[ad] OR turk[ad] OR turks[ad] OR turkmen*[ad] OR uganda*[ad] OR ukrain*[ad] OR uruguay*[ad] OR uzbek*[ad] OR vanuatu*[ad] OR venezuela*[ad] OR vietnam*[ad] OR viet-nam[ad] OR middle-east*[ad] OR west-bank[ad] OR gaza[ad] OR palestin*[ad] OR yemen*[ad] OR yugoslav*[ad] OR zambia*[ad] OR zimbabwe*[ad] OR global-south[ad] OR sahara*[ad] OR west-indies[ad] OR caribbean*[ad] OR central-america*[ad] OR latin-america*[ad] OR south-america*[ad] OR central-asia*[ad] OR north-asia*[ad] OR northern-asia*[ad] OR southeastern-asia*[ad] OR southeast-asia*[ad] OR south-east-asia*[ad] OR west-asia*[ad] OR western-asia*[ad] OR east-europe*[ad] OR eastern-europe*[ad] OR lmic[tiab] OR lmics[tiab] OR third-world[tiab] OR lami-countr*[tiab] OR transitional-countr*[tiab] OR democratic-people-s-republic-of-korea[tiab] OR ((developing[tiab] OR less-developed[tiab] OR lesser-developed[tiab] OR under-developed[tiab] OR underdeveloped[tiab] OR middle-income[tiab] OR low-income[tiab] OR lower-income[tiab] OR underserved[tiab] OR under-served[tiab] OR deprived[tiab] OR poor[tiab] OR poorer[tiab] OR improverished[tiab]) AND (countr*[tiab] OR nation*[tiab] OR population*[tiab] OR world*[tiab] OR econom*[tiab])) OR ((low[tiab] OR lower[tiab]) AND (gdp[tiab] OR gnp[tiab] OR gross-domestic[tiab] OR gross-national[tiab])) OR ((emerging[tiab]) AND (econom*[tiab] OR nation*[tiab])))) NOT ("Animals"[Mesh] NOT ("Animals"[Mesh] AND "Humans"[Mesh])) NOT ("Editorial"[pt] OR "Comment"[pt] OR “Clinical Trial Protocol”[pt] OR case-report*[ti] OR systematic-review*[ti] OR meta-analys*[ti]) AND (2005/1/1:3000[pdat])

*** * ***

**Embase (Scopus): 14,197 Results**

(TITLE-ABS-KEY(hepatoblastoma* OR hepatocell* OR HCC OR cholangiocarcinoma* OR hepatocholangiocarcinoma* OR CHCC* OR ((liver* OR hepatic* OR biliary OR hepatobiliary OR hepatopancrea* OR pancreatobiliary OR bile-canalicul* OR gallbladder* OR cholangiocellular* OR klatskin* OR bile-duct*) W/3 (neoplas* OR cancer* OR adenoma* OR carcinoma* OR adenocarcinoma* OR malignan* OR tumor* OR tumour* OR metasta* OR lesion* OR mass*))) AND TITLE-ABS-KEY(hepatectom* OR posthepatectom* OR post-hepatectom* OR hepathectom* OR hepatopancreatoduodenectom* OR metastasectom* OR postmetastasectom* OR post-metastasectom* OR cholecystectom* OR lobectom* OR postlobectom* OR post-lobectom* OR surger* OR surgical* OR resection* OR debulk*)) AND ((TITLE-ABS-KEY(afghan* OR africa* OR albania* OR algeria* OR angola* OR argentin* OR armenia* OR azerbaijan* OR bangladesh* OR bangalee* OR bajan* OR belarus* OR byelarus* OR belorus* OR byelorus* OR belize* OR benin* OR dahomey OR bhutan* OR bolivia* OR bosnia* OR herzegovin* OR botswana* OR batswana* OR bechuanaland* OR brazil* OR brasil* OR bulgaria* OR burkina* OR upper-volta OR burundi* OR urundi* OR cabo-verde* OR cape-verde* OR cambodia* OR kampuchea* OR khmer* OR cameroon* OR cameron* OR cameroun* OR ubangi* OR chad OR chadian* OR chile OR chilean* OR china OR chinese OR colombia* OR comoro* OR comorian* OR mayotte* OR congo OR congolese OR zaire* OR costa-rica* OR cote-d-ivoir* OR ivory-coast* OR ivorian* OR croatia* OR cuba* OR djibouti* OR dominica* OR ecuador* OR egypt* OR united-arab-republic* OR salvador* OR equatoguinean* OR eritrea* OR estonia* OR eswatin* OR swazi* OR swati* OR ethiopia* OR fiji* OR gabon* OR gambia* OR georgia* OR ghana* OR gold-coast* OR gibraltar* OR grenad* OR guam* OR guatemala* OR guinea* OR guyan* OR guiana* OR haiti* OR hispaniola OR hondura* OR hungar* OR india* OR indonesia* OR timor* OR iran* OR iraq* OR jamaica* OR jordan* OR kazakh* OR kenya* OR north-korea* OR kosov* OR kyrgyz* OR kirghiz* OR kirgiz* OR kirghiz* OR lao OR laos OR loatian* OR latvia* OR lebanon* OR lesoth* OR basutoland OR mosotho* OR basotho* OR liberia* OR libya* OR lithuania* OR macedonia* OR madagasca* OR malagasy* OR malawi* OR nyasaland OR malaysia* OR maldiv* OR mali OR malian OR malians OR micronesia* OR kiribati* OR marshall-island* OR marshallese OR nauru* OR mariana* OR palau* OR tuvalu* OR mauritania* OR mauritian* OR mauritius OR mexico OR mexican* OR moldov* OR mongol* OR montenegr* OR morocc* OR ifni OR mozambi* OR myanma* OR burma* OR burmese* OR namibia* OR nauruan* OR nepal* OR nicaragua* OR niger OR nigeria* OR nigerien* OR oman* OR muscat OR pakistan* OR panama* OR paraguay* OR peru OR peruvian* OR philippin* OR philipin* OR phillipin* OR phillippin* OR filipin* OR poland OR polish OR pole OR poles OR romania* OR russia* OR ussr OR soviet-union* OR union-of-soviet-socialist-republic* OR rwand* OR ruand* OR samoa* OR pacific-island* OR polynesia* OR sao-tome* OR senegal* OR serbia* OR seychell* OR sierra-leone* OR slovak* OR melanesia* OR solomon-island* OR norfolk-island* OR somali* OR sri-lanka* OR ceylon* OR saint-kitt* OR st-kitt* OR kittitian* OR saint-lucia* OR st-lucia* OR saint-vincent* OR st-vincent* OR vincentian* OR grenadin* OR sudan* OR surinam* OR syria* OR tadjik* OR tadzhik* OR tajik* OR tanzania* OR tanganyika* OR thailand* OR thai OR thais OR siam OR timor* OR togo OR togolese* OR tonga* OR trinidad* OR tobago* OR tunisia* OR turkey OR turk OR turks OR turkmen* OR uganda* OR ukrain* OR uruguay* OR uzbek* OR vanuatu* OR new-hebride* OR venezuela* OR vietnam* OR viet-nam OR viet-names* OR middle-east* OR west-bank OR gaza OR palestin* OR yemen* OR yugoslav* OR zambia* OR zimbabwe* OR rhodesia* OR global-south OR magreb* OR maghrib* OR sahara* OR west-indies OR caribbean* OR central-america* OR latin-america* OR south-america* OR central-asia* OR north-asia* OR northern-asia* OR southeastern-asia* OR south-eastern-asia* OR southeast-asia* OR south-east-asia* OR west-asia* OR western-asia* OR east-europe* OR eastern-europe* OR lmic OR lmics OR third-world OR lami-countr* OR transitional-countr* OR ((developing OR less-developed OR lesser-developed OR under-developed OR underdeveloped OR middle-income OR low-income OR lower-income OR underserved OR under-served OR deprived OR poor OR poorer) W/3 (countr* OR nation* OR population* OR world* OR econom*)) OR ((low OR lower) W/3 (gdp OR gnp OR gross-domestic OR gross-national)) OR ((emerging) W/3 (econom* OR nation*)))) OR (AFFIL(afghan* OR africa* OR albania* OR algeria* OR angola* OR argentin* OR armenia* OR azerbaijan* OR bangladesh* OR bangalee* OR bajan* OR belarus* OR byelarus* OR belorus* OR byelorus* OR belize* OR benin* OR dahomey OR bhutan* OR bolivia* OR bosnia* OR herzegovin* OR botswana* OR batswana* OR bechuanaland* OR brazil* OR brasil* OR bulgaria* OR burkina* OR upper-volta OR burundi* OR urundi* OR cabo-verde* OR cape-verde* OR cambodia* OR kampuchea* OR khmer* OR cameroon* OR cameron* OR cameroun* OR ubangi* OR chad OR chadian* OR chile OR chilean* OR china OR chinese OR colombia* OR comoro* OR comorian* OR mayotte* OR congo OR congolese OR zaire* OR costa-rica* OR cote-d-ivoir* OR ivory-coast* OR ivorian* OR croatia* OR cuba* OR djibouti* OR dominica* OR ecuador* OR egypt* OR united-arab-republic* OR salvador* OR equatoguinean* OR eritrea* OR estonia* OR eswatin* OR swazi* OR swati* OR ethiopia* OR fiji* OR gabon* OR gambia* OR georgia* OR ghana* OR gold-coast* OR gibraltar* OR grenad* OR guam* OR guatemala* OR guinea* OR guyan* OR guiana* OR haiti* OR hispaniola OR hondura* OR hungar* OR india* OR indonesia* OR timor* OR iran* OR iraq* OR jamaica* OR jordan* OR kazakh* OR kenya* OR north-korea* OR kosov* OR kyrgyz* OR kirghiz* OR kirgiz* OR kirghiz* OR lao OR laos OR loatian* OR latvia* OR lebanon* OR lesoth* OR basutoland OR mosotho* OR basotho* OR liberia* OR libya* OR lithuania* OR macedonia* OR madagasca* OR malagasy* OR malawi* OR nyasaland OR malaysia* OR maldiv* OR mali OR malian OR malians OR micronesia* OR kiribati* OR marshall-island* OR marshallese OR nauru* OR mariana* OR palau* OR tuvalu* OR mauritania* OR mauritian* OR mauritius OR mexico OR mexican* OR moldov* OR mongol* OR montenegr* OR morocc* OR ifni OR mozambi* OR myanma* OR burma* OR burmese* OR namibia* OR nauruan* OR nepal* OR nicaragua* OR niger OR nigeria* OR nigerien* OR oman* OR muscat OR pakistan* OR panama* OR paraguay* OR peru OR peruvian* OR philippin* OR philipin* OR phillipin* OR phillippin* OR filipin* OR poland OR polish OR pole OR poles OR romania* OR russia* OR ussr OR soviet-union* OR union-of-soviet-socialist-republic* OR rwand* OR ruand* OR samoa* OR pacific-island* OR polynesia* OR sao-tome* OR senegal* OR serbia* OR seychell* OR sierra-leone* OR slovak* OR melanesia* OR solomon-island* OR norfolk-island* OR somali* OR sri-lanka* OR ceylon* OR saint-kitt* OR st-kitt* OR kittitian* OR saint-lucia* OR st-lucia* OR saint-vincent* OR st-vincent* OR vincentian* OR grenadin* OR sudan* OR surinam* OR syria* OR tadjik* OR tadzhik* OR tajik* OR tanzania* OR tanganyika* OR thailand* OR thai OR siam OR timor* OR togo OR togolese* OR tonga* OR trinidad* OR tobago* OR tunisia* OR turkey OR turk OR turks OR turkmen* OR uganda* OR ukrain* OR uruguay* OR uzbek* OR vanuatu* OR new-hebride* OR venezuela* OR vietnam* OR viet-nam OR viet-names* OR middle-east* OR west-bank OR gaza OR palestin* OR yemen* OR yugoslav* OR zambia* OR zimbabwe* OR rhodesia* OR global-south OR magreb* OR maghrib* OR sahara* OR west-indies OR caribbean* OR central-america* OR latin-america* OR south-america* OR central-asia* OR north-asia* OR northern-asia* OR southeastern-asia* OR south-eastern-asia* OR southeast-asia* OR south-east-asia* OR west-asia* OR western-asia* OR east-europe* OR eastern-europe*))) AND (PUBYEAR AFT 2004) AND (INDEX(embase)) AND NOT (KEY((animal* OR nonhuman* OR rat OR rats OR mouse OR mice OR rodent* OR murine* OR primate* OR monkey* OR dog OR dogs OR canine* OR pig* OR porcine*) AND NOT (human* AND (animal* OR nonhuman* OR rat OR rats OR mouse OR mice OR rodent* OR murine* OR primate* OR monkey* OR dog OR dogs OR canine* OR pig* OR porcine*)))) AND NOT (TITLE(editorial* OR comment* OR case-report* OR systematic-review* OR meta-analys* OR ((trial*) AND (protocol*))))

*** * ***

**Science Citation Index Expanded, Emerging Sources Citation Index (Web of Science): 11,311 Results**

(TS=(hepatoblastoma* OR hepatocell* OR HCC OR cholangiocarcinoma* OR hepatocholangiocarcinoma* OR CHCC* OR ((liver* OR hepatic* OR biliary OR hepatobiliary OR hepatopancrea* OR pancreatobiliary OR bile-canalicul* OR gallbladder* OR cholangiocellular* OR klatskin* OR bile-duct*) NEAR/3 (neoplas* OR cancer* OR adenoma* OR carcinoma* OR adenocarcinoma* OR malignan* OR tumor* OR tumour* OR metasta* OR lesion* OR mass*))) AND TS=(hepatectom* OR posthepatectom* OR post-hepatectom* OR hepathectom* OR hepatopancreatoduodenectom* OR metastasectom* OR postmetastasectom* OR post-metastasectom* OR cholecystectom* OR lobectom* OR postlobectom* OR post-lobectom* OR surger* OR surgical* OR resection* OR debulk*)) AND ((TS=(afghan* OR africa* OR albania* OR algeria* OR angola* OR argentin* OR armenia* OR azerbaijan* OR bangladesh* OR bangalee* OR bajan* OR belarus* OR byelarus* OR belorus* OR byelorus* OR belize* OR benin* OR dahomey OR bhutan* OR bolivia* OR bosnia* OR herzegovin* OR botswana* OR batswana* OR bechuanaland* OR brazil* OR brasil* OR bulgaria* OR burkina* OR upper-volta OR burundi* OR urundi* OR cabo-verde* OR cape-verde* OR cambodia* OR kampuchea* OR khmer* OR cameroon* OR cameron* OR cameroun* OR ubangi* OR chad OR chadian* OR chile OR chilean* OR china OR chinese OR colombia* OR comoro* OR comorian* OR mayotte* OR congo OR congolese OR zaire* OR costa-rica* OR cote-d-ivoir* OR ivory-coast* OR ivorian* OR croatia* OR cuba* OR djibouti* OR dominica* OR ecuador* OR egypt* OR united-arab-republic* OR salvador* OR equatoguinean* OR eritrea* OR estonia* OR eswatin* OR swazi* OR swati* OR ethiopia* OR fiji* OR gabon* OR gambia* OR georgia* OR ghana* OR gold-coast* OR gibraltar* OR grenad* OR guam* OR guatemala* OR guinea* OR guyan* OR guiana* OR haiti* OR hispaniola OR hondura* OR hungar* OR india* OR indonesia* OR timor* OR iran* OR iraq* OR jamaica* OR jordan* OR kazakh* OR kenya* OR north-korea* OR kosov* OR kyrgyz* OR kirghiz* OR kirgiz* OR kirghiz* OR lao OR laos OR loatian* OR latvia* OR lebanon* OR lesoth* OR basutoland OR mosotho* OR basotho* OR liberia* OR libya* OR lithuania* OR macedonia* OR madagasca* OR malagasy* OR malawi* OR nyasaland OR malaysia* OR maldiv* OR mali OR malian OR malians OR micronesia* OR kiribati* OR marshall-island* OR marshallese OR nauru* OR mariana* OR palau* OR tuvalu* OR mauritania* OR mauritian* OR mauritius OR mexico OR mexican* OR moldov* OR mongol* OR montenegr* OR morocc* OR ifni OR mozambi* OR myanma* OR burma* OR burmese* OR namibia* OR nauruan* OR nepal* OR nicaragua* OR niger OR nigeria* OR nigerien* OR oman* OR muscat OR pakistan* OR panama* OR paraguay* OR peru OR peruvian* OR philippin* OR philipin* OR phillipin* OR phillippin* OR filipin* OR poland OR polish OR pole OR poles OR romania* OR russia* OR ussr OR soviet-union* OR union-of-soviet-socialist-republic* OR rwand* OR ruand* OR samoa* OR pacific-island* OR polynesia* OR sao-tome* OR senegal* OR serbia* OR seychell* OR sierra-leone* OR slovak* OR melanesia* OR solomon-island* OR norfolk-island* OR somali* OR sri-lanka* OR ceylon* OR saint-kitt* OR st-kitt* OR kittitian* OR saint-lucia* OR st-lucia* OR saint-vincent* OR st-vincent* OR vincentian* OR grenadin* OR sudan* OR surinam* OR syria* OR tadjik* OR tadzhik* OR tajik* OR tanzania* OR tanganyika* OR thailand* OR thai OR thais OR siam OR timor* OR togo OR togolese* OR tonga* OR trinidad* OR tobago* OR tunisia* OR turkey OR turk OR turks OR turkmen* OR uganda* OR ukrain* OR uruguay* OR uzbek* OR vanuatu* OR new-hebride* OR venezuela* OR vietnam* OR viet-nam OR viet-names* OR middle-east* OR west-bank OR gaza OR palestin* OR yemen* OR yugoslav* OR zambia* OR zimbabwe* OR rhodesia* OR global-south OR magreb* OR maghrib* OR sahara* OR west-indies OR caribbean* OR central-america* OR latin-america* OR south-america* OR central-asia* OR north-asia* OR northern-asia* OR southeastern-asia* OR south-eastern-asia* OR southeast-asia* OR south-east-asia* OR west-asia* OR western-asia* OR east-europe* OR eastern-europe* OR lmic OR lmics OR third-world OR lami-countr* OR transitional-countr* OR ((developing OR less-developed OR lesser-developed OR under-developed OR underdeveloped OR middle-income OR low-income OR lower-income OR underserved OR under-served OR deprived OR poor OR poorer) NEAR/3 (countr* OR nation* OR population* OR world* OR econom*)) OR ((low OR lower) NEAR/3 (gdp OR gnp OR gross-domestic OR gross-national)) OR ((emerging) NEAR/3 (econom* OR nation*)))) OR (CU=(afghan* OR africa* OR albania* OR algeria* OR angola* OR argentin* OR armenia* OR azerbaijan* OR bangladesh* OR bangalee* OR bajan* OR belarus* OR byelarus* OR belorus* OR byelorus* OR belize* OR benin* OR dahomey OR bhutan* OR bolivia* OR bosnia* OR herzegovin* OR botswana* OR batswana* OR bechuanaland* OR brazil* OR brasil* OR bulgaria* OR burkina* OR upper-volta OR burundi* OR urundi* OR cabo-verde* OR cape-verde* OR cambodia* OR kampuchea* OR khmer* OR cameroon* OR cameron* OR cameroun* OR ubangi* OR chad OR chadian* OR chile OR chilean* OR china OR chinese OR colombia* OR comoro* OR comorian* OR mayotte* OR congo OR congolese OR zaire* OR costa-rica* OR cote-d-ivoir* OR ivory-coast* OR ivorian* OR croatia* OR cuba* OR djibouti* OR dominica* OR ecuador* OR egypt* OR united-arab-republic* OR salvador* OR equatoguinean* OR eritrea* OR estonia* OR eswatin* OR swazi* OR swati* OR ethiopia* OR fiji* OR gabon* OR gambia* OR georgia* OR ghana* OR gold-coast* OR gibraltar* OR grenad* OR guam* OR guatemala* OR guinea* OR guyan* OR guiana* OR haiti* OR hispaniola OR hondura* OR hungar* OR india* OR indonesia* OR timor* OR iran* OR iraq* OR jamaica* OR jordan* OR kazakh* OR kenya* OR north-korea* OR kosov* OR kyrgyz* OR kirghiz* OR kirgiz* OR kirghiz* OR lao OR laos OR loatian* OR latvia* OR lebanon* OR lesoth* OR basutoland OR mosotho* OR basotho* OR liberia* OR libya* OR lithuania* OR macedonia* OR madagasca* OR malagasy* OR malawi* OR nyasaland OR malaysia* OR maldiv* OR mali OR malian OR malians OR micronesia* OR kiribati* OR marshall-island* OR marshallese OR nauru* OR mariana* OR palau* OR tuvalu* OR mauritania* OR mauritian* OR mauritius OR mexico OR mexican* OR moldov* OR mongol* OR montenegr* OR morocc* OR ifni OR mozambi* OR myanma* OR burma* OR burmese* OR namibia* OR nauruan* OR nepal* OR nicaragua* OR niger OR nigeria* OR nigerien* OR oman* OR muscat OR pakistan* OR panama* OR paraguay* OR peru OR peruvian* OR philippin* OR philipin* OR phillipin* OR phillippin* OR filipin* OR poland OR polish OR pole OR poles OR romania* OR russia* OR ussr OR soviet-union* OR union-of-soviet-socialist-republic* OR rwand* OR ruand* OR samoa* OR pacific-island* OR polynesia* OR sao-tome* OR senegal* OR serbia* OR seychell* OR sierra-leone* OR slovak* OR melanesia* OR solomon-island* OR norfolk-island* OR somali* OR sri-lanka* OR ceylon* OR saint-kitt* OR st-kitt* OR kittitian* OR saint-lucia* OR st-lucia* OR saint-vincent* OR st-vincent* OR vincentian* OR grenadin* OR sudan* OR surinam* OR syria* OR tadjik* OR tadzhik* OR tajik* OR tanzania* OR tanganyika* OR thailand* OR thai OR siam OR timor* OR togo OR togolese* OR tonga* OR trinidad* OR tobago* OR tunisia* OR turkey OR turk OR turks OR turkmen* OR uganda* OR ukrain* OR uruguay* OR uzbek* OR vanuatu* OR new-hebride* OR venezuela* OR vietnam* OR viet-nam OR viet-names* OR middle-east* OR west-bank OR gaza OR palestin* OR yemen* OR yugoslav* OR zambia* OR zimbabwe* OR rhodesia* OR global-south OR magreb* OR maghrib* OR sahara* OR west-indies OR caribbean* OR central-america* OR latin-america* OR south-america* OR central-asia* OR north-asia* OR northern-asia* OR southeastern-asia* OR south-eastern-asia* OR southeast-asia* OR south-east-asia* OR west-asia* OR western-asia* OR east-europe* OR eastern-europe*))) AND (PY=(2005-2021)) NOT (TS=((animal* OR nonhuman* OR rat OR rats OR mouse OR mice OR rodent* OR murine* OR primate* OR monkey* OR dog OR dogs OR canine* OR pig* OR porcine*) NOT (human* AND (animal* OR nonhuman* OR rat OR rats OR mouse OR mice OR rodent* OR murine* OR primate* OR monkey* OR dog OR dogs OR canine* OR pig* OR porcine*)))) NOT (TI=(editorial* OR comment* OR case-report* OR systematic-review* OR meta-analys* OR ((trial*) AND (protocol*))))

*** * ***

**Global Index Medicus (World Health Organization): 3,798 results**

(tw:((hepatoblastoma* OR hepatocell* OR hcc OR cholangiocarcinoma* OR hepatocholangiocarcinoma* OR chcc* OR ((liver* OR hepatic* OR biliary OR hepatobiliary OR hepatopancrea* OR pancreatobiliary OR bile-canalicul* OR gallbladder* OR cholangiocellular* OR klatskin* OR "bile duct" OR “bile ducts”) AND (neoplas* OR cancer* OR adenoma* OR carcinoma* OR adenocarcinoma* OR malignan* OR tumor* OR tumour* OR metasta* OR lesion* OR mass*))) AND (hepatectom* OR posthepatectom* OR post-hepatectom* OR hepathectom* OR hepatopancreatoduodenectom* OR metastasectom* OR postmetastasectom* OR post-metastasectom* OR cholecystectom* OR lobectom* OR postlobectom* OR post-lobectom* OR surger* OR surgical* OR resection* OR debulk*))) AND NOT (mh:(Animals AND NOT (Humans AND Animals))) AND NOT (mh:(Editorial OR Comment OR clinical-trial-protocol)) AND NOT (ti:(case-report*OR systematic-review* OR meta-analys*))

**Section 3: Study and Participant Characteristics**

**Supplementary Table A**: Study Characteristics

| **Study ID** | **First Author's Last Name** | **Year of Publication** | **Study Title** | **Country** | **Income Status of Country** | **Study Design** | **Study Period** | **Sample Size** | **Males (%)** | **Indication For Surgery** |
| --- | --- | --- | --- | --- | --- | --- | --- | --- | --- | --- |
| 1 | Abd El-Kader et al. | 2018 | Percutaneous radiofrequency ablation compared with surgical resection in the treatment of early hepatocellular carcinoma | Egypt | lowerMIC | Quasi-Experimental | 2013 - 2017 | 40 | 82.50 | HCC |
| 2 | Abdel Wahab et al. | 2015 | Postoperative Outcome after Major Liver Resection in Jaundiced Patients with Proximal Bile Duct Cancer without Preoperative Biliary Drainage | Egypt | lowerMIC | Case-Control | 2000 - 2014 | 175 | 58.86 | HCC, CCA (perihilar and intrahepatic). CRC, GBC, GIST |
| 3 | Abdel-Wahab et al. | 2010 | Prognostic factors affecting survival and recurrence after hepatic resection for hepatocellular carcinoma in cirrhotic liver | Egypt | lowerMIC | Retrospective Cohort | 1995 - 2007 | 175 | 74.90 | HCC |
| 4 | Abdelraouf et al. | 2014 | Initial experience of surgical microwave tissue precoagulation in liver resection for hepatocellular carcinoma in cirrhotic liver | Egypt | lowerMIC | Case Series | - | 26 | 73.08 | HCC |
| 5 | Abdelwahab et al. | 2014 | Hilar cholangiocarcinoma in cirrhotic liver: a case-control study | Egypt | lowerMIC | Case-Control | 1995 - 2010 | 243 | 61.31 | Hilar CCA |
| 6 | Abreu et al. | 2020 | Liver resections for metastasis: surgical outcomes of a single center academic institution | Brazil | upperMIC | Retrospective Cohort | 2010 - 2015 | 86 | 44.20 | CRC, neuroendocrine tumours, or other primary cancers |
| 7 | Agarwal et al. | 2015 | Minimally invasive versus the conventional open surgical approach of a radical cholecystectomy for gallbladder cancer: a retrospective comparative study | India | lowerMIC | Retrospective Cohort | 2011 - 2013 | 70 | - | GBC |
| 8 | Ainthachot et al. | 2022 | Chromosomal aberrations, visualized using UroVysion fluorescence in-situ hybridization assay, can predict poor prognosis in formalin-fixed paraffin-embedded tissues of cholangiocarcinoma patients | Thailand | upperMIC | Retrospective Cohort | 2008 - 2017 | 194 | 64.95 | CCA |
| 9 | Akcam et al. | 2019 | Oncological Outcomes of Hepatic Resection vs Transplantation for Localized Hepatocellular Carcinoma | Turkiye | upperMIC | Retrospective Cohort | 2005 - 2017 | 38 | 92.11 | HCC |
| 10 | Aksoy et al. | 2020 | Comparison of Resection and Liver Transplant in Treatment of Hepatocellular Carcinoma | Turkiye | upperMIC | Retrospective Cohort | 1998 - 2010 | 36 | 66.67 | HCC |
| 11 | Alexandrescu et al. | 2012 | Simultaneous resection of the primary colorectal tumor and liver metastases - a safe and effective operation | Romania | upperMIC | Retrospective Cohort | 1995 - 2010 | 142 | 43.66 | CRC |
| 12 | Alexandrescu et al. | 2017 | Comparative Analysis between Simultaneous Resection and Staged Resection for Synchronous Colorectal Liver Metastases - A Single Center Experience on 300 Consecutive Patients | Romania | upperMIC | Retrospective Cohort | 1995 - 2016 | 300 | 55.67 | CRC |
| 13 | Alvarez et al. | 2015 | Associating liver partition and portal vein ligation for staged hepatectomy offers high oncological feasibility with adequate patient safety: a prospective study at a single center | Argentina | upperMIC | Prospective Cohort | 2011 - 2014 | 30 | 63.33 | HCC, CCA, CRC, Neuroendocrine Tumor, Breast CA, Esophageal CA, Leiomyosarcoma |
| 14 | Ardiles et al. | 2010 | Prognostic factors after resection of hepatocellular carcinoma in the non-cirrhotic liver: presentation of 51 cases | Argentina | upperMIC | Case Series | 1990 - 2006 | 51 | 64.71 | HCC |
| 15 | Aristizabal et al. | 2018 | Analysis of results after the implementation of fast recovery protocols in hepatopancreatobiliary surgery | Colombia | upperMIC | Retrospective Cohort | 2012 - 2017 | 199 | - | CCA, Metastatic liver cancer (unspecified primary) |
| 16 | Aysal et al. | 2022 | Tumoral and Parenchymal Morphological Assessment in Liver Metastases of Colorectal Carcinoma: Micrometastasis, Peritumoral Lymphocytes, Tumor Budding and Differentiation are Potential Prognostic Factors | Turkiye | upperMIC | Case Series | 2008 - 2016 | 100 | 47.00 | CRC |
| 17 | Bacalbasa et al. | 2015 | Liver resection for ovarian cancer liver metastases as part of cytoreductive surgery is safe and may bring survival benefit | Romania | upperMIC | Retrospective Cohort | 2002 - 2014 | 31 | - | Ovarian CA |
| 18 | Bacalbasa et al. | 2014 | Role of surgical treatment in breast cancer liver metastases: a single center experience | Romania | upperMIC | Case Series | 2002 - 2013 | 43 | - | Breast CA |
| 19 | Bari et al. | 2021 | Is it necessary to measure hepatic venous pressure gradient before liver resection in cirrhotic patients? A single center audit | Pakistan | lowerMIC | Case Series | 2015 - 2017 | 20 | 55.00 | HCC |
| 20 | Batra et al. | 2016 | Major Gastrointestinal Cancer Resections in the Elderly in India: Poised for Future Challenges | India | lowerMIC | Retrospective Cohort | 2006 - 2014 | 158 | - | Unspecified |
| 21 | Belev et al. | 2007 | Aggressive surgery in the multimodality treatment of liver metastases from colorectal cancer | Bulgaria | upperMIC | Case Series | 2004 - 2006 | 42 | - | CRC |
| 22 | Bhaijee et al. | 2011 | Liver resection for non-cirrhotic hepatocellular carcinoma in South African patients | South Africa | upperMIC | Case Series | 1990 - 2008 | 22 | 45.45 | HCC |
| 23 | Bhandare et al. | 2018 | Liver resection for HCC outside the BCLC criteria | India | lowerMIC | Retrospective Cohort | 2010 - 2015 | 100 | 85.00 | HCC |
| 24 | Bogdanovic et al. | 2020 | Impact of diseased liver parenchyma on perioperative outcome among patients with hepatocellular carcinoma undergoing hepatectomy: Experience from a developing country | Serbia | upperMIC | Retrospective Cohort | 2001 - 2017 | 190 | 60.53 | HCC |
| 25 | Bonadio et al. | 2019 | Conversion Chemotherapy With a Modified FLOX Regimen for Borderline or Unresectable Liver Metastases From Colorectal Cancer: An Alternative for Limited-Resources Settings | Brazil | upperMIC | Retrospective Cohort | 2009 - 2017 | 54 | 55.56 | CRC |
| 26 | Bredt et al. | 2014 | Predictors of recurrence after a first hepatectomy for colorectal cancer liver metastases: a retrospective analysis | Brazil | upperMIC | Retrospective Cohort | 2006 - 2010 | 101 | 63.37 | CRC |
| 27 | Burlaka et al. | 2020 | Colorectal cancer liver metastases within the central and peripheral segments: Parenchymal sparing surgery adaptation | Ukraine | lowerMIC | Prospective Cohort | 2015 - 2020 | 185 | - | CRC |
| 28 | Chaudhari et al. | 2018 | Outcome of neoadjuvant chemotherapy in "locally advanced/borderline resectable" gallbladder cancer: the need to define indications | India | lowerMIC | Retrospective Cohort | 2010 - 2016 | 66 | - | GBC |
| 29 | Chinburen et al. | 2015 | Impact of Glissonean pedicle approach for centrally located hepatocellular carcinoma in mongolia | Mongolia | lowerMIC | Retrospective Cohort | 2003 - 2012 | 69 | 50.72 | HCC |
| 30 | Chotirosniramit et al. | 2020 | The benefit of curative liver resection with a selective bile duct preserving approach for hepatocellular carcinoma with macroscopic bile duct tumor thrombus | Thailand | upperMIC | Retrospective Cohort | 2001 - 2016 | 168 | 75.00 | HCC (With and without macroscopic bile duct tumor thrombus) |
| 31 | Civil et al. | 2020 | Long-Term Results and Prognostic Significance of Non-ANATOMIC Liver Resection for Colorectal Liver Metastasis: Single Center Experience | Turkiye | upperMIC | Retrospective Cohort | 2007 - 2011 | 44 | 65.91 | CRC |
| 32 | Cokmert et al. | 2014 | Survival outcomes of liver metastasectomy in colorectal cancer cases: a single-center analysis in Turkey | Turkiye | upperMIC | Retrospective Cohort | 1996 - 2013 | 99 | 64.65 | CRC |
| 33 | Costa et al. | 2022 | Repeat hepatectomy for recurrent colorectal liver metastases: A comparative analysis of short- and long-term results | Brazil | upperMIC | Retrospective Cohort | 2000 - 2020 | 709 | 46.69 | CRC |
| 34 | Daradkeh et al. | 2021 | A case series of hilar cholangiocarcinoma: A single surgeon experience over 20-years | Jordan | upperMIC | Case Series | 1996 - 2016 | 22 | 63.64 | Hilar CCA |
| 35 | deSantibanes et al. | 2015 | Associated liver and multivisceral resections: should we extend the frontiers of resectability? | Argentina | upperMIC | Case Series | 2007 - 2013 | 21 | 47.62 | CRC, neuroendocrine tumors, retroperitoneal liposarcoma, pancreatic insulinoma |
| 36 | deSantibanes et al. | 2010 | Short-term and long-term outcomes after simultaneous resection of colorectal malignancies and synchronous liver metastases | Argentina | upperMIC | Retrospective Cohort | 1982 - 2006 | 185 | 61.62 | CRC |
| 37 | Diaconescu et al. | 2017 | Resection of Concomitant Hepatic and Extrahepatic Metastases from Colorectal Cancer - A Worthwhile Operation? | Romania | upperMIC | Retrospective Cohort | 1996 - 2016 | 678 | - | CRC |
| 38 | Dulundu et al. | 2017 | Simultaneous resection for colorectal cancer with synchronous liver metastases is a safe procedure: Outcomes at a single center in Turkey | Turkiye | upperMIC | Retrospective Cohort | 2005 - 2016 | 108 | 53.70 | CRC |
| 39 | Dumitrascu et al. | 2016 | Major hepatectomies for perihilar cholangiocarcinoma: Predictors for clinically relevant postoperative complications using the International Study Group of Liver Surgery definitions | Romania | upperMIC | Retrospective Cohort | 1996 - 2012 | 70 | 57.14 | Perihilar CCA |
| 40 | Dumitrascu et al. | 2017 | Curative-intent Surgery for Perihilar Cholangiocarcinoma with and without Portal Vein Resection - A Comparative Analysis of Early and Late Outcomes | Romania | upperMIC | Retrospective Cohort | 1996 - 2014 | 123 | 56.91 | Perihilar CCA |
| 41 | Efanov et al. | 2020 | Combining E-PASS model and disease specific risk factors to predict severe morbidity after liver and bile duct resection for perihilar cholangiocarcinoma | Russia | upperMIC | Retrospective Cohort | 2013 - 2019 | 119 | 48.74 | Perihilar CCA |
| 42 | El-Gendi et al. | 2013 | Intraoperative ablation for small HCC not amenable for percutaneous radiofrequency ablation in Child A cirrhotic patients | Egypt | lowerMIC | Retrospective Cohort | 2009 - 2012 | 27 | 48.10 | HCC |
| 43 | El-Gendi et al. | 2018 | Combined liver resection and transarterial chemoembolization versus liver resection alone for the management of solitary large exophytic hepatocellular carcinoma with extrahepatic arterial supply: is two always better than one? | Egypt | lowerMIC | Randomised Controlled Trial | 2015 - 2017 | 108 | 42.59 | HCC |
| 44 | El-Gendi et al. | 2018 | Laparoscopic Versus Open Hepatic Resection for Solitary Hepatocellular Carcinoma Less Than 5cm in Cirrhotic Patients: A Randomized Controlled Study | Egypt | lowerMIC | Randomised Controlled Trial | - | 50 | 60.00 | HCC |
| 45 | Elsanousi et al. | 2018 | Operative outcome of liver resections for hepatocellular carcinoma: Retrospective case control study of a twelve-years pioneer experience in the Sudan | Sudan | LIC | Case-Control | 2002 - 2013 | 44 | 72.73 | HCC |
| 46 | Elshaarawy et al. | 2021 | Outcomes of curative liver resection for hepatocellular carcinoma in patients with cirrhosis | Egypt | lowerMIC | Retrospective Cohort | 2010 - 2017 | 120 | - | HCC |
| 47 | Fadel et al. | 2020 | Laparoscopic liver resection for hepatocellular carcinoma: a single-center experience in upper egypt | Egypt | lowerMIC | Case Series | 2017 - 2019 | 20 | 60.00 | HCC |
| 48 | Fontana et al. | 2014 | Surgical outcomes and prognostic factors in patients with synchronous colorectal liver metastases | Brazil | upperMIC | Case Series | 1996 - 2007 | 59 | 49.15 | CRC |
| 49 | Galun et al. | 2012 | Is there any benefit from expanding the criteria for the resection of hepatocellular carcinoma in cirrhotic liver? Experience from a developing country | Serbia | upperMIC | Retrospective Cohort | 2001 - 2008 | 40 | 62.50 | HCC |
| 50 | Galun et al. | 2018 | Preoperative neutrophil-to-lymphocyte ratio as a prognostic predictor after curative-intent surgery for hepatocellular carcinoma: experience from a developing country | Serbia | upperMIC | Retrospective Cohort | 2001 - 2012 | 109 | 59.60 | HCC |
| 51 | Galun et al. | 2021 | Short- and Long-Term Outcomes After Hepatectomy in Elderly Patients with Hepatocellular Carcinoma: An Analysis of 229 Cases from a Developing Country | Serbia | upperMIC | Retrospective Cohort | 2009 - 2018 | 229 | 58.95 | HCC |
| 52 | Goel et al. | 2022 | Node positivity in T1b gallbladder cancer: A high volume centre experience | India | lowerMIC | Retrospective Cohort | 2010 - 2021 | 76 | 26.32 | GBC |
| 53 | Govil et al. | 2016 | Liver resection for perihilar cholangiocarcinoma - why left is sometimes right | India | lowerMIC | Retrospective Cohort | 2009 - 2015 | 36 | 72.22 | Perihilar CCA |
| 54 | Goyal et al. | 2021 | Prognostic significance of tumour budding, tumour-stroma ratio and desmoplastic stromal reaction in gall bladder carcinoma | India | lowerMIC | Retrospective Cohort | 2010 - 2020 | 96 | 16.67 | GBC |
| 55 | Grigorie et al. | 2017 | Curative Intent Treatment of Hepatocellular Carcinoma - 844 Cases Treated in a General Surgery and Liver Transplantation Center | Romania | upperMIC | Retrospective Cohort | 2001 - 2016 | 518 | 70.85 | HCC |
| 56 | Gupta et al. | 2022 | Radical surgery for de novo gallbladder carcinoma-Single-center analysis of prognostic factors and survival outcomes from an endemic region | India | lowerMIC | Retrospective Cohort | 2014 - 2018 | 115 | 20.00 | GBC |
| 57 | Hegazy et al. | 2019 | Liver stiffness measurement by transient elastography can predict outcome after hepatic resection for hepatitis C virus-induced hepatocellular carcinoma | Egypt | lowerMIC | Retrospective Cohort | 2015 - 2017 | 40 | 77.50 | HCC |
| 58 | Herman et al. | 2016 | IS RESECTION OF HEPATOCELLULAR CARCINOMA IN THE ERA OF LIVER TRANSPLANTATION WORTHWILE? A single center experience | Brazil | upperMIC | Case Series | 2000 - 2014 | 101 | 65.35 | HCC |
| 59 | Herman et al. | 2014 | Laparoscopic resection of hepatocellular carcinoma: when, why, and how? A single-center experience | Brazil | upperMIC | Case Series | 2007 - 2013 | 30 | 70.00 | HCC |
| 60 | Ho et al. | 2021 | Hepatectomy with Takasaki's Technique Using SonaStar Ultrasonic Aspiration System: An Experience from 58 Cases | Vietnam | lowerMIC | Case Series | 2018 - 2021 | 58 | 86.21 | HCC |
| 61 | Iancu et al. | 2008 | Survival prognostic factors in patients with resection of liver metastasis from colorectal cancer | Romania | upperMIC | Retrospective Cohort | 2002 - 2005 | 63 | 52.38 | CRC |
| 62 | Ibraheem et al. | 2022 | Extra-hepatic Glissonean approach allows safe segmental liver resection in patients with cirrhosis with hepatocellular carcinoma | Egypt | lowerMIC | Retrospective Cohort | 2014 - 2016 | 87 | 58.62 | HCC |
| 63 | Jayme et al. | 2021 | Infiltrative Tumor Borders in Colorectal Liver Metastasis: Should We Enlarge Margin Size? | Brazil | upperMIC | Retrospective Cohort | 2004 - 2019 | 266 | 55.26 | CRC |
| 64 | Joshi et al. | 2021 | Impact of enhanced recovery pathway in 408 gallbladder cancer resections | India | lowerMIC | Prospective Cohort | 2014 - 2019 | 408 | 30.15 | GBC |
| 65 | Kalayarasan et al. | 2013 | Squamous variant of gallbladder cancer: is it different from adenocarcinoma? | India | lowerMIC | Retrospective Cohort | 2009 - 2012 | 136 | 22.06 | GBC |
| 66 | Kavlakoglu et al. | 2011 | Surgical treatment of liver metastases from colorectal cancer: experience of a single institution | Turkiye | upperMIC | Retrospective Cohort | 2004 - 2007 | 42 | 52.38 | CRC |
| 67 | Khalil et al. | 2018 | Resection of hepatocellular carcinoma in cirrhotic patients: laparoscopic versus open resection | Egypt | lowerMIC | Randomised Controlled Trial | 2014 - 2016 | 65 | 44.62 | HCC |
| 68 | Khuntikeo et al. | 2008 | Major hepatic resection for hilar cholangiocarcinoma without preoperative biliary drainage | Thailand | upperMIC | Retrospective Cohort | 1999 - 2002 | 30 | 70.00 | CCA |
| 69 | Kostov et al. | 2013 | Prognostic factors related to surgical outcome of liver metastases of breast cancer | Bulgaria | upperMIC | Retrospective Cohort | 2001 - 2007 | 42 | 0.00 | Breast CA |
| 70 | Kostov et al. | 2009 | Segmental liver resection for colorectal metastases | Bulgaria | upperMIC | Retrospective Cohort | 2000 - 2007 | 188 | 60.63 | CRC |
| 71 | Kruger et al. | 2018 | Evolution in the surgical management of colorectal liver metastases: Propensity score matching analysis (PSM) on the impact of specialized multidisciplinary care across two institutional eras | Brazil | upperMIC | Retrospective Cohort | 2000 - 2014 | 176 |  | CRC |
| 72 | Kumar et al. | 2019 | Multimodality management of incidentally detected gall bladder cancer: long term results from a tertiary care cancer centre | India | lowerMIC | Retrospective Cohort | 2002 - 2012 | 34 | - | GBC |
| 73 | Leeratanakachorn et al. | 2021 | Infrahepatic Inferior Vena Cava Clamping Reduces Blood Loss during Liver Transection for Cholangiocarcinoma | Thailand | upperMIC | Retrospective Cohort | 2015 - 2016 | 116 | 62.07 | CCA |
| 74 | Long et al. | 2013 | Laparoscopic liver resection: 5-year experience at a single center | Vietnam | lowerMIC | Retrospective Cohort | 2008 - 2012 | 173 | 75.14 | HCC |
| 75 | Lopes et al. | 2016 | Influence of Hepatocellular Carcinoma Etiology in the Survival after Resection | Brazil | upperMIC | Retrospective Cohort | 2000 - 2014 | 101 | - | HCC |
| 76 | Luna-Abanto et al. | 2020 | Liver Resection as Part of Cytoreductive Surgery for Ovarian Cancer | Peru | upperMIC | Retrospective Cohort | 2009 - 2017 | 39 | 0.00 | Ovarian CA |
| 77 | Machado et al. | 2017 | Extended laparoscopic liver resection: initial experience and review of the literature | Brazil | upperMIC | Case Series | 2007 - 2016 | 23 | 43.48 | CCA, CRC, hemangioendothelioma, angiomyolipoma |
| 78 | Makdissi et al. | 2021 | A Combined "Hanging Liver Maneuver" and "Intrahepatic Extra-Glissonian Approach" for Anatomical Right Hepatectomy: Technique Standardization, Results, and Correlation With Portal Pedicle Anatomy | Brazil | upperMIC | Case Series | 2014 - 2020 | 30 | 73.33 | CRC |
| 79 | Makhlouf et al. | 2020 | Risk of liver failure after major hepatectomy for patients with hepatocellular carcinoma | Egypt | lowerMIC | Case-Control | 2013 - 2017 | 28 | 67.86 | HCC |
| 80 | Mannai et al. | 2010 | The model of end-stage liver disease (MELD) score in predicting postoperative liver failure after hepatic resection of hepatocellular carcinoma in cirrhotic patients: The Tunisian experience | Tunisia | lowerMIC | Retrospective Cohort | 1991 - 2007 | 26 | 50.00 | HCC |
| 81 | Marques et al. | 2018 | Is primary sidedness a prognostic factor in patients with resected colon cancer liver metastases (CLM)? | Brazil | upperMIC | Retrospective Cohort | 1998 - 2012 | 151 | 49.67 | CRC |
| 82 | Maurette et al. | 2017 | Laparoscopic liver resection in metastatic colorectal cancer treatment: comparison with long-term results using the conventional approach | Argentina | upperMIC | Retrospective Cohort | 2007 - 2015 | 40 | 65.00 | CRC |
| 83 | Meira Junior et al. | 2022 | Platelet-albumin (PAL) score as a predictor of perioperative outcomes and survival in patients with hepatocellular carcinoma undergoing liver resection in a Western center | Brazil | upperMIC | Retrospective Cohort | 2008 - 2019 | 182 | 67.58 | HCC |
| 84 | Mogahed et al. | 2021 | The value of intra-operative ultrasonography on safety margin and outcome during liver resection and radio-frequency ablation in the management of hepatocellular carcinoma patients | Egypt | lowerMIC | Prospective and Retrospective Cohort | 2017 - 2020 | 76 | 76.32 | HCC |
| 85 | Molek et al. | 2021 | Factors influencing the five-year recurrence of liver carcinoma after surgery | Thailand | upperMIC | Retrospective Cohort | 2010 - 2014 | 76 | 61.84 | HCC, CCA |
| 86 | Nag et al. | 2021 | Bi-segmentectomy versus wedge hepatic resection in extended cholecystectomy for T2 and T3 gallbladder cancer: A matched case-control study | India | lowerMIC | Case-Control | 2009 - 2019 | 102 | 31.37 | GBC |
| 87 | Nari et al. | 2018 | Short and long-term outcomes of the re-hepatectomies as part of multi-modal treatment of hepatic metastases from colo-rectal origin. Bi-institutional study | Argentina | upperMIC | Retrospective Cohort | 1997 - 2013 | 444 | 68.55 | CRC |
| 88 | Negi et al. | 2011 | Lymph nodal involvement as prognostic factor in gallbladder cancer: location, count or ratio? | India | lowerMIC | Retrospective Cohort | 2003 - 2009 | 57 | 22.81 | GBC |
| 89 | Nicolas et al. | 2022 | Laparoscopic vs open liver resection for metastatic colorectal cancer: analysis of surgical margin status and survival | Argentina | upperMIC | Retrospective Cohort | 2007 - 2017 | 82 | 46.34 | CRC |
| 90 | Ninh et al. | 2021 | The Application of Selective Hepatic Inflow Vascular Occlusion with Anterior Approach in Liver Resection: Effectiveness in Managing Major Complications and Long-Term Survival | Vietnam | lowerMIC | Case Series | 2011 - 2014 | 72 | 83.33 | HCC, CCA, CRC |
| 91 | Pandey et al. | 2018 | Surgico-pathological Outcomes of 148 Radical Cholecystectomies Using Systematic Regional Lymphadenectomy Protocol: a Retrospective Study | India | lowerMIC | Case Series | 2007 - 2017 | 148 | 64.03 | GBC |
| 92 | Panwar et al. | 2016 | Hepatic resection for predominantly large size hepatocellular carcinoma: Early and long-term results from a tertiary care center in India | India | lowerMIC | Retrospective Cohort | 1987 - 2013 | 81 | 86.40 | HCC |
| 93 | Parau et al. | 2015 | Determinants of survival after liver resection for metastatic colorectal carcinoma | Romania | upperMIC | Retrospective Cohort | 2006 - 2011 | 70 | 52.86 | CRC |
| 94 | Patkar et al. | 2020 | Towards Standardization of Liver Resections in India: Five Hundred Consecutive Oncological Liver Resections- Trends, Techniques and Outcomes | India | lowerMIC | Case Series | 2010 - 2016 | 516 | 61.54 | HCC, CCA, CRC, neuroendocrine liver metastases, non-neuroendocrine or CRC liver metastases, others (unspecified) |
| 95 | Patkar et al. | 2019 | Achieving margin negative resection-doing less is justified: oncological outcomes of wedge excision of liver in gallbladder cancer (GBC) surgery | India | lowerMIC | Case Series | 2010 - 2015 | 97 | - | GBC |
| 96 | Patkar et al. | 2018 | Emerging role of multimodality treatment in gall bladder cancer: Outcomes following 510 consecutive resections in a tertiary referral center | India | lowerMIC | Retrospective Cohort | 2010 - 2015 | 313 |  | GBC |
| 97 | Patkar et al. | 2021 | Is resection for noncolorectal, nonneuroendocrine liver metastases justified? | India | lowerMIC | Case Series | 2010 - 2016 | 50 | 48.00 | Non-CRC, non-neuroendocrine metastases |
| 98 | Petrovic et al. | 2010 | The results of surgical treatment of metastatic liver tumor | Serbia | upperMIC | Case Series | 2003 - 2007 | 33 | 51.52 | CRC, Breast CA, Gastric CA, Ovarian CA, PDAC |
| 99 | Popescu et al. | 2005 | Current treatment of hepatocellular carcinoma. Analysis of a series of 123 cases over a 5-year period | Romania | upperMIC | Retrospective Cohort | 2000 - 2004 | 84 | 75.00 | HCC |
| 100 | Popescu et al. | 2012 | Metastatic colorectal cancer--what about the primary? | Romania | upperMIC | Prospective and Retrospective Cohort | 1995 - 2012 | 209 | - | CRC |
| 101 | Pottakkat et al. | 2013 | Evaluation of a prospective surgical strategy of extended resection to achieve R0 status in gall bladder cancer | India | lowerMIC | Case Series | 2008 - 2011 | 40 | 27.00 | GBC |
| 102 | Quesada-Soto et al. | 2017 | Liver Metastasectomy and Systemic Therapy Improve Overall Survival Compared With Surgery Alone After Curative Liver Resection of Colorectal Metastases in a Developing Country (Costa Rica) | Costa Rica | upperMIC | Retrospective Cohort | 2009 - 2014 | 51 | 76.47 | CRC |
| 103 | Qureshi et al. | 2020 | Feasibility of Nonanatomical Liver Resection in Diligently Selected Patients with Hepatoblastoma and Comparison of Outcomes with Anatomic Resection | India | lowerMIC | Retrospective Cohort | 2008 - 2019 | 120 | 68.33 | Hepatoblastoma |
| 104 | Rachdi et al. | 2019 | Local treatment of liver and lung metastases from colorectal cancer: a multicenter Tunisian study | Tunisia | lowerMIC | Retrospective Cohort | 2003 - 2015 | 61 | - | CRC |
| 105 | Rammohan et al. | 2015 | Bile duct thrombi in hepatocellular carcinoma: is aggressive surgery worthwhile? | India | lowerMIC | Retrospective Cohort | 1997 - 2012 | 426 | 71.83 | HCC |
| 106 | Ribeiro et al. | 2012 | Prognostic factors for survival in patients with colorectal liver metastases: experience of a single brazilian cancer center | Brazil | upperMIC | Retrospective Cohort | 1998 - 2009 | 170 | 53.53 | CRC |
| 107 | Ribeiro et al. | 2013 | Extended preoperative chemotherapy, extent of liver resection and blood transfusion are predictive factors of liver failure following resection of colorectal liver metastasis | Brazil | upperMIC | Retrospective Cohort | 1998 - 2009 | 170 | 53.53 | CRC |
| 108 | Ruiz et al. | 2022 | A preoperative nomogram for predicting long-term survival after resection of large hepatocellular carcinoma (>10 cm) | Peru | upperMIC | Retrospective Cohort | 1990 - 2015 | 234 | 40.60 | HCC |
| 109 | Ruiz et al. | 2016 | Hepatocellular carcinoma surgery outcomes in the developing world: A 20-year retrospective cohort study at the National Cancer Institute of Peru | Peru | upperMIC | Retrospective Cohort | 1991 - 2011 | 253 | 59.30 | HCC |
| 110 | Rungsakulkij et al. | 2018 | Prognostic factors in patients with HBV-related hepatocellular carcinoma following hepatic resection | Thailand | upperMIC | Retrospective Cohort | 2006 - 2015 | 217 | 46.08 | HCC |
| 111 | Saber et al. | 2020 | Minimally invasive vs traditional liver resection in managing small hepatocellular carcinoma | Egypt | lowerMIC | Randomised Controlled Trial | 2018 - 2019 | 30 | 36.67 | HCC |
| 112 | Saglam et al. | 2022 | Results of Intrahepatic Cholangiocarcinoma Resections: a Single-Center Analysis | Turkiye | upperMIC | Retrospective Cohort | 2013 - 2020 | 31 | 51.61 | CCA |
| 113 | Said et al. | 2021 | Predictive factors for long-term survival after hepatic resection for hepatocellular carcinoma: a single-center experience | Egypt | lowerMIC | Retrospective Cohort | 2010 - 2018 | 230 | 79.13 | HCC |
| 114 | Sapmaz et al. | 2020 | Incidental Gallbladder Cancer Diagnosed During Or After Laparoscopic Cholecystectomy, What Did We Do? | Turkiye | upperMIC | Case Series | 2015 - 2020 | 20 | 20.00 | GBC |
| 115 | Saritas et al. | 2020 | Comparison of surgical and percutaneous ablation methods in hepatic metastases from colorectal cancer | Turkiye | upperMIC | Retrospective Cohort | 2008 - 2019 | 86 | 67.44 | CRC |
| 116 | Sawangkajohn et al. | 2020 | Re-Rising of Total Bilirubin Level after Postoperative Day 3 (The V Pattern) Predicting Liver Failure and Survival of Patients who Underwent Hepatectomy for Cholangiocarcinoma | Thailand | upperMIC | Retrospective Cohort | 2015 - 2016 | 116 | 62.07 | CCA |
| 117 | Senbel et al. | 2017 | Role of Hepatic Resection for HCC in the era of Transplantation; an Experience of Two Tertiary Egyptian Centers | Egypt | lowerMIC | Prospective and Retrospective Cohort | 2010 - 2015 | 84 | 75.00 | HCC |
| 118 | Shehta et al. | 2021 | Post-hepatectomy liver failure after hepatic resection for hepatocellular carcinoma: a single center experience | Egypt | lowerMIC | Retrospective Cohort | 2010 - 2019 | 268 | 79.90 | HCC |
| 119 | Shehta et al. | 2021 | Outcomes of Hepatic Resection for Hepatocellular Carcinoma Associated with Portal Vein Invasion | Egypt | lowerMIC | Retrospective Cohort (with Propensity Matching) | 2009 - 2019 | 288 | 80.60 | HCC |
| 120 | Shehta et al. | 2020 | Recurrence of hilar cholangiocarcinoma after surgical resection: is there a role of surgery? | Egypt | lowerMIC | Retrospective Cohort | 1995 - 2010 | 263 | 62.40 | CCA |
| 121 | Soliman et al. | 2017 | Laparoscopic left lateral bisegmentectomy for hepatocellular carcinoma: moving from peripheral to anatomical | Egypt | lowerMIC | Retrospective Cohort | 2008 - 2016 | 38 | 81.48 | HCC |
| 122 | Sriputtha et al. | 2013 | Survival rate of intrahepatic cholangiocarcinoma patients after surgical treatment in Thailand | Thailand | upperMIC | Retrospective Cohort | 2005-2009 | 73 | 58.90 | CCA |
| 123 | Taesombat et al. | 2020 | Benefits of simultaneous laparoscopic colorectal surgery and liver resection for colorectal cancer with synchronous liver metastases: Retrospective case-matched study | Thailand | upperMIC | Retrospective Cohort (with Propensity Matching) | 2010 - 2019 | 36 | 52.78 | CRC |
| 124 | Taesombat et al. | 2020 | Long-term outcomes of laparoscopic versus open liver resection for hepatocellular  carcinoma: Retrospective case-matched study | Thailand | upperMIC | Retrospective Cohort (with Propensity Matching) | 2007 - 2013 | 54 | - | HCC |
| 125 | Takorov et al. | 2016 | Laparoscopic combined colorectal and liver resections for primary colorectal cancer with synchronous liver metastases | Bulgaria | upperMIC | Retrospective Cohort | 2012 - 2015 | 27 | - | CRC |
| 126 | Techathuvanan et al. | 2015 | Comparison between disease free survival of hepatocellular carcinoma after hepatic resection in chronic hepatitis B patients with or without cirrhosis | Thailand | upperMIC | Retrospective Cohort | 2001 - 2011 | 215 | 72.09 | HCC |
| 127 | Tohra et al. | 2021 | Experience With Changing Etiology and Nontransplant Curative Treatment Modalities for Hepatocellular Carcinoma in a Real-Life Setting-A Retrospective Descriptive Analysis | India | lowerMIC | Retrospective Cohort | 2007 - 2015 | 38 | 94.74 | HCC |
| 128 | Tomas et al. | 2020 | Is It Rational to Perform Liver Resection for Patients with Intermediate and Advanced Stages of Hepatocellular Carcinoma? | Turkiye | upperMIC | Retrospective Cohort | 2007 - 2016 | 54 | 83.33 | HCC |
| 129 | Tongsiri et al. | 2020 | Comparison of early clinical outcomes between intermittent vascular inflow occlusion versus intermittent selective hepatic vascular exclusion in hepatic resections for cholangiocarcinoma patients: A prospective randomized controlled trial study | Thailand | upperMIC | Randomised Controlled Trial | 2018 - 2019 | 40 | 37.50 | CCA |
| 130 | Valadares et al. | 2015 | Resection of liver metastasis from neuroendocrine tumors: evaluation of results and prognostic factors | Brazil | upperMIC | Retrospective Cohort | 1997 - 2007 | 22 | 54.55 | Neuroendocrine tumours |
| 131 | VazdaSilva et al. | 2020 | Predictors of long-term survival in patients with hepatic resection of colorectal metastases: Analysis of a Brazilian Cancer Center Cohort | Brazil | upperMIC | Retrospective Cohort | 2000-2012 | 280 | 56.07 | CRC |
| 132 | Wahab et al. | 2014 | Predictors of recurrence in hepatitis C virus related hepatocellular carcinoma after hepatic resection: a retrospective cohort study | Egypt | lowerMIC | Retrospective Cohort | 2002 - 2011 | 208 | 75.48 | HCC |
| 133 | Wahab et al. | 2012 | Caudate lobe resection with major hepatectomy for central cholangiocarcinoma: is it of value? | Egypt | lowerMIC | Retrospective Cohort | 1995 - 2010 | 159 | 59.12 | CCA |
| 134 | Younes et al. | 2020 | Surgical Resection of Hepatoblastoma: Factors Affecting Local Recurrence | Egypt | lowerMIC | Retrospective Cohort | 2007 - 2019 | 133 | 56.39 | Hepatoblastoma |
| 135 | Zakaria et al. | 2020 | Alpha-fetoprotein level to total tumor volume as a predictor of hepatocellular carcinoma recurrence after resection. A retrospective cohort study | Egypt | lowerMIC | Retrospective Cohort | 2010 - 2018 | 286 | 79.37 | HCC |
| *upperMIC: Upper Middle Income Country; lowerMIC: Lower Middle Income Country; LIC: Low Income Country; HCC: Hepatocellular Carcinoma; GBC: Gallbladder Carcinoma; CCA: Cholangiocarcinoma; CRC: Colorectal Cancer; CA: Carcinoma; GIST: Gastrointestinal Stromal Tumour* | | | | | | | | | | |

**Supplementary Table B:** Participant Characteristics

| **Study ID** | **First Author's Last Name** | **Year of Publication** | **TNM Stage (Overall)** | | | | **ASA ≥3** | **Child Pugh Class** | | | **HBV** | **HCV** | **ECOG Performance Status** | | | | | **Chemotherapy** | | **Type of Surgery** | | | | | **Multivisceral Resection** | **Intra/Postoperative Ablation** |
| --- | --- | --- | --- | --- | --- | --- | --- | --- | --- | --- | --- | --- | --- | --- | --- | --- | --- | --- | --- | --- | --- | --- | --- | --- | --- | --- |
|  |  |  | **I** | **II** | **III** | **IV** |  | **A** | **B** | **C** |  |  | **0** | **1** | **2** | **3** | **4** | **Neoadjuvant** | **Adjuvant** | **Open** | **Laparoscopic** | **Robotic** | **Major** | **Minor** |  |  |
| 1 | Abd El-Kader et al. | 2018 | BCLC Staging System: A1: 21; A2: 10; A3: 9 | | | | - | 40 | 0 | 0 | 18 | 15 | 35 | 5 | 0 | 0 | 0 | - | - | 40 | - | - | - | - | - | - |
| 2 | Abdel Wahab et al. | 2015 | - | - | - | - | - | - | - | - | - | - | - | - | - | - | - | - | - | 175 | - | - | 175 | - | 5 | - |
| 3 | Abdel-Wahab et al. | 2010 | 119 | 19 | 37 | - | - | 152 | 23 | - | 12 | 145 | - | - | - | - | - | - | - | 175 | - | - | 65 | 110 | - | - |
| 4 | Abdelraouf et al. | 2014 | - | - | - | - | - | 16 | 10 | - | - | 26 | - | - | - | - | - | - | - | 26 | - | - | - | 26 | - | 26 |
| 5 | Abdelwahab et al. | 2014 | - | - | - | - | - | - | - | - | 4 | 101 | - | - | - | - | - | - | - | 243 | - | - | 173 | 70 | - | - |
| 6 | Abreu et al. | 2020 | - | - | - | - | 12 | - | - | - | - | - | 17 | 58 | 11 | - | - | - | - | 82 | 4 | - | 68 | 18 | - | - |
| 7 | Agarwal et al. | 2015 | 8 | 20 | 42 | - | - | - | - | - | - | - | - | - | - | - | - | - | - | 46 | 24 | - | - | 70 | - | - |
| 8 | Ainthachot et al. | 2022 | - | - | - | - | - | - | - | - | - | - | - | - | - | - | - | - | - | - | - | - | - | - | - | - |
| 9 | Akcam et al. | 2019 | - | - | - | - | - | 30 | 6 | 2 | 24 | 3 | - | - | - | - | - | - | - | - | - | - | - | - | - | - |
| 10 | Aksoy et al. | 2020 | - | - | - | - | - | 26 | 10 | 0 | 23 | 2 | - | - | - | - | - | - | - | - | - | - | - | - | - | - |
| 11 | Alexandrescu et al. | 2012 | - | - | - | - | - | - | - | - | - | - | - | - | - | - | - | - | - | - | - | - | 30 | 112 | 117 | - |
| 12 | Alexandrescu et al. | 2017 | - | - | - | - | - | - | - | - | - | - | - | - | - | - | - | 82 | - | - | - | - | 23 | 94 | 234 | - |
| 13 | Alvarez et al. | 2015 | - | - | - | - | 8 | - | - | - | - | - | - | - | - | - | - | 18 | - | - | - | - | 30 | - | 11 | - |
| 14 | Ardiles et al. | 2010 | 20 | 8 | 21 | 1 | - | - | - | - | 5 | 3 | - | - | - | - | - | - | - | - | - | - | 38 | 13 | 10 | 1 |
| 15 | Aristizabal et al. | 2018 | - | - | - | - | - | - | - | - | - | - | - | - | - | - | - | - | - | - | - | - | - | - | - | - |
| 16 | Aysal et al. | 2022 | - | - | - | - | - | - | - | - | - | - | - | - | - | - | - | 49 | - | - | - | - | 23 | 77 | - | - |
| 17 | Bacalbasa et al. | 2015 | - | - | - | - | - | - | - | - | - | - | - | - | - | - | - | 2 | 15 | - | - | - | 2 | 21 | - | 1 |
| 18 | Bacalbasa et al. | 2014 | - | - | - | - | - | - | - | - | - | - | - | - | - | - | - | 35 | 41 | - | - | - | 29 | 14 | - | - |
| 19 | Bari et al. | 2021 | - | - | - | - | 16 | 20 | - | - | 1 | 19 | - | - | - | - | - | - | - |  | - | - | - | - | - | - |
| 20 | Batra et al. | 2016 | - | - | - | - | - | - | - | - | - | - | - | - | - | - | - | - | - |  | - | - | 87 | 71 | - | - |
| 21 | Belev et al. | 2007 | - | - | - | - | - | - | - | - | - | - | - | - | - | - | - | 8 | - | - | - | - | 26 | 16 | - | 4 |
| 22 | Bhaijee et al. | 2011 | - | - | - | - | - | 0 | 0 | 0 | 0 | 0 | - | - | - | - | - | - | - | 22 | - | - | 17 | 5 | 6 | - |
| 23 | Bhandare et al. | 2018 | BCLC Staging System: A: 25; B: 64; C: 11 | | | | - | 93 | 7 | - | 27 | 6 | - | - | - | - | - | - | - |  | - | - | 60 | 40 | - | - |
| 24 | Bogdanovic et al. | 2020 | - | - | - | - | 73 | - | - | - | - | - | - | - | - | - | - | - | - | 190 | - | - | 43 | 147 | - | - |
| 25 | Bonadio et al. | 2019 | - | - | - | - | - | - | - | - | - | - | 48 | | 4 | - | - | 54 | - | - | - | - | - | - | - | 6 |
| 26 | Bredt et al. | 2014 | - | - | - | - | - | - | - | - | - | - | - | - | - | - | - | 78 | 73 | - | - | - | - | - | 14 | 8 |
| 27 | Burlaka et al. | 2020 | - | - | - | - | - | - | - | - | - | - | - | - | - | - | - | - | - | 35 | 150 | - | 19 | 167 | 33 | - |
| 28 | Chaudhari et al. | 2018 | - | - | - | - | - | - | - | - | - | - | - | - | - | - | - | - | 48 |  | - | - | - | - | 3 | - |
| 29 | Chinburen et al. | 2015 | 0 | 11 | 48 | 10 | - | 44 | 1 | 0 | 22 | 28 | - | - | - | - | - | - | - |  | - | - | - | - | - | - |
| 30 | Chotirosniramit et al. | 2020 | - | - | - | - | - | - | - | - | - | - | - | - | - | - | - | - | - | - | - | - | - | - | - | - |
| 31 | Civil et al. | 2020 | - | - | - | - | 22 | - | - | - | - | - | - | - | - | - | - | - | - | 44 | - | - | 18 | 26 | - | 5 |
| 32 | Cokmert et al. | 2014 | - | - | - | - | - | - | - | - | - | - | - | - | - | - | - | - | 85 | - | - | - | - | - | - | - |
| 33 | Costa et al. | 2022 | - | - | - | - | 52 | - | - | - | - | - | - | - | - | - | - | - | - | 564 | 145 | - | 234 | 475 | 709 | 80 |
| 34 | Daradkeh et al. | 2021 | - | - | - | - | - | - | - | - | - | - | - | - | - | - | - | - | - | - | - | - | - | - | - | - |
| 35 | deSantibanes et al. | 2015 | - | - | - | - | 5 | - | - | - | - | - | - | - | - | - | - | - | - | - | - | - | 7 | - | 17 | - |
| 36 | deSantibanes et al. | 2010 | - | - | - | - | 0 | - | - | - | - | - | - | - | - | - | - | - | 185 | 185 | 0 | 0 | 42 | 143 | 185 | - |
| 37 | Diaconescu et al. | 2017 | - | - | - | - | - | - | - | - | - | - | - | - | - | - | - | - | - | - | - | - | - | - | 69 | - |
| 38 | Dulundu et al. | 2017 | - | - | - | - | 23 | - | - | - | - | - | - | - | - | - | - | 35 | - | - | - | - | 41 | 67 | 108 | - |
| 39 | Dumitrascu et al. | 2016 | - | - | - | - | - | - | - | - | - | - | - | - | - | - | - | - | - | - | - | - | 70 | - | - | - |
| 40 | Dumitrascu et al. | 2017 | - | - | - | - | - | - | - | - | - | - | - | - | - | - | - | - | 59 | - | - | - | - | - | - | - |
| 41 | Efanov et al. | 2020 | - | - | - | - | - | - | - | - | - | - | - | - | - | - | - | - | - | 119 | - | - | 119 | - | - | - |
| 42 | El-Gendi et al. | 2013 | - | - | - | - | - | - | - | - | - | - | - | - | - | - | - | - | - | 27 | - | - | - | 27 | - | - |
| 43 | El-Gendi et al. | 2018 | - | - | - | - | 19 | 108 | 0 | 0 | - | 99 | - | - | - | - | - | - | - |  | - | - | - | - | - | - |
| 44 | El-Gendi et al. | 2018 | - | - | - | - | 9 | - | - | - | - | - | - | - | - | - | - | - | - |  | - | - | - | - | - | - |
| 45 | Elsanousi et al. | 2018 | - | - | - | - | 0 | 36 | 8 | 0 | - | - | - | - | - | - | - | - | - | 44 | - | - | 8 | 36 | - | - |
| 46 | Elshaarawy et al. | 2021 | - | - | - | - | - | 120 | 0 | 0 | - | 105 | - | - | - | - | - | - | - | - | - | - | - | - | - | - |
| 47 | Fadel et al. | 2020 | - | - | - | - | - | 20 | 0 | 0 | - | - | - | - | - | - | - | - | - | 0 | 20 | - | - | 20 | - | - |
| 48 | Fontana et al. | 2014 | - | - | - | - | - | - | - | - | - | - | - | - | - | - | - | - | - | 59 | - | - | 33 | 26 | - | - |
| 49 | Galun et al. | 2012 | - | - | - | - | - | 29 | 11 | - | 19 | 10 | - | - | - | - | - | - | - | 40 | - | - | 22 | 18 | - | - |
| 50 | Galun et al. | 2018 | - | - | - | - | 47 | 83 | 26 | - | 30 | 23 | - | - | - | - | - | - | - | 109 | - | - | 39 | 70 | - | - |
| 51 | Galun et al. | 2021 | - | - | - | - | 134 | 118 | 11 | 0 | 61 | 88 | - | - | - | - | - | - | - | - | - | - | 28 | 201 | - | - |
| 52 | Goel et al. | 2022 | - | - | - | - | 3 | - | - | - | - | - | - | - | - | - | - | 9 | - | - | - | - | - | - | - | - |
| 53 | Govil et al. | 2016 | - | - | - | - | - | - | - | - | - | - | - | - | - | - | - | - | - | 36 | - | - | 36 | - | - | - |
| 54 | Goyal et al. | 2021 | 15 | 21 | 45 | 15 | - | - | - | - | - | - | - | - | - | - | - | - | - | - | - | - | - | - | - | - |
| 55 | Grigorie et al. | 2017 | BCLC Staging System: 0: 30; A: 195; B: 239; C: 54 | | | | - | 255 | 38 | 4 | 112 | 199 | - | - | - | - | - | - | - | 162 | 518 | - | 513 | 5 | - | 164 |
| 56 | Gupta et al. | 2022 | 15 | 28 | 57 | 15 | - | - | - | - | - | - | - | - | - | - | - | - | 66 | 115 | - | - | - | 115 | 29 | - |
| 57 | Hegazy et al. | 2019 | - | - | - | - | - | 29 | 11 | 0 | - | 40 | - | - | - | - | - | - | - | - | - | - | - | - | - | - |
| 58 | Herman et al. | 2016 | - | - | - | - | - | 98 | 3 | 0 | 11 | 34 | - | - | - | - | - | - | - | 74 | 27 | - | 29 | 72 | - | - |
| 59 | Herman et al. | 2014 | - | - | - | - | - | - | - | - | 6 | 17 | - | - | - | - | - | - | - | - | 30 | - | - | - | - | - |
| 60 | Ho et al. | 2021 | BCLC Staging System: A: 46; B: 12 | | | | - | - | - | - | 51 | 6 |  |  |  |  |  | - | - | 58 | 0 | 0 | 43 | 15 | 0 | - |
| 61 | Iancu et al. | 2008 | - | - | - | - | - | - | - | - | - | - | - | - | - | - | - | - | 23 | - | - | - | 29 | 34 | 14 | - |
| 62 | Ibraheem et al. | 2022 | - | - | - | - | 0 | 60 | 27 | 0 | 12 | 72 | - | - | - | - | - | - | - | 87 | 0 | - | 78 | 9 | 0 | - |
| 63 | Jayme et al. | 2021 |  |  |  |  | - | - | - | - | - | - | - | - | - | - | - | 266 | 266 | - | - | - | - | - | - | - |
| 64 | Joshi et al. | 2021 | 28 | 125 | 214 | 20 | 15 | - | - | - | - | - | - | - | - | - | - | - | - | 408 | 0 | 0 | - | 408 | - | - |
| 65 | Kalayarasan et al. | 2013 | 0 | 13 | 96 | 27 | - | - | - | - | - | - | - | - | - | - | - | - | - | 136 | - | - | 16 | 120 | - | - |
| 66 | Kavlakoglu et al. | 2011 | - | - | - | - | - | - | - | - | - | - | - | - | - | - | - | - | - | 42 | 0 | - | 2 | 40 | - | - |
| 67 | Khalil et al. | 2018 | - | - | - | - | - | 65 | 0 | 0 | 1 | 58 | - | - | - | - | - | - | - | 33 | 32 | - | 0 | 65 | - | - |
| 68 | Khuntikeo et al. | 2008 | 0 | 0 | 3 | 26 | - | - | - | - | - | - | - | - | - | - | - | - | - | 30 | - | - | 30 | - | - | - |
| 69 | Kostov et al. | 2013 | - | - | - | - | - | - | - | - | - | - | - | - | - | - | - | 14 | 42 | - | - | - | 29 | 10 | - | 3 |
| 70 | Kostov et al. | 2009 | - | - | - | - | - | - | - | - | - | - | - | - | - | - | - | - | - | 188 | 0 | 0 | 116 | 72 | - | - |
| 71 | Kruger et al. | 2018 | - | - | - | - | - | - | - | - | - | - | - | - | - | - | - | 56 | 35 | 146 | 18 | - | 75 | 89 | - | - |
| 72 | Kumar et al. | 2019 | 12 | 13 | 9 | 0 | - | - | - | - | - | - | - | - | - | - | - | - | 18 | - | - | - | - | 34 | - | - |
| 73 | Leeratanakachorn et al. | 2021 | - | - | - | - | - | - | - | - | - | - | 116 | | - | - | - | - | - | - | - | - | 116 | - | - | - |
| 74 | Long et al. | 2013 | BCLC Staging System: 0: 6; A: 120; B: 47 | | | | - | - | - | - | - | - | - | - | - | - | - | - | - | - | 173 | - | 6 | 163 | - | - |
| 75 | Lopes et al. | 2016 | - | - | - | - | - | 97 | - | - | 11 | 34 | - | - | - | - | - | - | - | 73 | 24 | - | - | - | - | - |
| 76 | Luna-Abanto et al. | 2020 | - | - | - | - | - | - | - | - | - | - | - | - | - | - | - | 31 | - | - | - | - | 2 | 37 | - | - |
| 77 | Machado et al. | 2017 | - | - | - | - | - | - | - | - | - | - | - | - | - | - | - | 15+ | - | 2 | 21 | - | 23 | - | - | - |
| 78 | Makdissi et al. | 2021 | - | - | - | - | - | - | - | - | - | - | - | - | - | - | - | - | - | 30 | - | - | 30 | - | - | - |
| 79 | Makhlouf et al. | 2020 | - | - | - | - | - | 28 | 0 | 0 | 7 | 20 | - | - | - | - | - | - | - | - | - | - | - | - | - | - |
| 80 | Mannai et al. | 2010 | - | - | - | - | - | 22 | 4 | 0 | 9 | 12 | - | - | - | - | - | - | - | - | - | - | 12 | 14 | - | - |
| 81 | Marques et al. | 2018 | - | - | - | - | 24 | - | - | - | - | - | - | - | - | - | - | 91 | - | - | - | - | - | - | - | - |
| 82 | Maurette et al. | 2017 | - | - | - | - | 16 | - | - | - | - | - | - | - | - | - | - | 21 | 30 | 22 | 18 | - | 11 | 29 | - | - |
| 83 | Meira Junior et al. | 2022 | - | - | - | - | 84 | 148 | 5 | - | 26 | 92 | - | - | - | - | - | - | - | 96 | 52 | - | 55 | 127 | - | - |
| 84 | Mogahed et al. | 2021 | - | - | - | - | - | - | - | 0 | - | - | - | - | - | - | - | - | - | 52 | 24 | - | - | - | - | 24 |
| 85 | Molek et al. | 2021 | 31 | 22 | HCC - Stage III/IV: 5  CCA: Stage III: 9; Stage IV: 9 | | - | - | - | - | - | - | - | - | - | - | - | - | - | - | - | - | - | - | - | - |
| 86 | Nag et al. | 2021 | 0 | 34 | 42 | 16 | 2 | - | - | - | - | - | - | - | - | - | - | - | 59 | 77 | 25 | - | 0 | 102 | - | - |
| 87 | Nari et al. | 2018 | - | - | - | - | - | - | - | - | - | - | - | - | - | - | - | - | - | - | - | - | - | 63.02 | - | - |
| 88 | Negi et al. | 2011 | - | - | - | - | - | - | - | - | - | - | - | - | - | - | - |  | 12 | 57 | - | - | 0 | 44 | 5 | - |
| 89 | Nicolas et al. | 2022 | - | - | - | - | - | - | - | - | - | - | - | - | - | - | - | 29 | 48 | 56 | 26 | - | 13 | 69 | - | - |
| 90 | Ninh et al. | 2021 | - | - | - | - | - | 72 | 0 | 0 | 56 | | - | - | - | - | - | - | - | 72 | - | - | 39 | 43 | - | - |
| 91 | Pandey et al. | 2018 | 19 | 57 | 49 | 23 | - | - | - | - | - | - |  |  |  |  |  | 12 | - | - | - | - | 11 | 136 | 6 | - |
| 92 | Panwar et al. | 2016 |  |  |  |  | - | 81 | - | - | 36 | 7 |  |  |  |  |  | - | - | - | - | - | - | - | - | - |
| 93 | Parau et al. | 2015 |  |  |  |  | - | - | - | - | - | - |  |  |  |  |  | 18 | 42-60 | 70 | - | - | 15 | 55 | - | - |
| 94 | Patkar et al. | 2020 |  |  |  |  | - | - | - | - | 59 | - |  |  |  |  |  | - | - | - | - | 8 |  |  | 78 | - |
| 95 | Patkar et al. | 2019 | 12 | 36 | 47 | 2 | - | - | - | - | - | - |  |  |  |  |  |  | 65 | 97 | - | - | - | 97 | - | - |
| 96 | Patkar et al. | 2018 |  |  |  |  | - | - | - | - | - | - | - |  |  |  |  | 83 | 206 | 313 | - | - |  | 313 | - | - |
| 97 | Patkar et al. | 2021 |  |  |  |  | - | - | - | - | - | - |  |  |  |  |  | - | 36 | - | - | - | 18 | 32 |  | - |
| 98 | Petrovic et al. | 2010 |  |  |  |  | - | - | - | - | - | - | - | - | - | - | - | 25 | - | - | - | - | - | - | - | - |
| 99 | Popescu et al. | 2005 |  |  |  |  | - | - | - | - | - | - | - | - | - | - | - | - | - | - | 1 | - | - | - | - | 3 |
| 100 | Popescu et al. | 2012 |  |  |  |  | - | - | - | - | - | - | - | - | - | - | - | 1 | - | - | - | - | - | - | - | 4 |
| 101 | Pottakkat et al. | 2013 | 2 | 5 | 19 | 14 | - | - | - | - | - | - | - | - | - | - | - | - | 38 | - | - | - | 7 | 33 | 10 | - |
| 102 | Quesada-Soto et al. | 2017 | 0 | 3 | 20 | 28 | - | - | - | - | - | - | - | - | - | - | - | 37 | 2 | - | - | - | - | - | - | 7 |
| 103 | Qureshi et al. | 2020 | - | - | - | - | - | - | - | - | - | - | - | - | - | - | - | 120 | - | - | - | - | 95 | 25 | - | - |
| 104 | Rachdi et al. | 2019 |  |  |  |  | - | - | - | - | - | - | - | - | - | - | - | - | - | - | - | - | 7 | 54 | - | 11 |
| 105 | Rammohan et al. | 2015 |  |  |  |  | - | 298 | 128 | - | 68 | 8 | - | - | - | - | - | - | - | - | - | - |  |  | - | - |
| 106 | Ribeiro et al. | 2012 |  |  |  |  | 29 | - | - | - | - | - | - | - | - | - | - | 120 | 112 | - | - | - | 100 | 109 | - | - |
| 107 | Ribeiro et al. | 2013 |  |  |  |  | 29 | - | - | - | - | - | - | - | - | - | - | 120 | 112 | - | - | - | 100 | 109 | - | - |
| 108 | Ruiz et al. | 2022 |  |  |  |  | - |  |  |  | 107 | 4 | - | - | - | - | - | 0 | - | 234 | - | - | 220 | 14 | - | - |
| 109 | Ruiz et al. | 2016 |  |  |  |  | - | 30 | 9 | - | 112 | 7 | - | - | - | - | - | - | - | - | - | - | 193 | 60 | - | - |
| 110 | Rungsakulkij et al. | 2018 | 138 | 79 | | | - | - | - | - | 217 | 7 | - | - | - | - | - | 72 | - | - | - | - | - | - | - | - |
| 111 | Saber et al. | 2020 |  |  |  |  | - | 30 | - | - | - | - | - | - | - | - | - | - | - | 15 | 15 | - | 30 | 0 | - | - |
| 112 | Saglam et al. | 2022 |  |  |  |  | - | - | - | - | 9 | 2 | - | - | - | - | - | 1 | 8 | 30 | 1 | - | 20 | 11 | - | - |
| 113 | Said et al. | 2021 |  |  |  |  | - | 227 | 3 | 0 | 1 | 214 | - | - | - | - | - | - | - | 230 | - | - | 52 | 178 | - | 12 |
| 114 | Sapmaz et al. | 2020 |  |  |  |  | - | - | - | - | - | - | - | - | - | - | - | - | - | 20 | - | - | - | - | - | - |
| 115 | Saritas et al. | 2020 |  |  |  |  | 9 | - | - | - | - | - | - | - | - | - | - | 18 | 50 | - | - | - | - | - | 34 | - |
| 116 | Sawangkajohn et al. | 2020 |  |  |  |  | - | - | - | - | - | - | - | - | - | - | - | - | - | - | - | - | 111 | - | - | - |
| 117 | Senbel et al. | 2017 |  |  |  |  | - | 84 | 0 | 0 | 21 | 63 | - | - | - | - | - | - | - | - | - | - | - | - | - | - |
| 118 | Shehta et al. | 2021 |  |  |  |  | - | 261 | 7 | - | 3 | 246 | - | - | - | - | - | - | - | 264 | 4 | - | 58 | 210 | - | 14 |
| 119 | Shehta et al. | 2021 |  |  |  |  | - | 281 | 7 | 0 | 3 | 265 | - | - | - | - | - | - | - | 284 | 4 | - | 67 | 221 | - | 19 |
| 120 | Shehta et al. | 2020 |  |  |  |  | - |  |  |  |  |  | - | - | - | - | - | - | - |  |  | - | 172 | 91 | - | - |
| 121 | Soliman et al. | 2017 |  |  |  |  | - | 38 | - | - | - | - | - | - | - | - | - | - | - | 2 | 36 | - | - | 38 | - | - |
| 122 | Sriputtha et al. | 2013 | 1 | 3 | 9 | 38 | - | - | - | - | - | - | - | - | - | - | - | - | - | - | - | - | - | - | - | - |
| 123 | Taesombat et al. | 2020 | - | - | - | - | 0 | - | - | - | - | - | - | - | - | - | - | 6 | - | 24 | 12 | - | - | 36 | - | - |
| 124 | Taesombat et al. | 2020 | - | - | - | - | 4 | 54 | 0 | 0 | 34 | 6 | - | - | - | - | - | - | - | 24 | 30 | - | - | - | - | - |
| 125 | Takorov et al. | 2016 | - | - | - | - | - | - | - | - | - | - | - | - | - | - | - | - | - | - | - | - | 5 | 22 | 27 | - |
| 126 | Techathuvanan et al. | 2015 | - | - | - | - | - | - | - | - | 215 | 0 | - | - | - | - | - | - | - | - | - | - | - | - | - | - |
| 127 | Tohra et al. | 2021 | - | - | - | - | - | - | - | - | 18 | 8 | - | - | - | - | - | - | - | 38 | - | - | - | - | - | - |
| 128 | Tomas et al. | 2020 | - | - | - | - | - | 38 | 4 | - | 36 | 12 | - | - | - | - | - | - | - | - | - | - | 17 | 37 | - | - |
| 129 | Tongsiri et al. | 2020 | - | - | - | - | - | - | - | - | 1 | 2 | 38 | 2 | - | - | - | - | - | 40 | - | - | 40 | - | - | - |
| 130 | Valadares et al. | 2015 | - | - | - | - | - | - | - | - | - | - | - | - | - | - | - | - | - | - | - | - | 9 | 13 | 11 | - |
| 131 | VazdaSilva et al. | 2020 | - | - | - | - | 37 | - | - | - | - | - | - | - | - | - | - | 166 | 182 | - | - | - | 171 | 109 | - | - |
| 132 | Wahab et al. | 2014 | 133 | 31 | 41 | - | - | 183 | 25 | - |  | 208 | - | - | - | - | - | - | - | - | - | - | 73 | 135 | - | - |
| 133 | Wahab et al. | 2012 | - | - | - | - | - |  |  | 0 | 2 | 58 | - | - | - | - | - | - | - | - | - | - | 159 |  | - | - |
| 134 | Younes et al. | 2020 | - | - | - | - | - | - | - | - | - | - | - | - | - | - | - | - | - | - | - | - | - | - | - | - |
| 135 | Zakaria et al. | 2020 | - | - | - | - | - | 286 | 0 | 0 | 19 | 264 | - | - | - | - | - | - | 6 | 260 | 26 | - | - | - | - | - |

**Section 4: Extended Results, Forest Plots, and Funnel Plots**

**Supplementary Table:** Studies Pooled For Each Outcome

| **Outcome** | **Studies Pooled (Study IDs)** |
| --- | --- |
| **Operative Duration** | 1, 5, 6, 7, 12, 13, 19, 20, 22, 23, 24, 27, 29, 30, 33, 35, 36, 40, 42, 43, 44, 46, 48, 51, 52, 59, 61, 66, 69, 71, 73, 76, 77, 81, 82, 83, 85, 86, 88, 90, 105, 111, 113, 119, 121, 123, 125, 130, 132, 133, 135 |
| **Margin Status** | 3, 5, 7, 9, 12, 13, 16, 17, 18, 22, 23, 25, 27, 28, 30, 32, 33, 36, 38, 40, 42, 44, 47, 52, 55, 61, 62, 65, 66, 68, 69, 70, 71, 77, 83, 84, 85, 86, 87, 89, 91, 92, 97, 101, 106, 110, 113, 118, 119, 122, 123, 124, 125, 126, 130, 131, 132, 133 |
| **Return to Operating Room** | 3, 5,6, 13, 14, 17, 22, 23, 24, 33, 35, 58, 59, 63, 66, 68, 69, 74, 77, 92, 113, 129 |
| **Unplanned Post-Operative Intubation** | 94, 116 |
| **Endoscopic or Percutaneous Reintervention** | 13, 14, 22, 68, 95, 125, 129, 130 |
| **Readmission** | 3, 43, 44, 63, 70 |
| **Length of Stay** | 1, 5, 10, 12, 15, 19, 20, 21, 23, 24, 29, 30, 33, 35, 36, 38, 40, 42, 43, 44, 45, 63, 66, 69, 77, 87, 89, 90, 94, 96, 111, 113, 115, 116, 123, 124, 125, 126, 127, 130, 132, 135 |
| **In-Hospital Morbidity** | 3, 14, 20, 22, 24, 46, 57, 92, 94, 100, 119, 129, 130 |
| **30-Day Morbidity** | 11, 17, 37, 93 |
| **90-Day Morbidity** | 41, 67, 74, 76, 96 |
| **Clavien-Dindo Grade 1-2** | 13, 22, 30, 36, 38, 43, 44, 48, 57, 61, 63, 66, 70, 75, 85, 86, 87, 88, 90, 94, 113, 119, 123, 124, 125, 131, 135 |
| **Clavien-Dindo Grade >2** | 13, 17, 22, 23, 24, 27, 28, 30, 36, 38, 40, 43, 44, 48, 57, 61, 63, 66, 70, 77, 81, 85, 86, 87, 88, 90, 94, 96, 113, 119, 123, 124, 125, 131, 135 |
| **In-Hospital Mortality** | 3, 5, 13, 14, 17, 24, 32, 42, 48, 50, 58, 60, 83, 92, 94, 97, 101, 118, 119, 132 |
| **30-Day Mortality** | 2, 11, 17, 19, 20, 24, 27, 30, 31, 35, 36, 37, 42, 44, 45, 46, 50, 51, 52, 53, 61, 65, 68, 69, 72, 87, 90, 92, 93, 97, 107, 108, 128 |
| **90-Day Mortality** | 2, 27, 35, 39, 40, 42, 46, 68, 77, 107, 108 |
| **Liver Failure** | 1, 5, 11, 13, 14, 19, 21, 23, 24, 25, 29, 31, 35, 36, 40, 41, 42, 43, 44, 45, 47, 49, 52, 54, 56, 58, 61, 63, 67, 69, 72, 76, 77, 79, 81, 82, 83, 87, 90, 91, 92, 93, 94, 101, 107, 109, 111, 113, 115, 116, 117, 118, 119, 123, 129, 130, 132 |
| **Bile Leaks** | 1, 3, 4, 5, 13, 17, 18, 21, 22, 23, 24, 28, 29, 40, 41, 43, 44, 45, 50, 52, 55, 59, 61, 63, 68, 69, 71, 72, 73, 74, 77, 82, 83, 85, 86, 87, 88, 90, 91, 93, 94, 99, 101, 109, 111, 112, 113, 116, 117, 118, 119, 121, 123, 125, 128, 129, 132 |
| **Hemorrhage** | 1, 3, 5, 11, 13, 14, 23, 24, 25, 28, 36, 40, 41, 43, 44, 45, 50, 58, 60, 61, 67, 68, 77, 78, 79, 82, 88, 97, 99, 101, 109, 111, 113, 115, 118, 119, 129 |
| **Myocardial Infarction** | 11, 25, 41, 101, 115 |
| **Thrombotic or Thromboembolic Events** | 3, 11, 40, 41, 65, 69, 72, 82, 83, 91, 109, 113, 117, 128, 129 |
| **Cerebrovascular Accidents** | 69, 109, 120 |
| **Pneumonia** | 11, 13, 14, 22, 24, 57, 68, 72, 79, 109, 111, 117, 120, 123, 128, 130 |
| **Liver Abscess** | 11, 17, 21, 22, 41, 47, 52, 60, 74, 81, 87, 93, 99, 109, 117, 119 |
| **Wound Dehiscence or Infection** | 1, 3, 5, 14, 18, 19, 21, 22, 36, 41, 42, 43, 44, 45, 55, 60, 63, 66, 67, 68, 69, 71, 74, 77, 78, 81, 82, 83, 87, 88, 91, 101, 109, 111, 115, 118, 119, 123, 125, 128, 129, 132 |
| **Urinary Tract Infections** | 18, 123, 130 |
| **Bacteremia** | 22, 28, 57, 59, 69, 99 |
| **Other Infections** | 1, 5, 13, 14, 18, 24, 25, 35, 36, 42, 43, 44, 45, 49, 54, 59, 63, 65, 68, 76, 77, 81, 82, 90, 92, 93, 99, 101, 115, 117, 118, 123, 130, 132 |
| Study IDs are consistent with the serial numbers given in Supplementary Tables A and B in Supplementary Section 3.  Studies 27, 30, 40, 43, 44, 86, and 123 each included 2 cohorts of patients that were pooled in the analysis separately. Study 87 included 4 cohorts of patients that were pooled in the analysis separately | |

**Subsection A: Extended Results**

**Narrative Results**

*Low-Income Countries*

Four studies from LICs (Ethiopia, Uganda, Sudan, and Syria) were evaluated during the full-text screening stage of the review. Three of these studies were excluded as 2 were review articles without original outcomes data while the third did not report the outcomes of patients who had undergone oncologic hepatectomies separately from other patients. Thus, only a single study by Elsanousi et al. from Sudan met the inclusion criteria. Their cohort included 44 patients who underwent hepatectomy for the treatment of HCC. The cohort was primarily male (72.3%) and included 21 patients with cirrhosis. Sixteen (36.4%) patients experienced major complications including 4 surgical site infections (SSI), 2 bile leaks, 1 pulmonary embolism, 2 cases of liver failure, and 1 intra-abdominal abscess. Four (9.1%) patients died within 30 days of surgery, 1 from a pulmonary embolism, 2 from liver failure, and 1 from respiratory failure.

*Studies of Pediatric Populations*

Two studies investigated outcomes in pediatric patients undergoing the surgical management of hepatoblastoma. The first study by Qureshi et al. compared the outcomes of 120 children in India who underwent anatomic resection (n=95) versus non-anatomic resection (n=25) for hepatoblastoma. Duration of surgery and blood loss were found to be significantly higher in the cohort undergoing anatomic resection. Overall, 24 (20%) patients experienced complications, including 22 from the anatomic resection group and 2 from the non-anatomic resection group. These included eight bile leaks, of which seven occurred in the anatomic resection group and one in the non-anatomic resection group. However, neither of these differences achieved significance. Length of stay and margin status were also reported to be similar across the two groups. The second study by Younes et al. investigated factors associated with local disease recurrence after the resection of hepatoblastoma. One hundred and thirty-three pediatric patients underwent surgery in their cohort, of whom 9 (7%) experienced postoperative complications. There were 2 patients who experienced bile leaks and 2 who experienced obstructive jaundice within 90 days of surgery. Five (4%) patients experienced adhesive small bowel obstructions. Twenty-nine (22%) patients had positive (R1) margins.

**Meta-Analysed Results**

| **Supplementary Table:** Summary of Pooled Outcomes with Subgroup Comparisons Between upperMICs and lowerMICs | | | | | | | | |
| --- | --- | --- | --- | --- | --- | --- | --- | --- |
| **Variables** | **Number of Cohorts** | **Total Patients** | **Pooled Outcome (95% CI)** | | | **P-value (Test for Subgroup Differences)** | **I^2^** | **𝜏^2^** |
|  |  |  | **Overall** | **lowerMIC** | **upperMIC** |  |  |  |
| **Procedure Time** | | | | | | | | |
| Operative Duration (Minutes) | 45 | 5472 | 200.34 (173.02, 227.65) | 185.24 (154.98, 215.49) | 259.74 (203.87, 315.62) | **0.02** | 81.07 | 4448.87 |
| **Margin Status** | | | | | | | | |
| Positive Margins | 66 | 8739 | 0.09 (0.06, 0.12) | 0.08 (0.04, 0.13) | 0.09 (0.06, 0.14) | 0.57 | 95.54 | 0.16 |
| **Postoperative Course** | | | | | | | | |
| Return to OR | 22 | 3002 | 0.03 (0.02, 0.04) | 0.03 (0.01, 0.05) | 0.03 (0.02, 0.05) | 0.69 | 61.41 | 0.01 |
| Unplanned Intubation | 2 | 632 | 0.02 (0.01, 0.04) | 0.03 (0.01, 004) | 0.01 (0.00, 0.04) | 0.27 | 31.29 | 0.00 |
| Percutaneous or Endoscopic Intervention | 8 | 331 | 0.05 (0.02, 0.10) | 0.01 (0.00, 0.04) | 0.07 (0.04, 0.12) | **0.01** | 43.98 | 0.02 |
| Readmission | 7 | 917 | 0.05 (0.02, 0.10) | 0.05 (0.02, 0.09) | 0.10 (0.06, 0.15) | 0.09 | 73.56 | 0.03 |
| Length of Stay (Days) | 45 | 6950 | 8.33 (6.81, 9.85) | 7.69 (5.61, 9.76) | 9.33 (7.09, 11.58) | 0.29 | 78.39 | 11.07 |
| **Morbidity** | | | | | | | | |
| In-Hospital Morbidity | 13 | 1802 | 0.27 (0.14, 0.42) | 0.27 (0.08, 0.52) | 0.26 (0.10, 0.46) | 0.99 | 97.53 | 0.31 |
| 30-Day Morbidity | 4 | 921 | 0.28 (0.16, 0.40) | - | - | - | 89.10 | 0.06 |
| 90-Day Morbidity | 5 | 586 | 0.34 (0.13, 0.58) | 0.13 (0.10, 0.17) | 0.40 (0.15, 0.68) | **0.03** | 96.61 | 0.30 |
| Clavien-Dindo 1 and 2 | 34 | 5240 | 0.27 (0.19, 0.37) | 0.34 (0.26, 0.43) | 0.22 (0.11, 0.37) | 0.17 | 97.96 | 0.32 |
| ≥ Clavien-Dindo 3 | 44 | 6279 | 0.11 (0.08, 0.14) | 0.11 (0.08, 0.14) | 0.12 (0.07, 0.17) | 0.68 | 92.30 | 0.09 |
| **Mortality** | | | | | | | | |
| In-Hospital Mortality | 20 | 2752 | 0.05 (0.02, 0.09) | 0.07 (0.02, 0.16) | 0.02 (0.00, 0.04) | 0.12 | 94.31 | 0.13 |
| 30-Day Mortality | 39 | 5633 | 0.02 (0.01, 0.03) | 0.02 (0.00, 0.03) | 0.02 (0.01, 0.03) | 0.93 | 77.21 | 0.02 |
| 90-Day Mortality | 13 | 1097 | 0.03 (0.01, 0.05) | 0.02 (0.00, 0.04) | 0.04 (0.02, 0.08) | 0.10 | 59.20 | 0.02 |
| **Specific Complications** | | | | | | | | |
| Liver Failure | 64 | 8345 | 0.08 (0.06, 0.12) | 0.13 (0.07, 0.20) | 0.06 (0.03, 0.08) | **0.03** | 95.46 | 0.16 |
| Bile Leak | 64 | 8004 | 0.06 (0.04, 0.08) | 0.06 (0.04, 0.09) | 0.05 (0.03, 0.09) | 0.74 | 89.67 | 0.07 |
| Hemorrhage | 40 | 4035 | 0.03 (0.02, 0.04) | 0.04 (0.02, 0.06) | 0.02 (0.01, 0.04) | 0.11 | 62.29 | 0.02 |
| Myocardial Infarction | 5 | 441 | 0.02 (0.01, 0.04) | 0.05 (0.00, 0.14) | 0.02 (0.00, 0.03) | 0.22 | 15.54 | 0.00 |
| Thrombotic or Thromboembolic Event | 16 | 1972 | 0.02 (0.01, 0.03) | 0.02 (0.01, 0.03) | 0.02 (0.01, 0.04) | 0.39 | 40.60 | 0.01 |
| Cerebrovascular Accident | 3 | 709 | 0.01 (0.00, 0.01) | 0.00 (0.00, 0.02) | 0.01 (0.00, 0.02) | 0.63 | 10.23 | 0.00 |
| Pneumonia | 17 | 1414 | 0.04 (0.02, 0.06) | 0.06 (0.0, 0.12) | 0.03 (0.01, 0.06) | 0.41 | 66.51 | 0.03 |
| Liver Abscess | 19 | 3210 | 0.03 (0.01, 0.05) | 0.02 (0.00, 0.05) | 0.03 (0.01, 0.05) | 0.56 | 79.99 | 0.02 |
| Wound Dehiscence or Infection | 48 | 5922 | 0.06 (0.04, 0.08) | 0.09 (0.06, 0.13) | 0.04 (0.02, 0.06) | **<0.01** | 86.09 | 0.05 |
| Urinary Tract Infection | 4 | 119 | 0.05 (0.01, 0.10) | - | - | - | 8.71 | 0.00 |
| Bacteremia | 6 | 448 | 0.01 (0.00, 0.03) | 0.02 (0.00, 0.05) | 0.02 (0.00, 0.04) | 0.91 | 16.01 | 0.00 |
| Unspecified Infection | 37 | 3629 | 0.07 (0.05, 0.10) | 0.10 (0.06, 0.15) | 0.05 (0.03, 0.07) | **0.02** | 82.76 | 0.05 |
| *Abbreviations: CI: Confidence Interval; lowerMIC: Lower-Middle Income Country; upperMIC: Upper-Middle Income Country; OR: Operating Room*  *Red indicates a significant p-value (<0.05)* | | | | | | | | |

**Operative Duration**

The pooled mean operative duration was 200.34 minutes (95% CI: 173.02, 227.65; K (number of pooled cohorts) = 45, N (number of pooled patients) = 5472) and varied significantly across income status (p=0.02) and country (p=0.01) subgroups. Pooled operative time was 259.74 (95% CI: 203.87, 315.62; K=15, N=2178) in upperMICs and 185.24 (95% CI: 154.98, 215.49; K=30, N= 3294) in lowerMICs. The mean operative time in Brazilian studies was a high outlier while Egyptian articles reported significantly shorter operative durations. Operative time did not significantly vary across indication subgroups (p=0.17).

**Margin Status**

The overall rate of positive margins was 0.09 (95% CI: 0.06, 0.12; K = 66, N = 8739). Subgroup meta-analysis showed a rate of 0.08 (95% CI: 0.04, 0.13; K=27, N= 2898) in lowerMICs and 0.09 (95% CI: 0.06, 0.14; K=39, N=5841) in upperMICs and this difference did not achieve significance (p=0.57). Significant variation between individual countries’ results was seen (p<0.01). India and Argentina had significantly lower and higher rates of positive margins respectively. Margin status also varied significantly across indication subgroups (p<0.01). Cholangiocarcinoma and Neuroendocrine tumours had significantly higher rates of positive margins while the gallbladder cancer subgroup had a significantly lower rate versus the overall result.

**Post-Operative Course**

*Return to Operating Room*

The overall rate of return to the operating room (OR) was computed to be 0.03 (95% CI: 0.02, 0.04; K=22, N=3002). Subgroup meta-analysis showed a rate of 0.03 (95% CI: 0.01, 0.05; K=8, N=1360) in lowerMICs and 0.03 (95% CI: 0.02, 0.05; K=14, N=1642) in upperMICs. These rates were not significantly different (p=0.69). Individual countries’ results did not vary significantly (p=0.50) nor did any country’s results significantly differ from the overall result. However, the test for subgroup differences across indications was significant (p=0.01) and the rate was lower versus the overall result for the gallbladder cancer subgroup.

*Unplanned Post-Operative Intubation*

Only 2 studies reported rates of unplanned intubation after surgery. One article from India had a rate of 0.03 (95% CI: 0.01, 0.04, N=516) while an article from Thailand had a rate of 0.01 (95% CI: 0.00, 0.04, N=116). These rates were not significantly different (p=0.27) and a pooled rate of 0.02 (95% CI: 0.01, 0.04, N=632) was obtained.

*Endoscopic or Percutaneous Intervention*

The overall rate of endoscopic or percutaneous intervention after surgery was 0.05 (95% CI: 0.02, 0.10; K=8, N=331). The difference between upperMICs (0.07 [95% CI: 0.04, 0.12], K=7, N=234) and lowerMICs (0.01 [95% CI: 0.00, 0.04], K=1, N=97) was significant (p=0.01) but only 1 study from India represented the lowerMIC subgroup. Significant variation was also seen across subgroups by indication (p=0.05) and the rate was lower versus overall in the gallbladder cancer subgroup which consisted of the same single article from India.

*Readmission*

Seven studies reported readmission rates and a pooled result of 0.05 (95% CI: 0.02, 0.10, N=917) was computed. Six studies were from lowerMICs, which had a pooled rate of 0.05 (95% CI: 0.02, 0.09, N=741) while the study from Brazil, a upperMIC, had a rate of 0.05 (95% CI: 0.06, 0.15, N=176). The test for subgroup differences by income status was significant (p=0.09). Subgrouping by country showed that the rate of readmission in the sole study from Brazil was a high outlier. Subgrouping by indication also showed significant differences (p=0.01)

*Length of Stay*

Forty-five studies were pooled and demonstrated a mean length of stay of 8.33 days (95% CI: 6.81, 9.85, N= 6950). Twenty-one studies were from lowerMICs, which had a pooled rate of 7.69 days (95% CI: 5.61, 9.76, N=2952). Twenty-four studies were from upperMICs and had a rate of 9.33 days (95% CI: 7.09, N=3998). These rates were not significantly different (p=0.29). Results did not vary significantly across countries (p=0.48). However, the pooled length of stay in the cholangiocarcinoma subgroup was significantly longer than for other tumor types.

**Morbidity**

*In-Hospital Morbidity*

In-hospital morbidity rates across 13 studies were meta-analysed and a rate of 0.27 (95% CI: 0.14, 0.42, N=1802) was obtained. LowerMICs had an in-hospital morbidity rate of 0.27 (95% CI: 0.08, 0.52; K=6, N=1238) and upperMICs had a rate of 0.26 (95% CI: 0.10, 0.46; K=7, N=564). This difference did not reach significance (p=0.99). Rates varied significantly across national subgroups (p<0.01). Thailand reported a low rate while those of Argentina, Romania, and Serbia were high outliers. Significant differences across subgroups by indication were also seen (p<0.01). The rate of in-hospital morbidity was high in the colorectal cancer liver metastases group and low in the neuroendocrine metastases and cholangiocarcinoma subgroups. However, these two subgroups only included a single study each.

*30-Day Morbidity*

Four studies from Romania reported this outcome. A pooled rate of 0.28 (95% CI: 0.16, 0.40, N=921) was obtained.

*90-Day Morbidity*

A pooled rate of 90-day morbidity was computed to be 0.34 (95% CI: 0.13, 0.58; K=5, N=586). The pooled rate in upperMICs was 0.40 (95% CI: 0.15, 0.68; K=4, N=273), which was significantly higher (p=0.03) than the rate of 0.13 (95% CI: 0.10, 0.17; K=1, N=313) reported from India, a lowerMIC. Significant variation was also seen across country (p=0.00) and indication (p=0.00) although the number of pooled studies was small.

*Clavien-Dindo Grade 1 or 2*

The rate of Clavien-Dindo grade 1 or 2 morbidity in patients was found to be 0.27 (95% CI: 0.19, 0.37, K=34, N=5240). Subgroup results for lowerMICs (0.34 [95% CI: 0.26, 0.43], K=15, N=2269) and upperMICs (0.22 [95% CI: 0.11, 0.37], K=19, N=2971) were not significantly different (p=0.17). Significant differences were seen between the results of individual countries (p<0.01). Argentina, Bulgaria, Thailand, and Turkiye had lower rates compared to the pooled result while Egypt, Serbia, Vietnam, and Peru had relatively higher rates. Rates also significantly varied across indication (p=0.00) although this was largely due to the outlier result in the single study investigating liver metastases of ovarian cancer.

*Clavien-Dindo Grade ≥3*

The rate of Clavien-Dindo grade 3, 4, or 5 complications was pooled across 44 studies and found to be 0.11 (95% CI: 0.08, 0.14, N=6279). Subgroup results for lowerMICs (0.11 [95% CI: 0.08, 0.14], K=20, N=2933) and upperMICs (0.12 [95% CI: 0.07, 0.17], K=24, N=3346) were not significantly different (p=0.68). However, the results varied significantly across individual countries (p=0.02). Vietnam (0.03 [95% CI: 0.00, 0.08], K=1, N=72) had rates significantly lower than the overall result, and Ukraine, which had a significantly higher rate (0.20 [95% CI: 0.12, 0.29], K=2, N=185). Variations were also seen across subgroups by indication (p=0.03). The gallbladder cancer subgroup had a significantly lower rate of major complications while cholangiocarcinoma had a significantly higher rate.

**Mortality**

*In-Hospital Mortality*

Twenty studies were pooled to compute an overall in-hospital mortality rate of 0.05 (95% CI: 0.02, 0.09, N=2752). The difference between upperMIC (0.02 [0.00, 0.04], K=9, N=834) and lowerMIC (0.07 [0.02, 0.16], K= 11, N=1918) subgroups did not achieve significance (p=0.12). Significant differences were seen across subgroups by country (p=0.05), where the Turkiye subgroup had a lower rate than the overall rate. No significant variation was seen by indication (p=0.58).

*30-Day Mortality*

Thirty-nine studies reported rates of 30-day mortality and a meta-analysed rate of 0.02 (95% CI: 0.01, 0.03, N=5633) was obtained. No difference (p=0.93) was seen between lowerMIC (0.02 [0.00, 0.03], K=17, N=1253) and upperMIC (0.02 [0.01, 0.03], K=22, N=4380) subgroups. Significant differences were seen across country subgroups (p=0.01) where Argentinian studies had a lower rate of 30-day mortality while the Serbian and Turkish subgroups reported higher rates. No significant variation was seen by indication (p=0.89).

*90-Day Mortality*

Thirteen studies reported this outcome and an overall rate of 0.03 (95% CI: 0.01, 0.05, N=1097) was obtained. The difference between lowerMICs (0.02 [0.00, 0.04], K=5, N=407) and upperMICs (0.04 [0.02, 0.08], K=8, N=690) did not reach significance (p=0.10).

**Specific Postoperative Complication Rates**

*Liver Failure or Dysfunction*

Sixty-four studies were pooled to compute an overall postoperative liver failure rate of 0.08 (0.06, 0.12, N=8345). Studies from lowerMICs (0.13 [0.07, 0.20], K=28, N=3361) reported significantly higher rates of post-operative liver failure (p=0.03) than upperMICs (0.06 [0.03, 0.08], K=36, N=4984). Significant variation by country subgroups was seen (p=0.00) with rates that were high in studies from Mongolia and Tunisia and low rates in Bulgaria, Pakistan, Turkiye, and Vietnam. Significant variation was also seen across indications (p=0.00). The gallbladder cancer and colorectal cancer liver metastases subgroups had significantly lower rates than the overall result.

*Bile Leaks*

A meta-analysis of 64 studies showed the rate of postoperative bile leaks to be 0.06 (95% CI: 0.04, 0.08, N=8004). The rates for lowerMICs (0.06 [0.04, 0.09], K=34, N=4011) and upperMICs (0.05 [0.03, 0.09], K=30, N=3993) did not significantly differ (p=0.74). Subgrouping by country showed significant differences (p=0.00). Peruvian and Vietnamese studies had significantly lower rates than the overall result while a study from Russia had a high outlier result.

*Hemorrhage*

Forty studies were pooled, and the overall rate of postoperative hemorrhage was computed to be 0.03 (95% CI: 0.02, 0.04, N=4035). The difference between lowerMICs (0.04 [0.02, 0.06], K=20, N=2001) and upperMICs (0.02 [0.01, 0.03], K=20, N=2034) did not achieve significance (p=0.11). Results did not vary across country (p=0.61) or indication (p=0.51) subgroups.


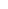


*Myocardial Infarction*

Five studies reported the rates of myocardial infarction after surgery and an overall rate of 0.02 (95% CI: 0.01, 0.04, N=441) was computed. No difference (p=0.22) between upperMICs (0.02 [0.00, 0.03], K=4, N=401) and lowerMICs (0.05 [0.00, 0.14], K=1, N=40) was seen. No difference was seen across countries (p=0.76) or indications (p=0.47).

*Thrombotic or Thromboembolic Events*

Sixteen studies were pooled to calculate the overall rate of thrombotic or thromboembolic events after surgery, which was found to be 0.02 (95% CI: 0.01, 0.03, N=1972). No differences (p=0.39) were seen between lowerMICs (0.02 [0.01, 0.03], K=5, N=713) and upperMICs (0.02 [0.01, 0.04], K= 11, N=1259). No differences were seen between country (p=0.61) and indication subgroups (p=0.40).

*Cerebrovascular Accidents*

Three studies reported data on strokes and cerebrovascular accidents after surgery and the overall rate was computed to be 0.01 ([0.00, 0.01], N=709). No significant difference (p=0.63) was seen between lowerMICs (0.00 [0.00, 0.02], K=1, N=268) and upperMICs (0.01 [0.00, 0.02], K=2, N=441) nor did the results vary across the three countries (p=0.64) or indications (p=0.35).

*Pneumonia*

Seventeen studies were pooled to compute an overall post-operative pneumonia rate of 0.04 (95% CI: 0.02, 0.06, N=1414). No difference (p=0.41) was seen between lowerMICs (0.06 [0.01, 0.12], K=4, N=408) and upperMICs (0.03 [0.01, 0.06], K=13, N=1006). Studies from Peru and Romania had significantly lower rates of post-operative pneumonia than other countries. Significant variations across indication subgroups were also seen (p=0.03).

*Liver Abscesses*

Nineteen studies reported rates of post-operative liver abscesses. An overall rate of 0.03 (95% CI: 0.01, 0.05, N=3210) was obtained and the difference between lowerMICs (0.02 [0.00, 0.05], K=3, N=408) and upperMICs (0.03 [0.01, 0.05], K=16, N=2802) did not reach significance (p=0.56). No significant differences in results across country (p=0.71) or indication (p=0.94) were seen.

*Wound Dehiscence or Infection*

Using data from 48 studies, meta-analysis computed an overall wound dehiscence rate of 0.06 ([0.04, 0.08], N=5922). LowerMICs (0.09 [0.06, 0.13], K=23, N=2494) had significantly higher (p<0.01) rates than upperMICs (0.04 [0.02, 0.06], K=25, N=3428). Subgrouping by country also showed significant variation (p=0.00). Studies from Argentina, Peru, and Russia reported significantly lower rates compared to other countries. Among indication subgroups rates varied significantly (p=0.02) and were higher in the cholangiocarcinoma subgroup and lower rates in the hepatocellular carcinoma and colorectal cancer liver metastases subgroups.

*Urinary Tract Infections*

Four studies were pooled to calculate an overall rate of 0.05 ([0.01, 0.10], K=4, N=119) for post-operative urinary tract infections. Results were comparable (p=0.50) across both countries and indications.

*Bacteremia*

Six studies were pooled, and the rate of post-operative bacteremia was computed to be 0.01 (95% CI: 0.00, 0.03, N=448). LowerMICs (0.02 [0.00, 0.05], K=2, N=124) and upperMICs (0.02 [0.00, 0.04], K=3, N=324) did not significantly differ (p=0.91). Differences across country (p=0.48) and indication (p=0.40) subgroups were not significant.

*Other Infections*

Additional infective complications, such as infected intraabdominal fluid collections, were reported by 37 studies. Pooled analysis yielded a rate of 0.07 (95% CI: 0.05, 0.10, N=3629). Studies from lowerMICs reported higher rates (0.10 [0.06, 0.15], K=16, N=1753) than those from upperMICs (0.05 [0.03, 0.07], K=21, N=1876) and this difference was significant (p=0.02). Results were significantly variable across countries (p<0.01). These included lower rates reported by Brazil, Romania, and Vietnam. Egyptian studies had significantly higher rates than other countries. Among subgroups by indication, the cholangiocarcinoma and colorectal cancer subgroups were high and low outliers respectively.

**Subsection B: Forest Plots**


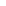


Forest plots depict the meta-analysis of each outcome. The total number of cohorts (K) and patients (N) is reported. All plots depict the overall result and subgroup analysis by national income status (income), country of publication (country) and tumor type (indication).

**Supplementary Forest Plot 1:** Operative Duration


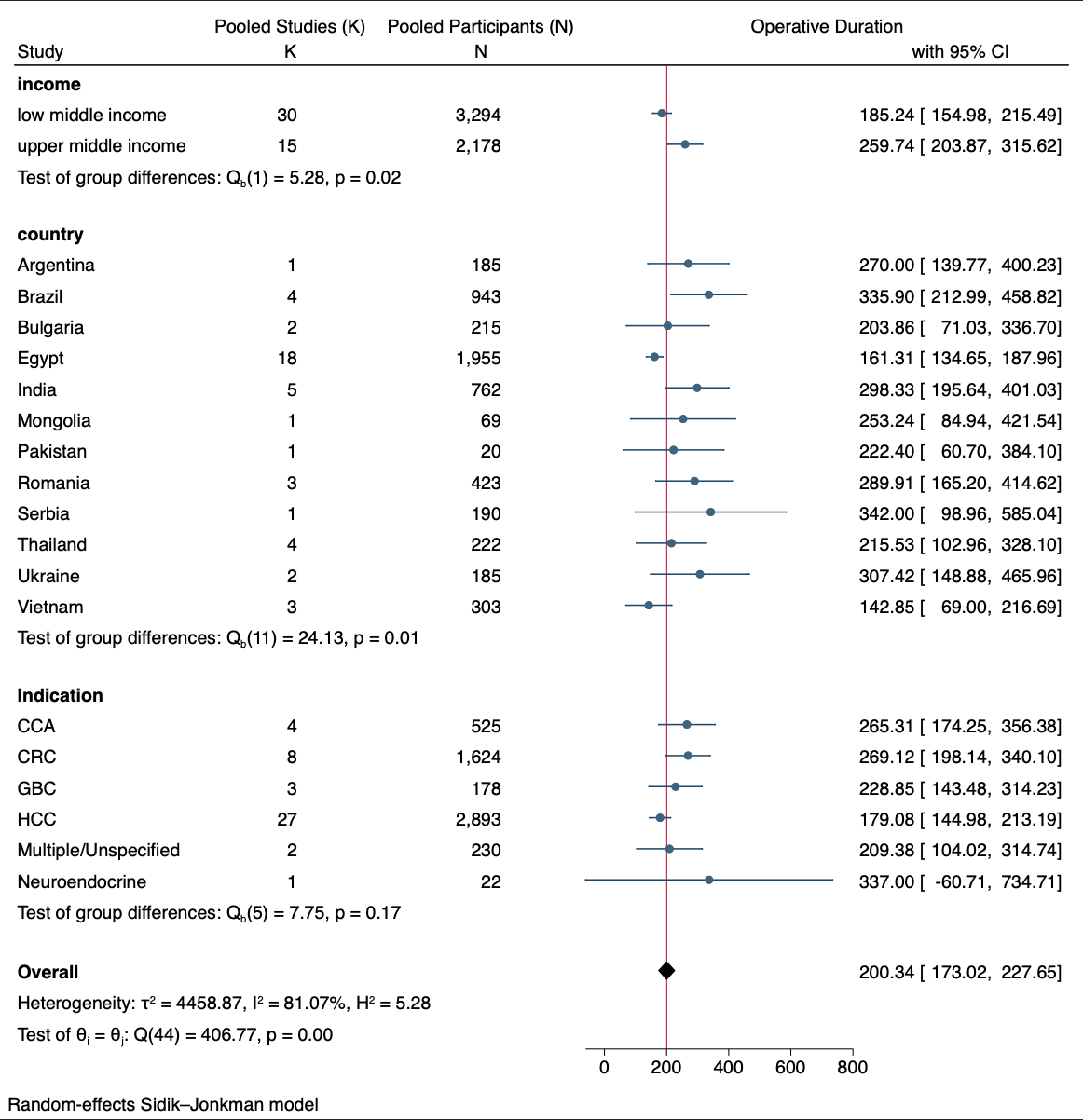


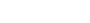


**Supplementary Forest Plot 2:** Margin Status


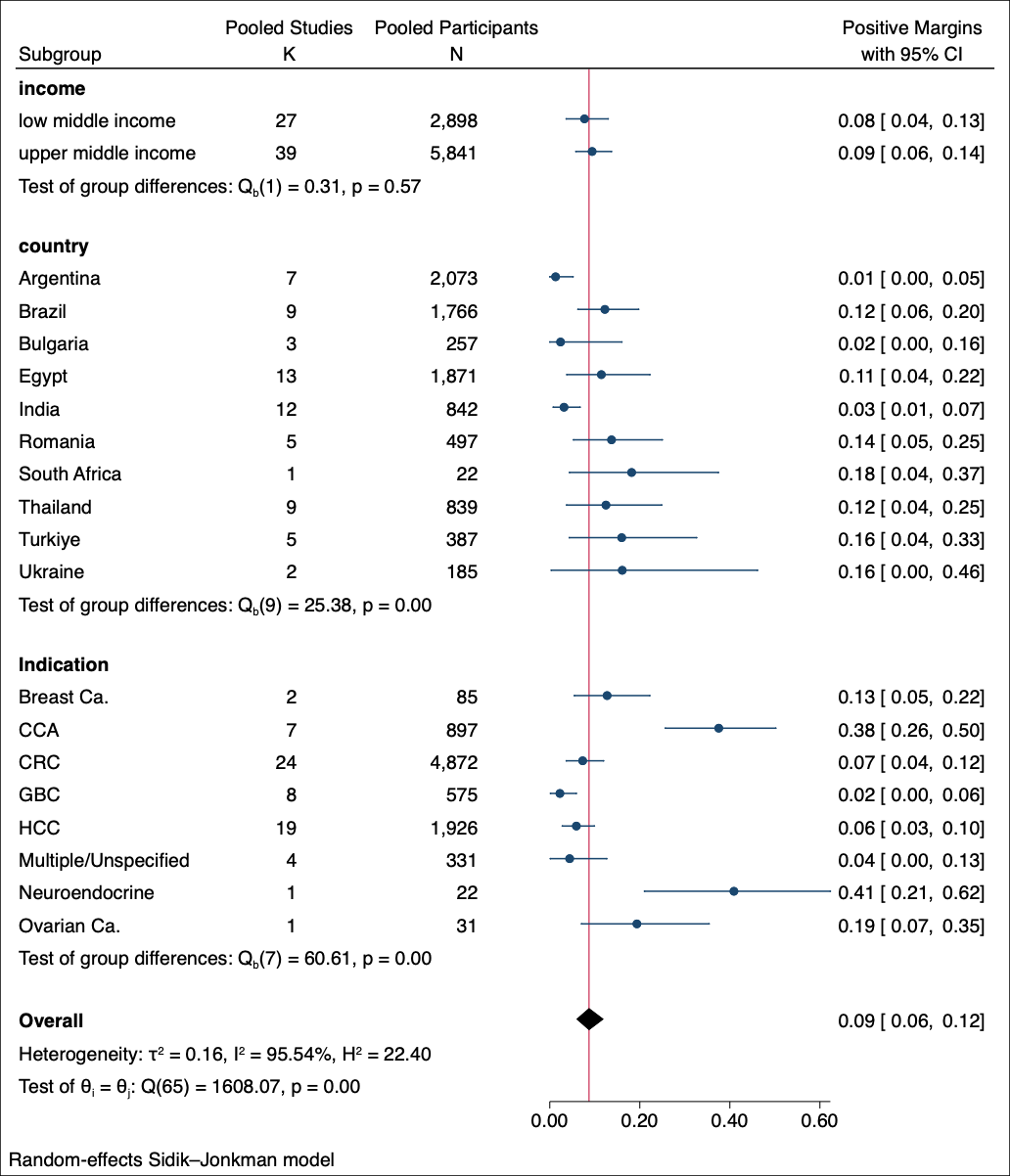


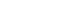


**Supplementary Forest Plot 3:** Return to Operating Room


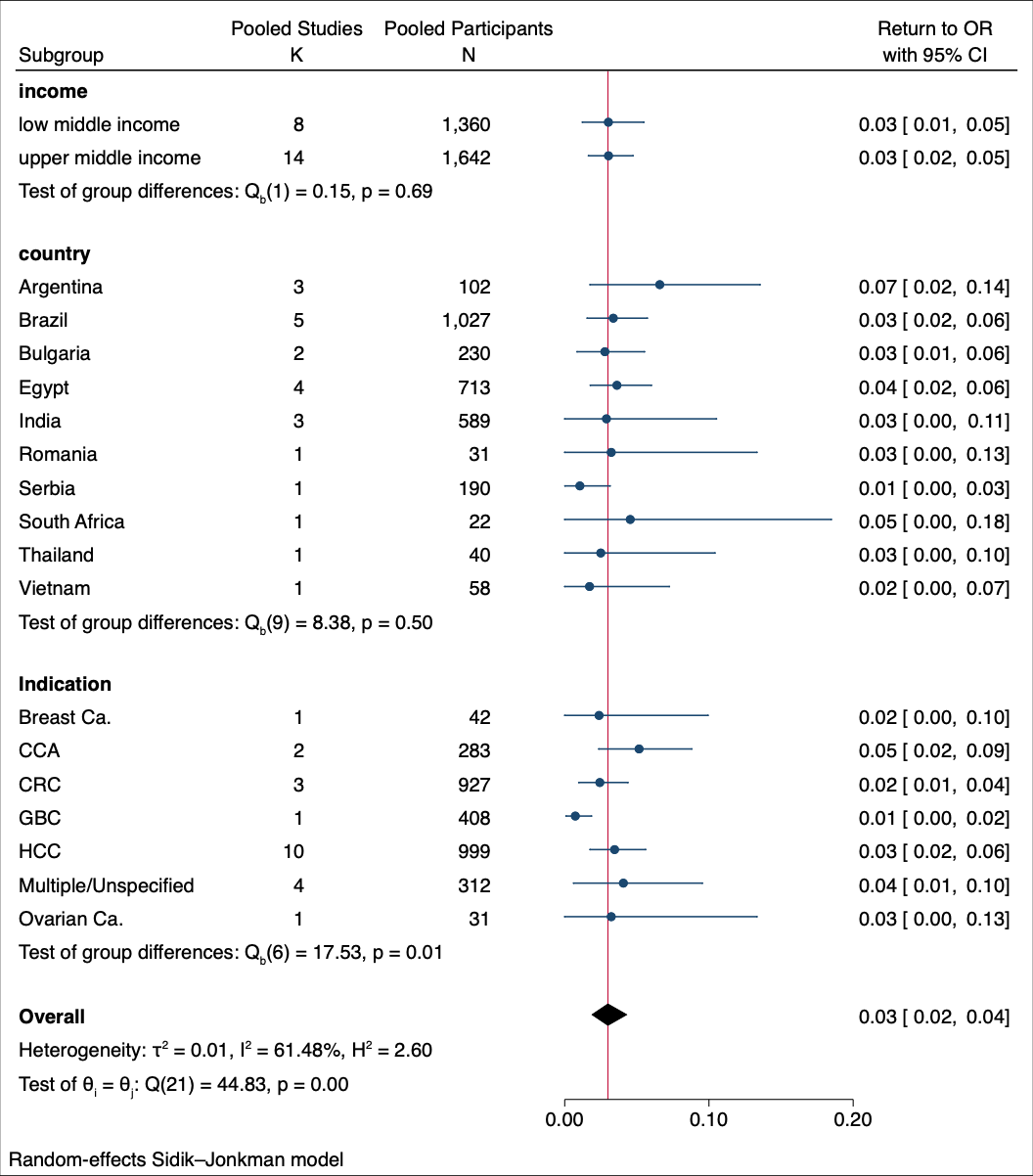


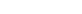


**Supplementary Forest Plot 4:** Unplanned Postoperative Intubation


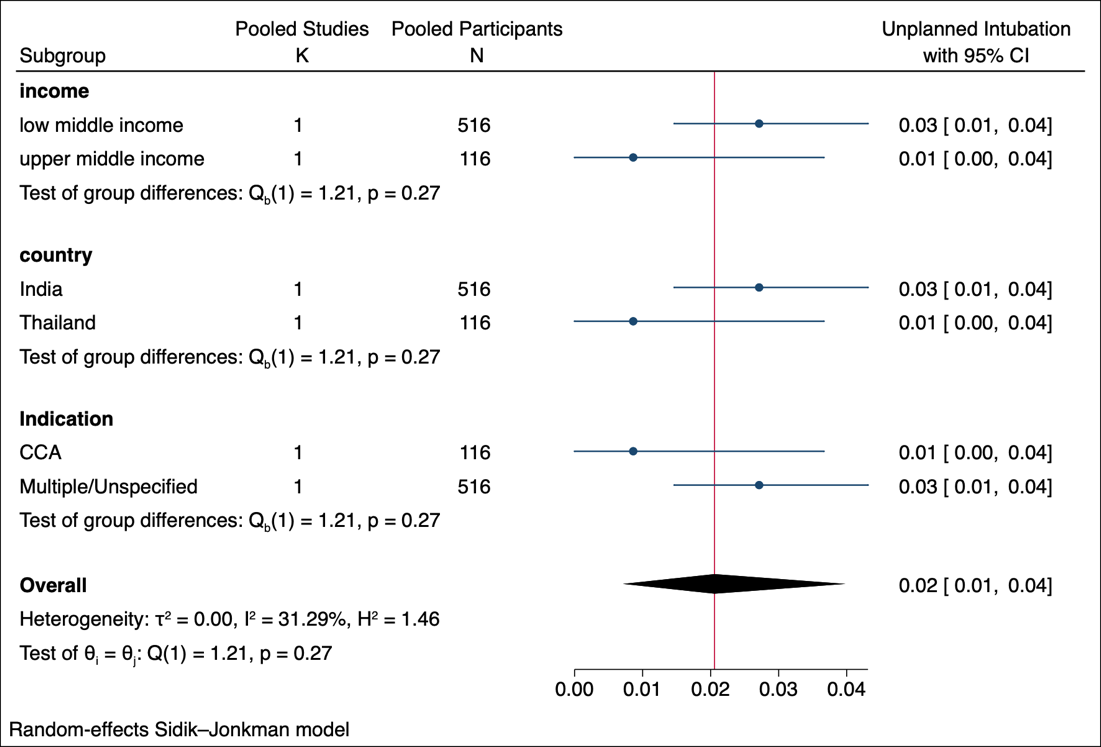


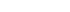


**Supplementary Forest Plot 5:** Endoscopic or Percutaneous Intervention


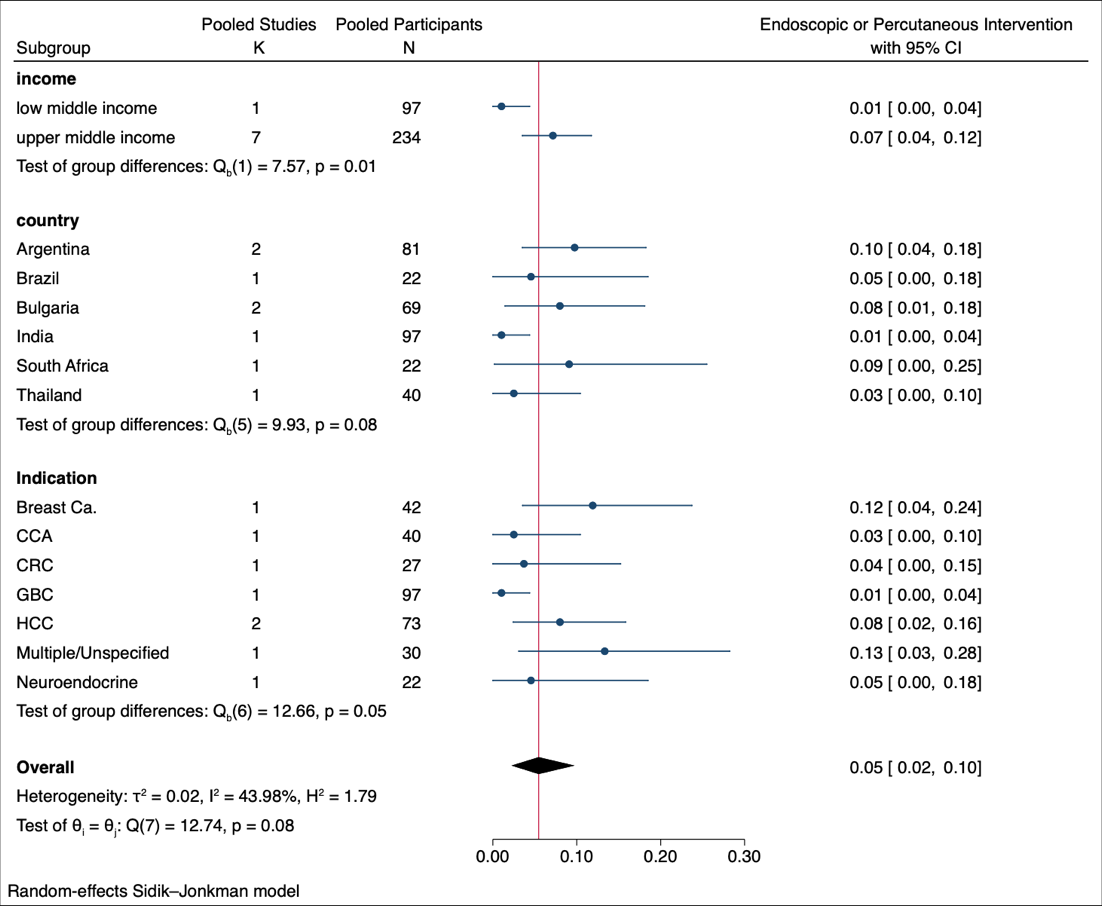


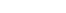


**Supplementary Forest Plot 6:** Readmission


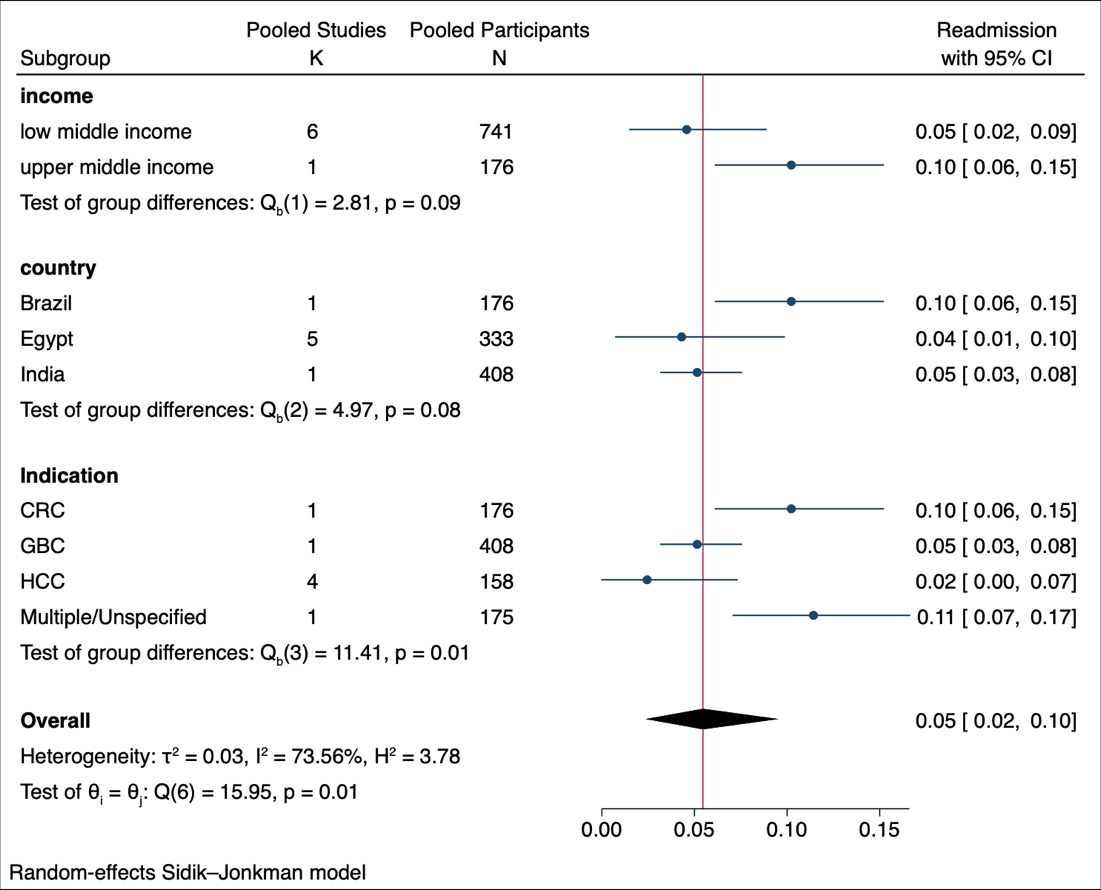


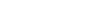


**Supplementary Forest Plot 7:** Length of Stay


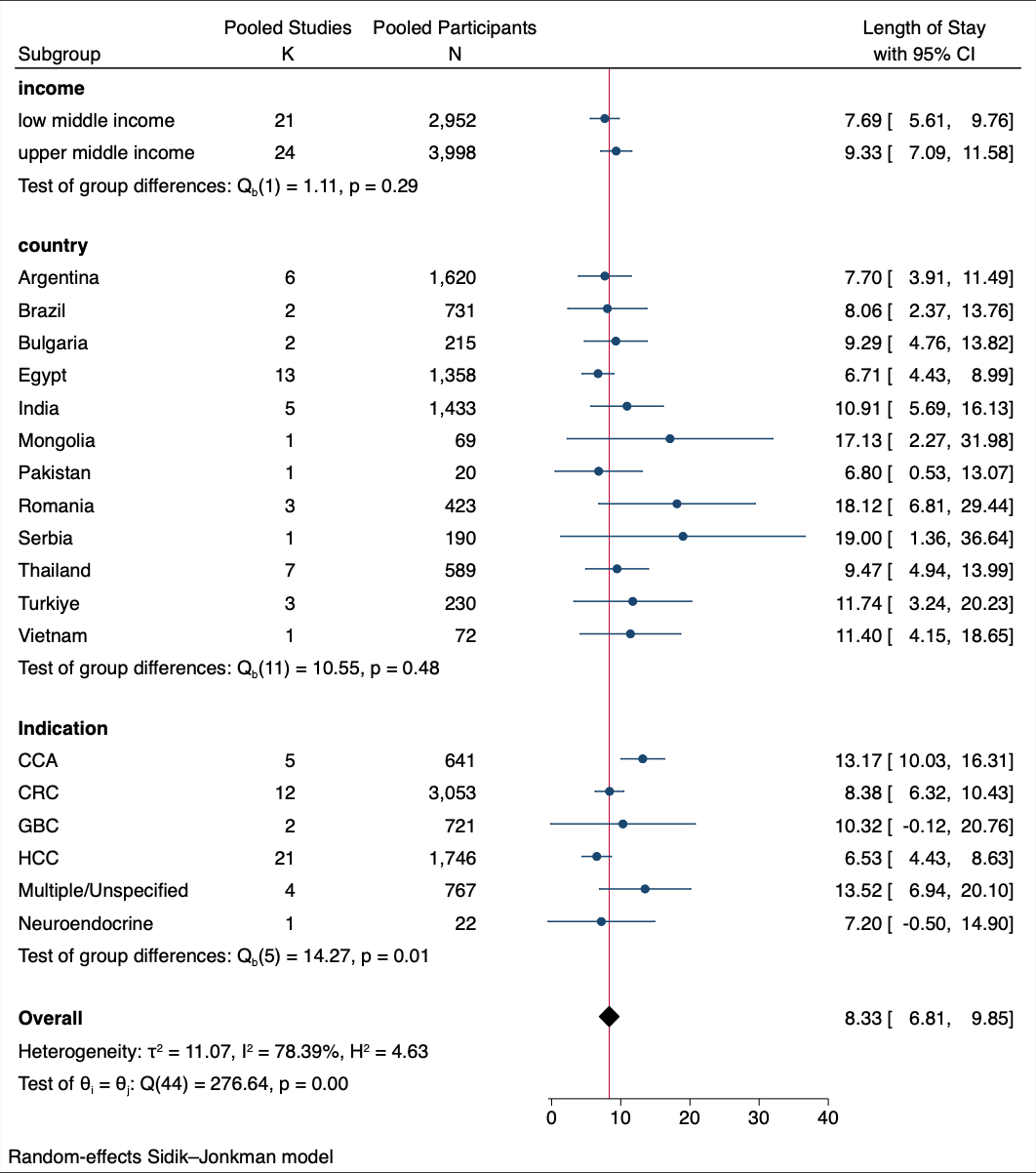


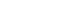

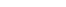


**Supplementary Forest Plot 8:** In-Hospital Morbidity


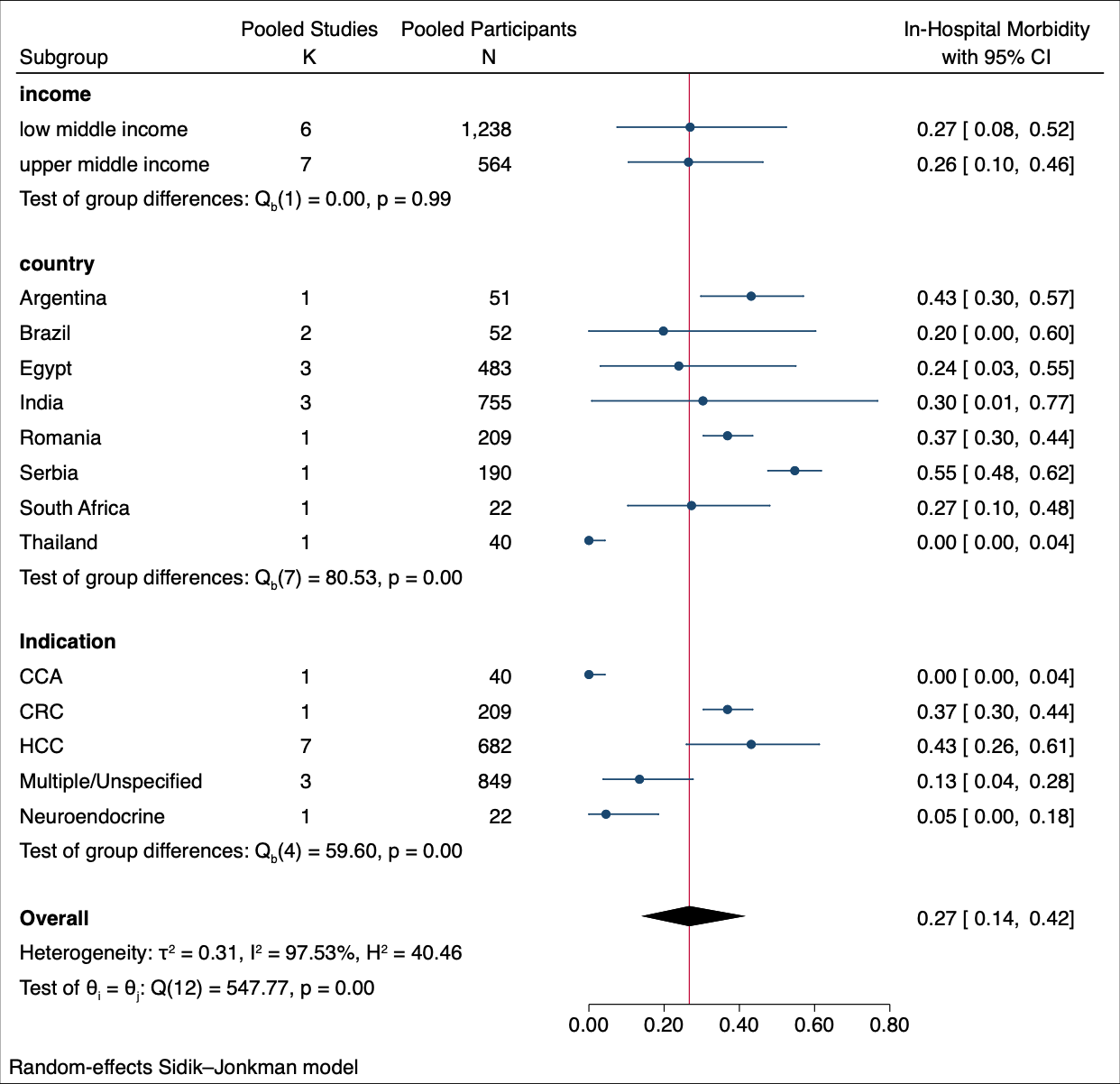


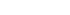


**Supplementary Forest Plot 9:** 30-Day Morbidity


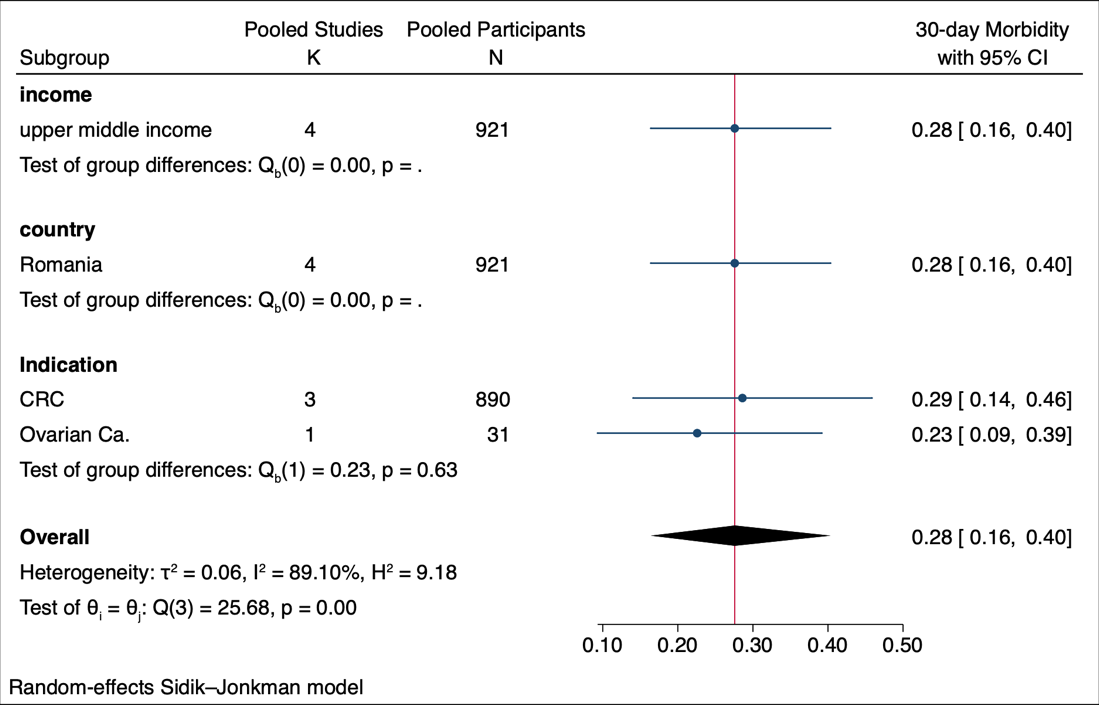


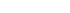


**Supplementary Forest Plot 10:** 90-Day Morbidity


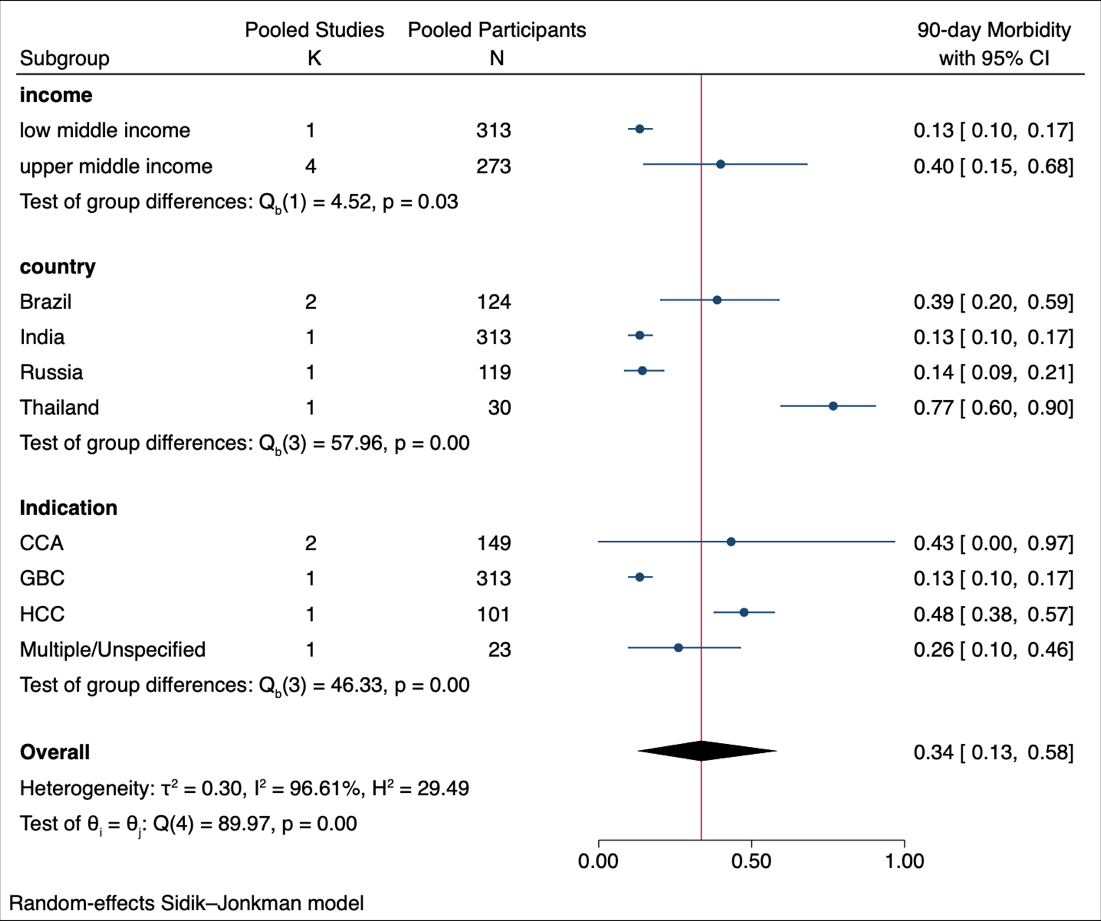


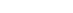

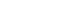


**Supplementary Forest Plot 11:** Clavien-Dindo Grade 1 or 2


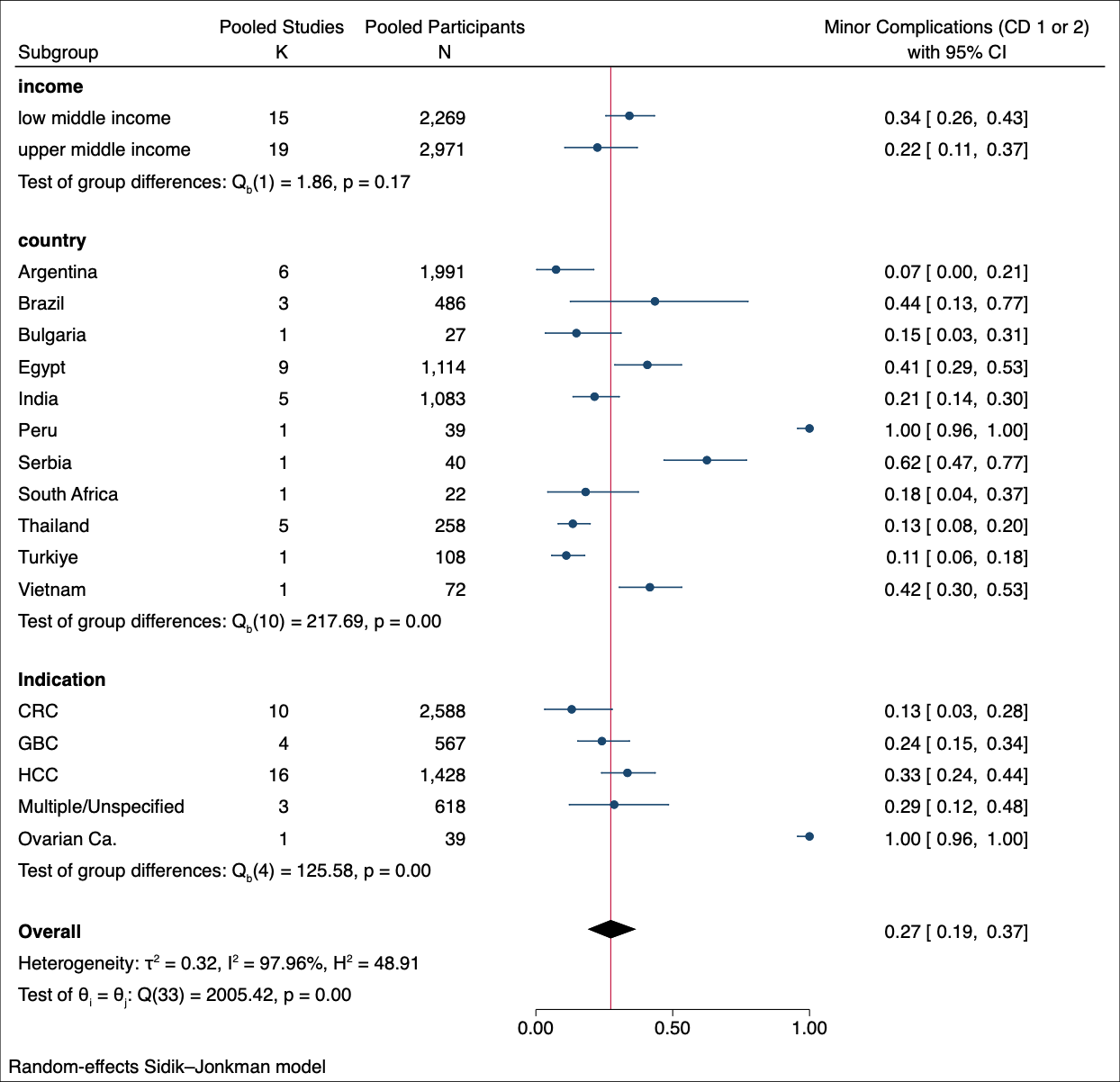


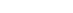


**Supplementary Forest Plot 12:** Clavien-Dindo Grade ≥3


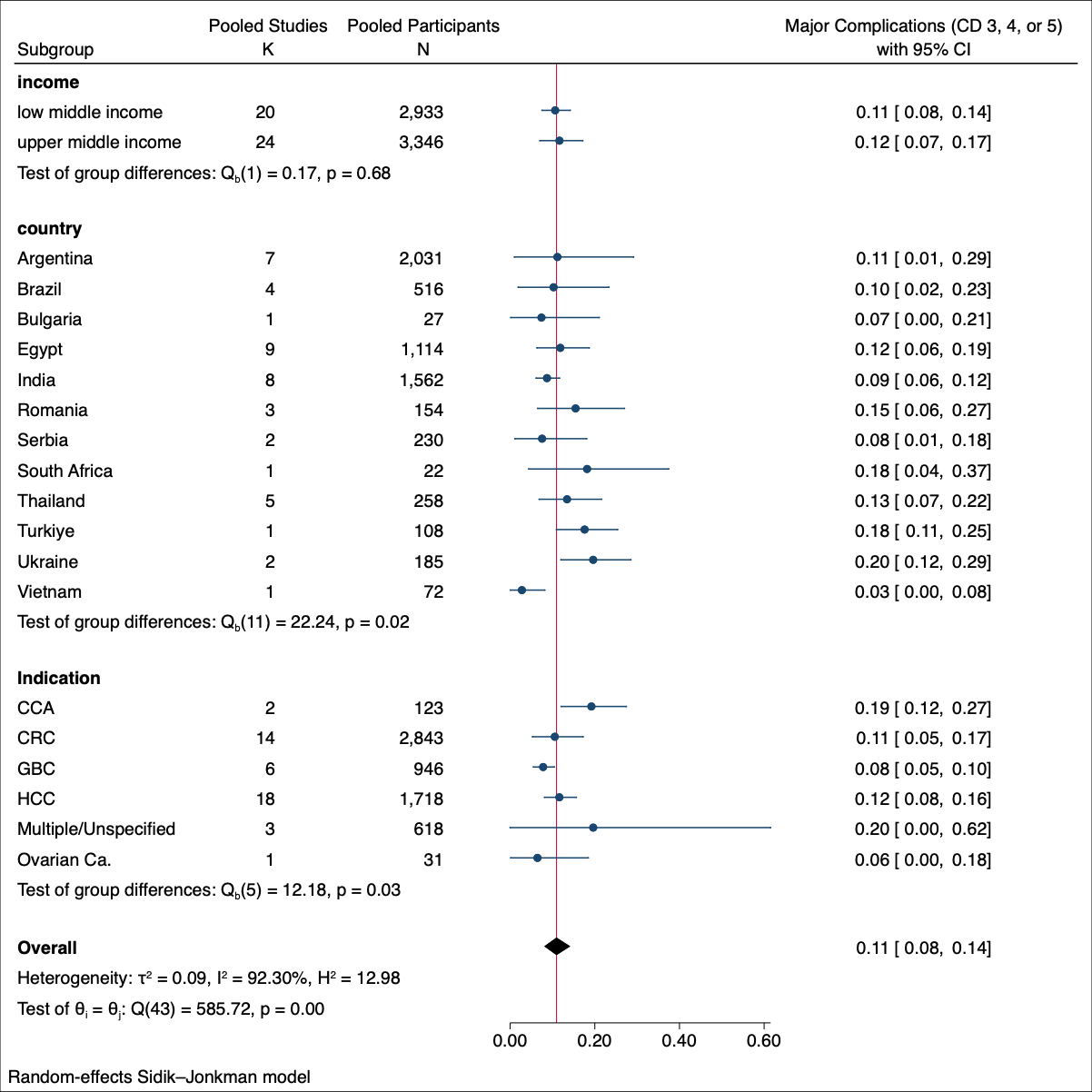


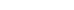

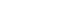


**Supplementary Forest Plot 13:** In-Hospital Mortality


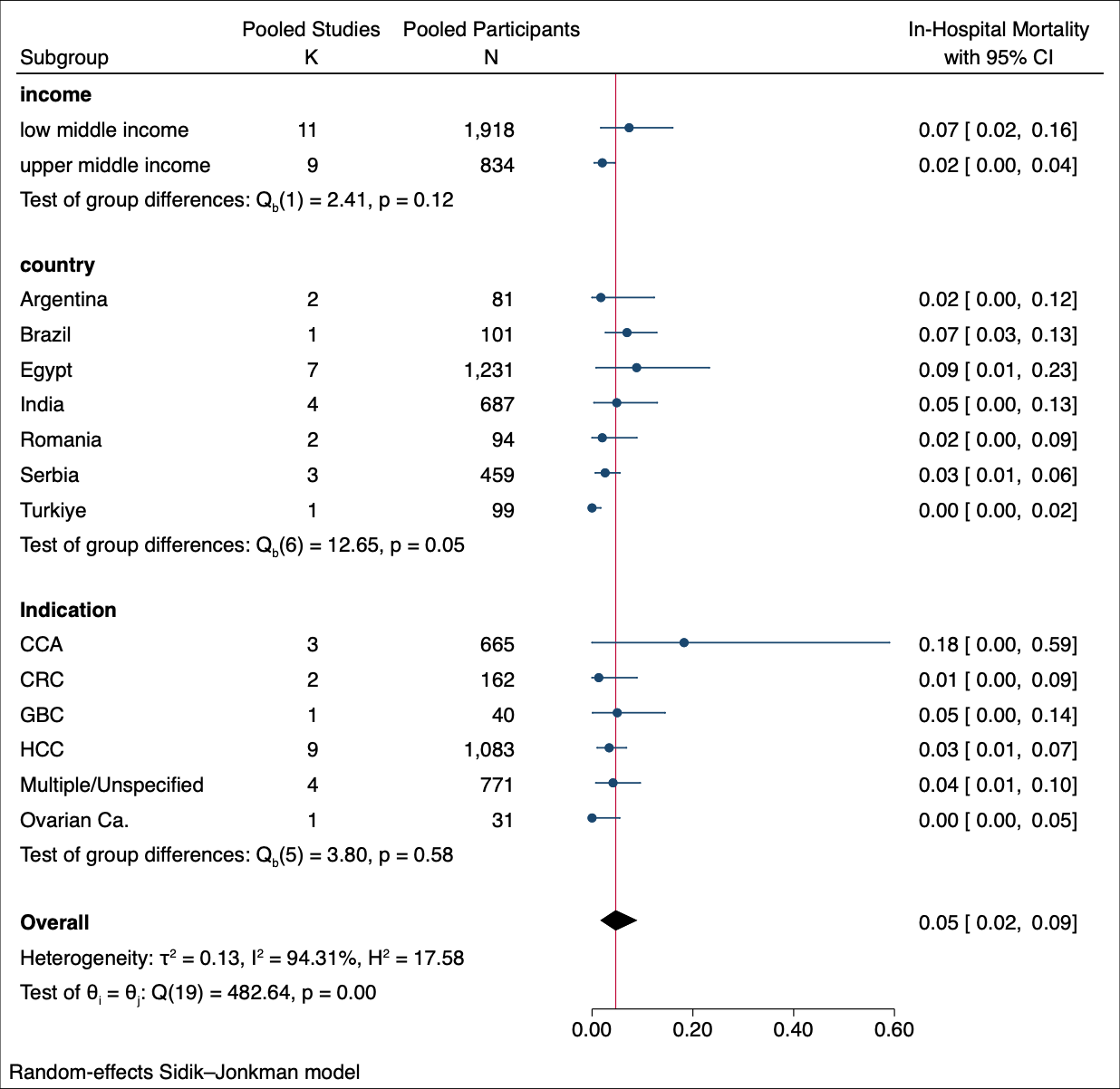


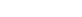


**Supplementary Forest Plot 14:** 30-Day Mortality


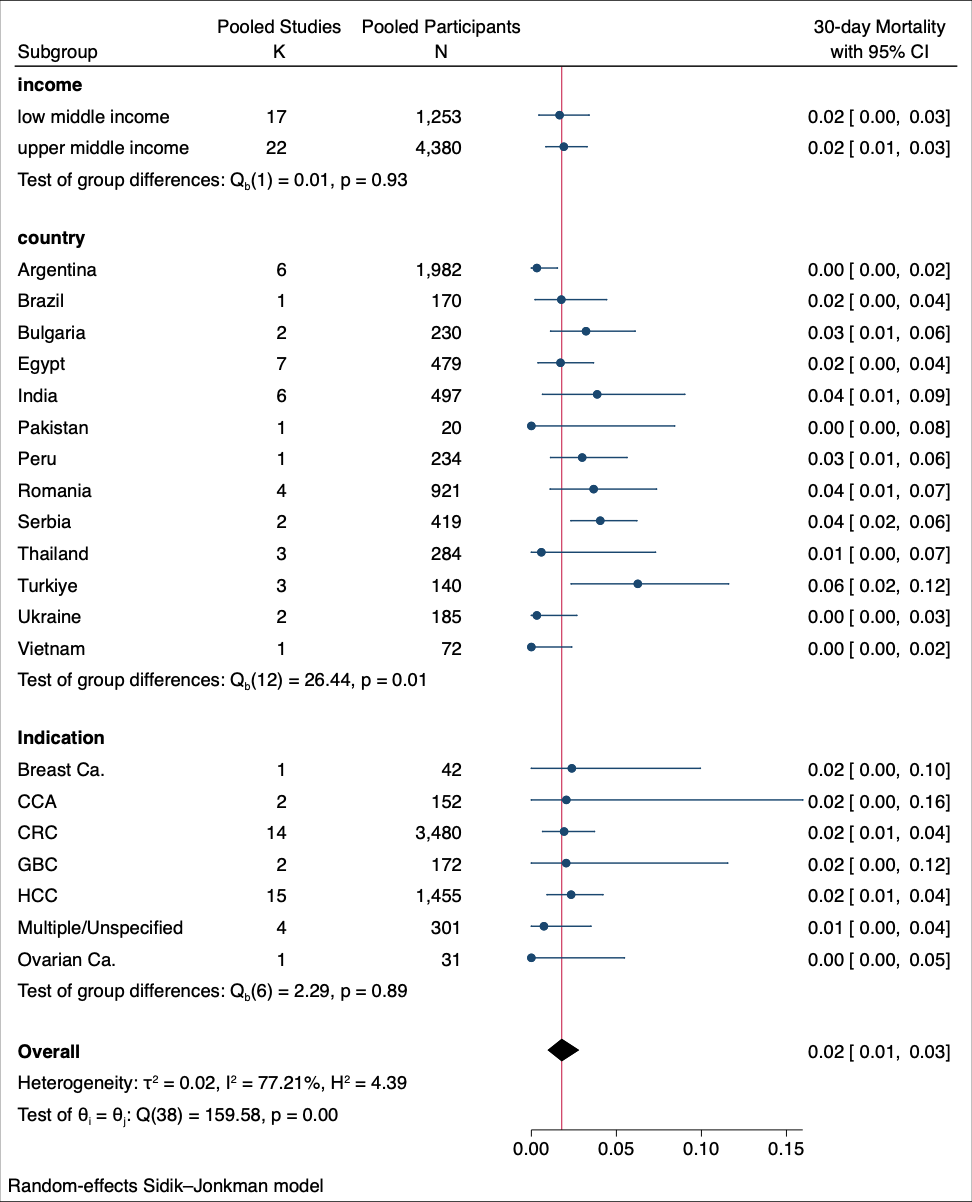


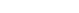


**Supplementary Forest Plot 15:** 90-Day Mortality


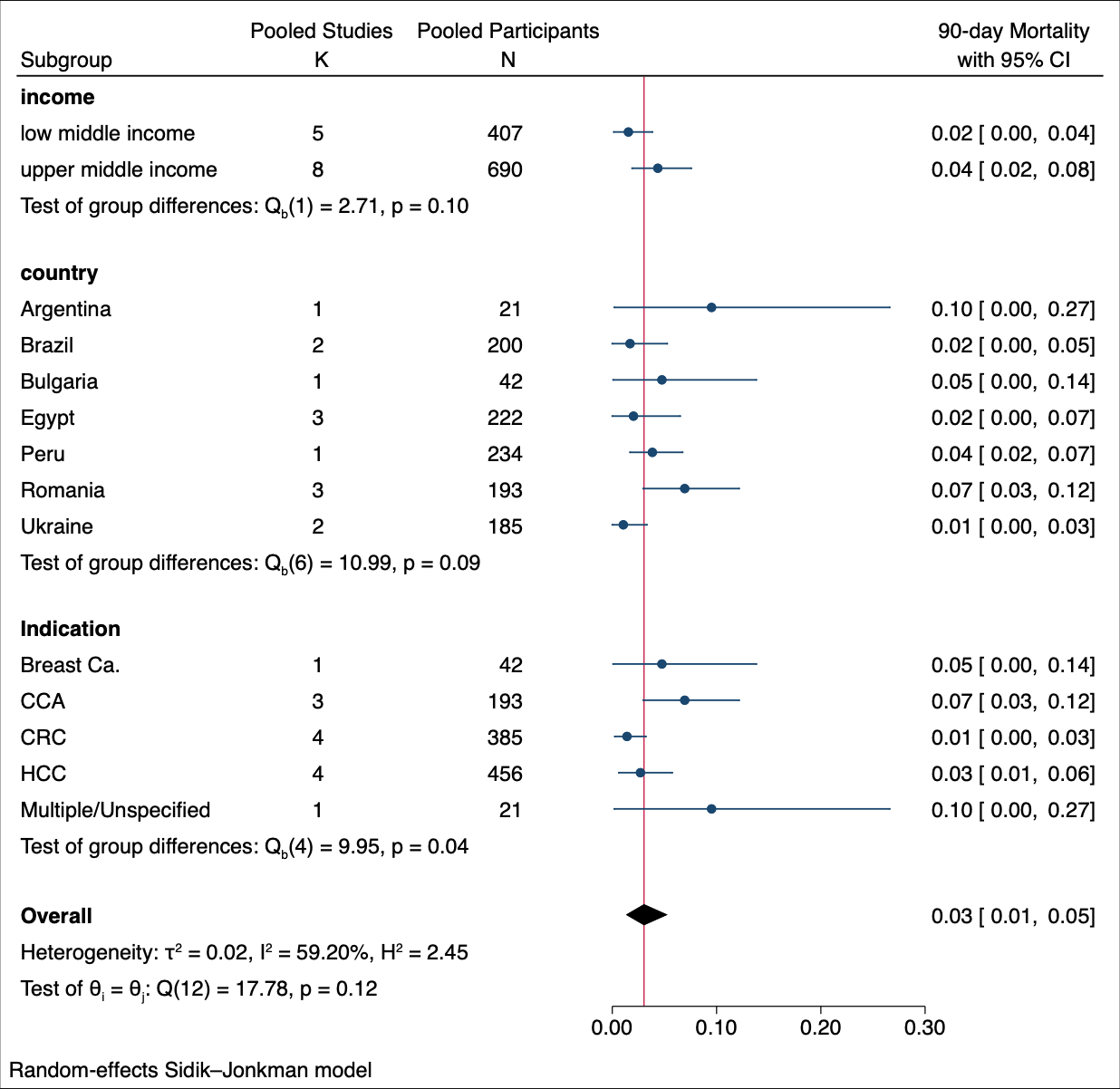


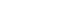

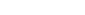


**Supplementary Forest Plot 16:** Liver Failure or Dysfunction


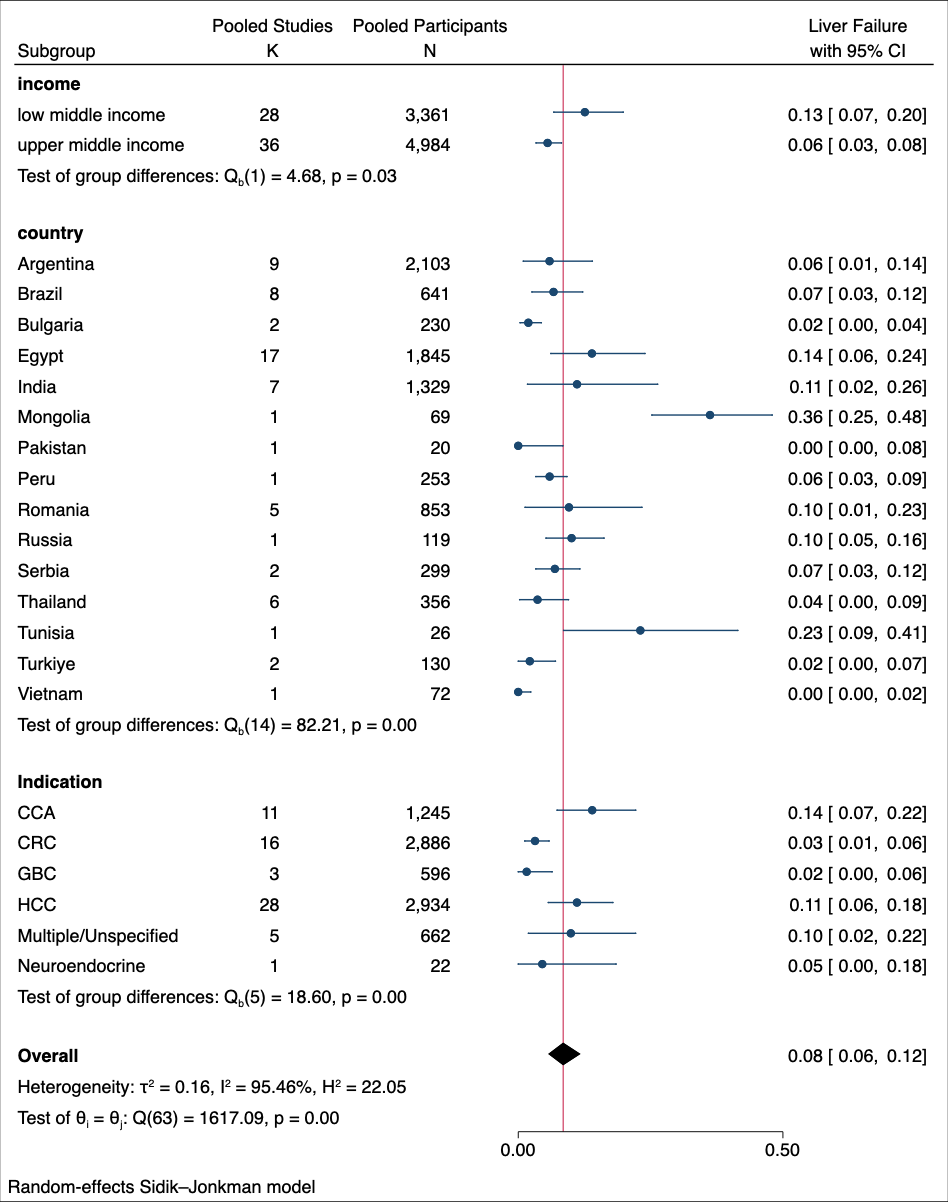


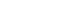


**Supplementary Forest Plot 17:** Bile Leaks


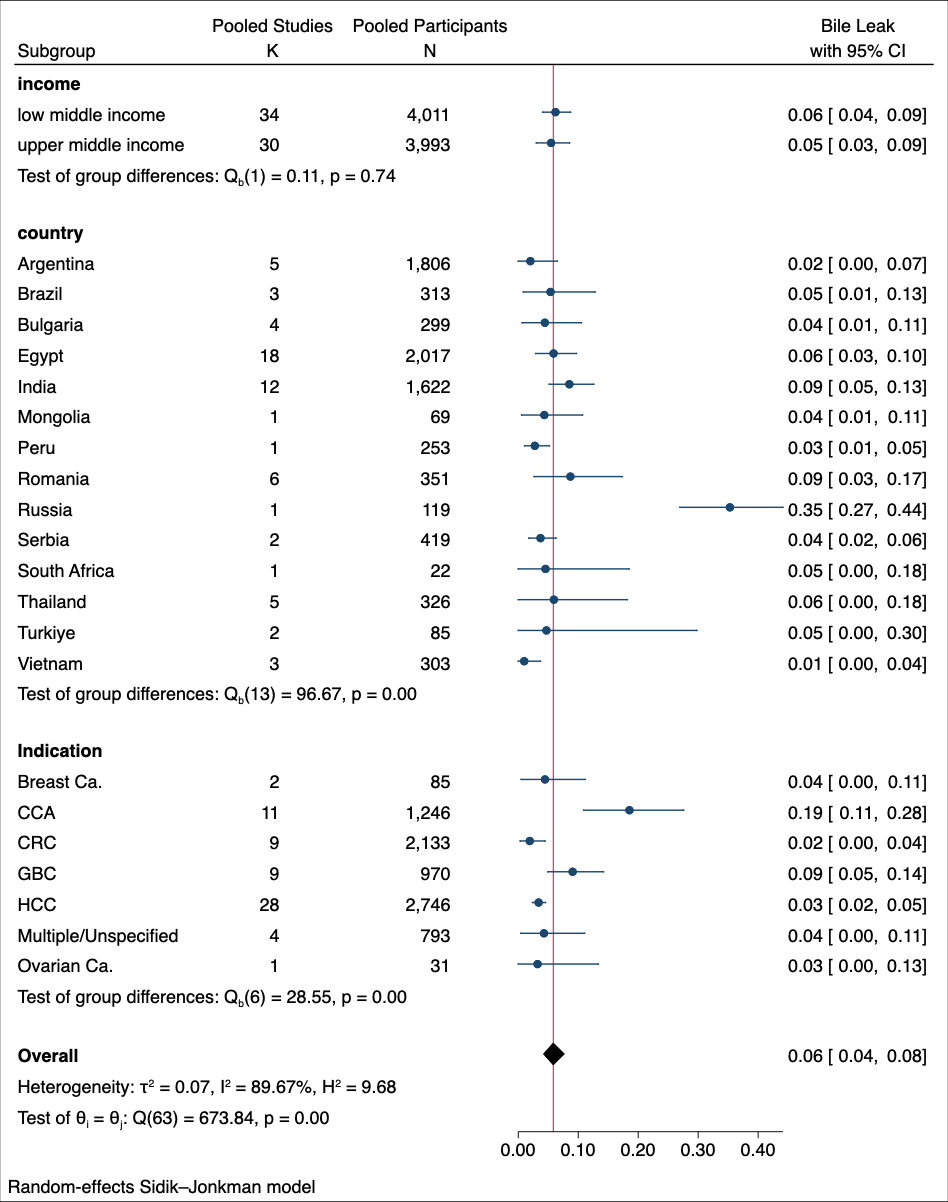


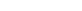


**Supplementary Forest Plot 18:** Hemorrhage


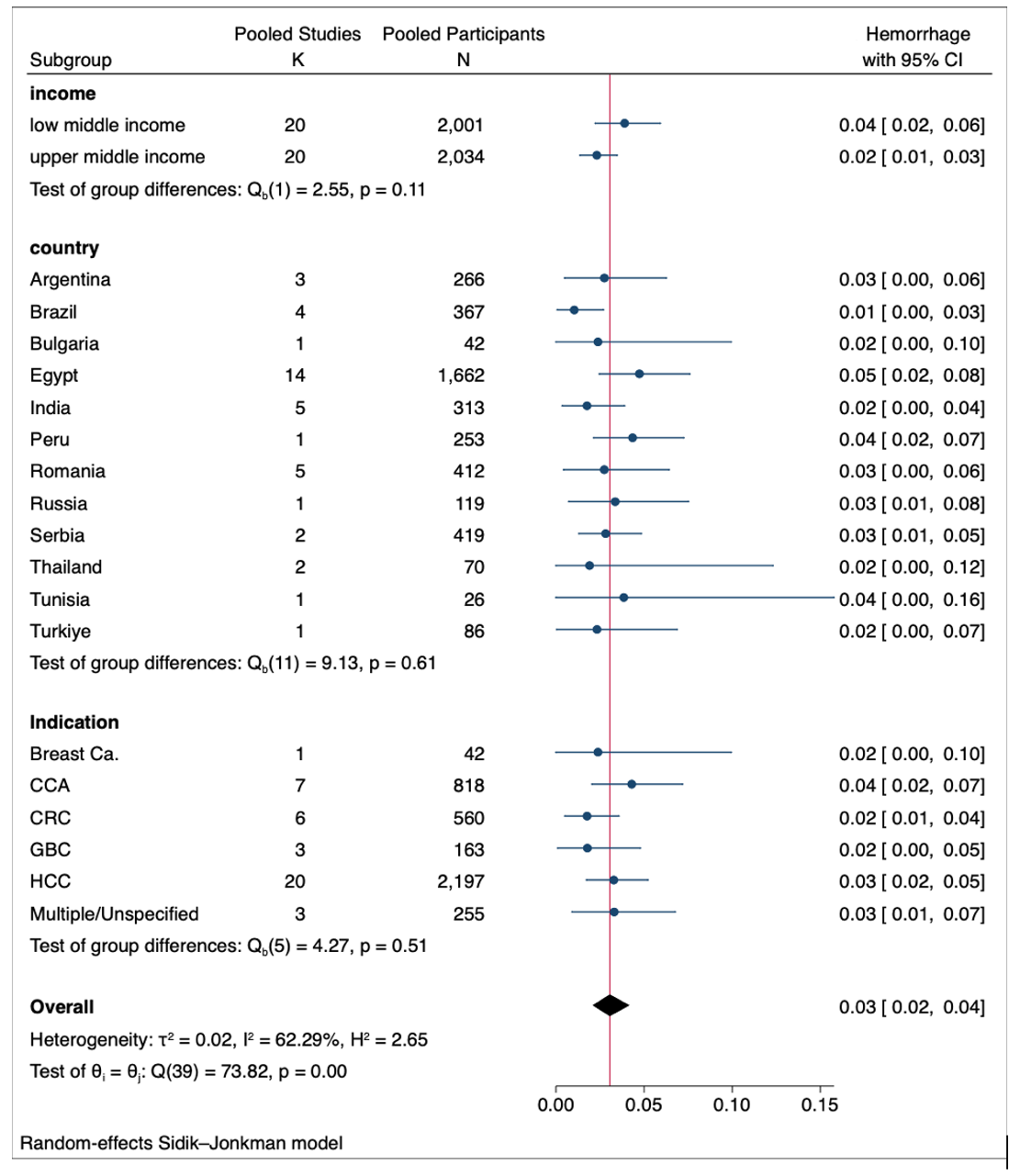


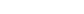


**Supplementary Forest Plot 19:** Myocardial Infarction


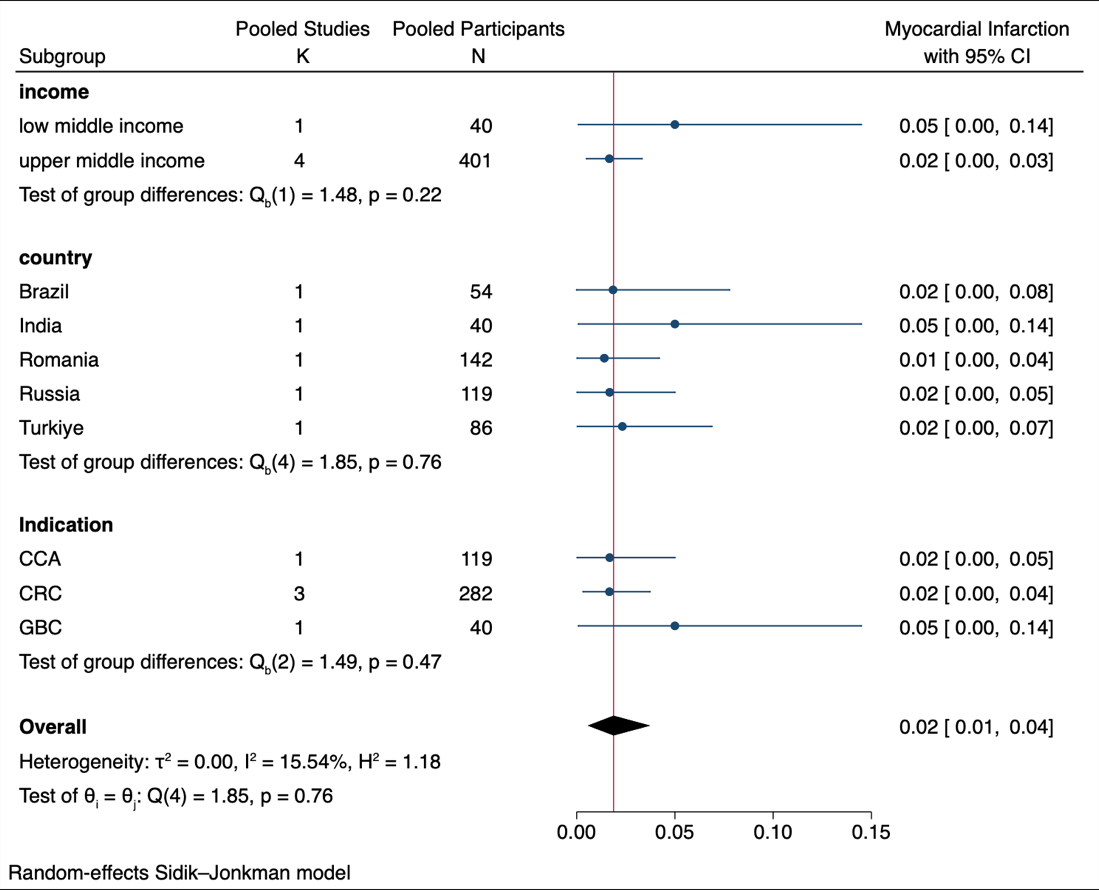


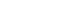


**Supplementary Forest Plot 20:** Thrombotic or Thromboembolic Events


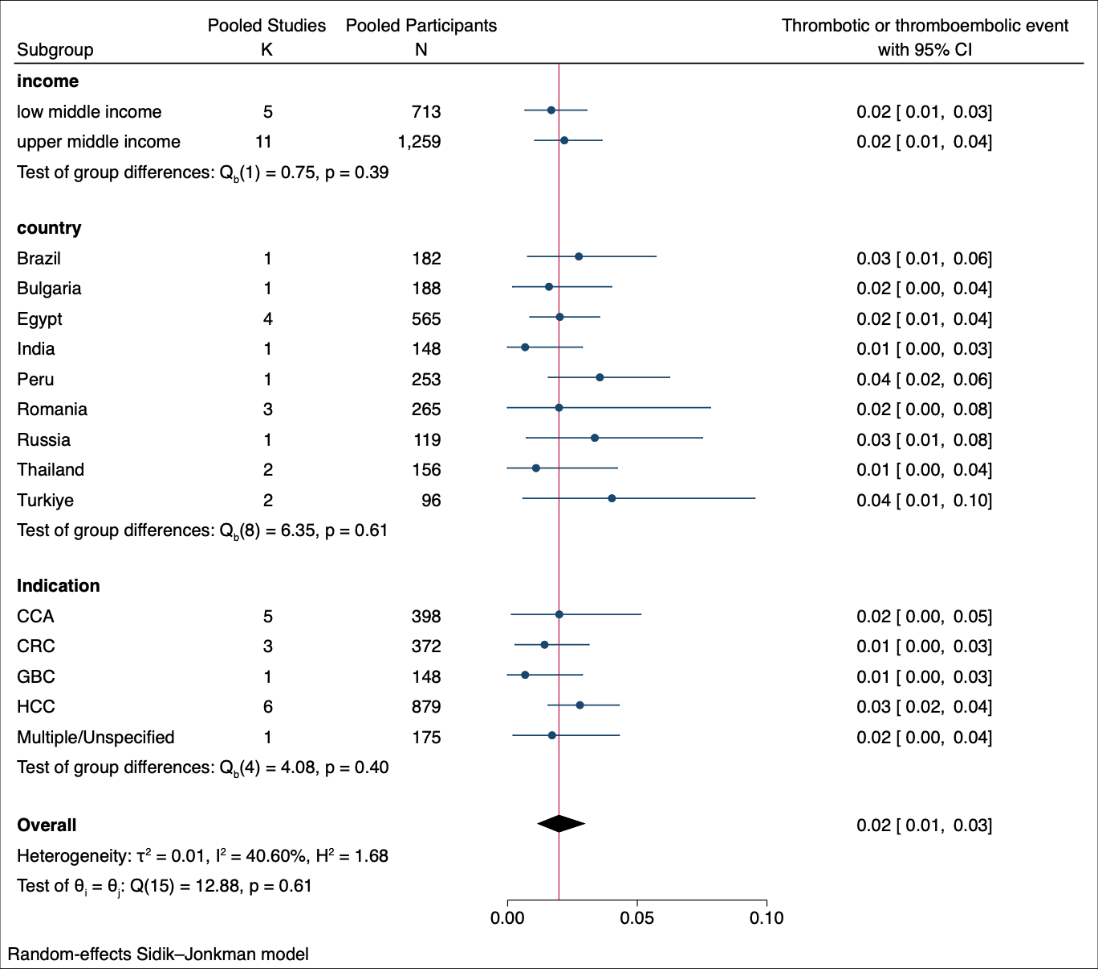


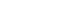


**Supplementary Forest Plot 21:** Cerebrovascular Accidents


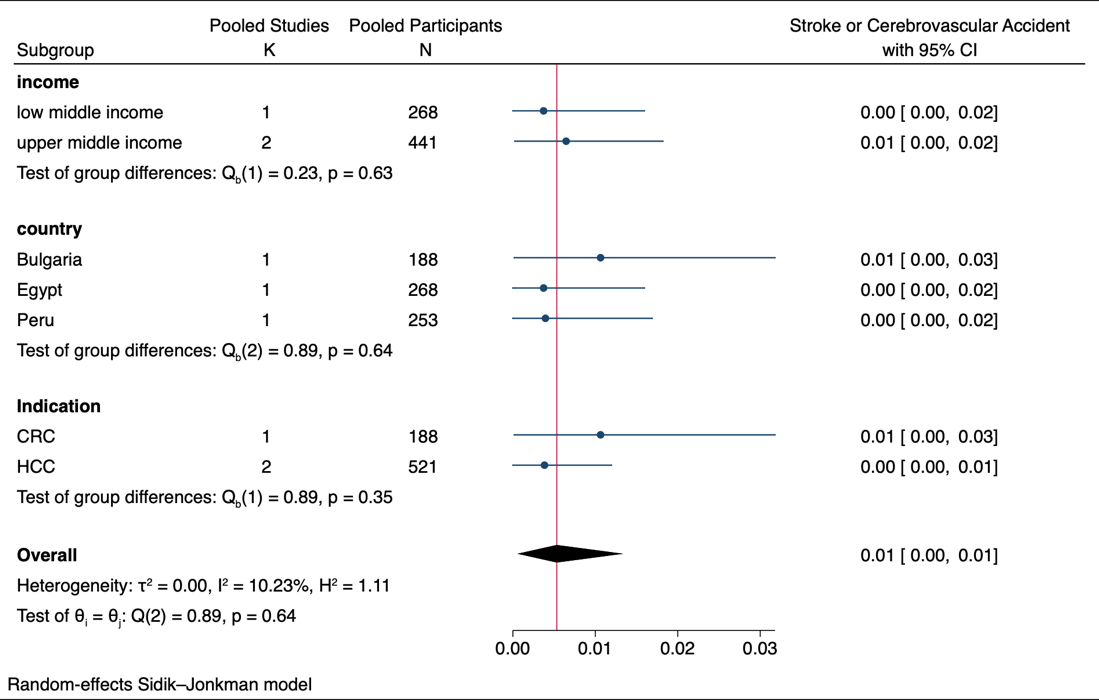


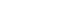


**Supplementary Forest Plot 22:** Pneumonia


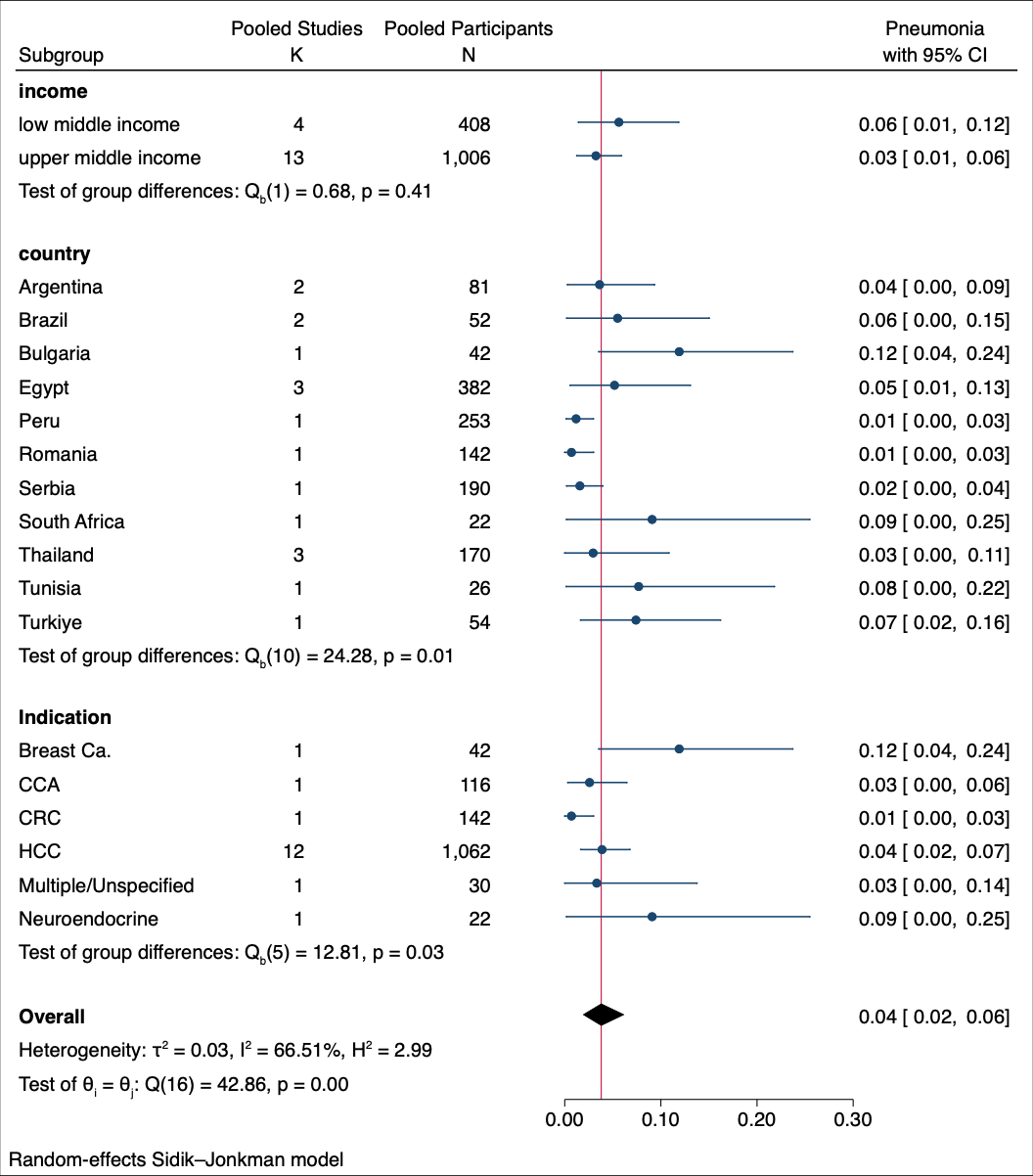


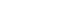


**Supplementary Forest Plot 23:** Liver Abscess


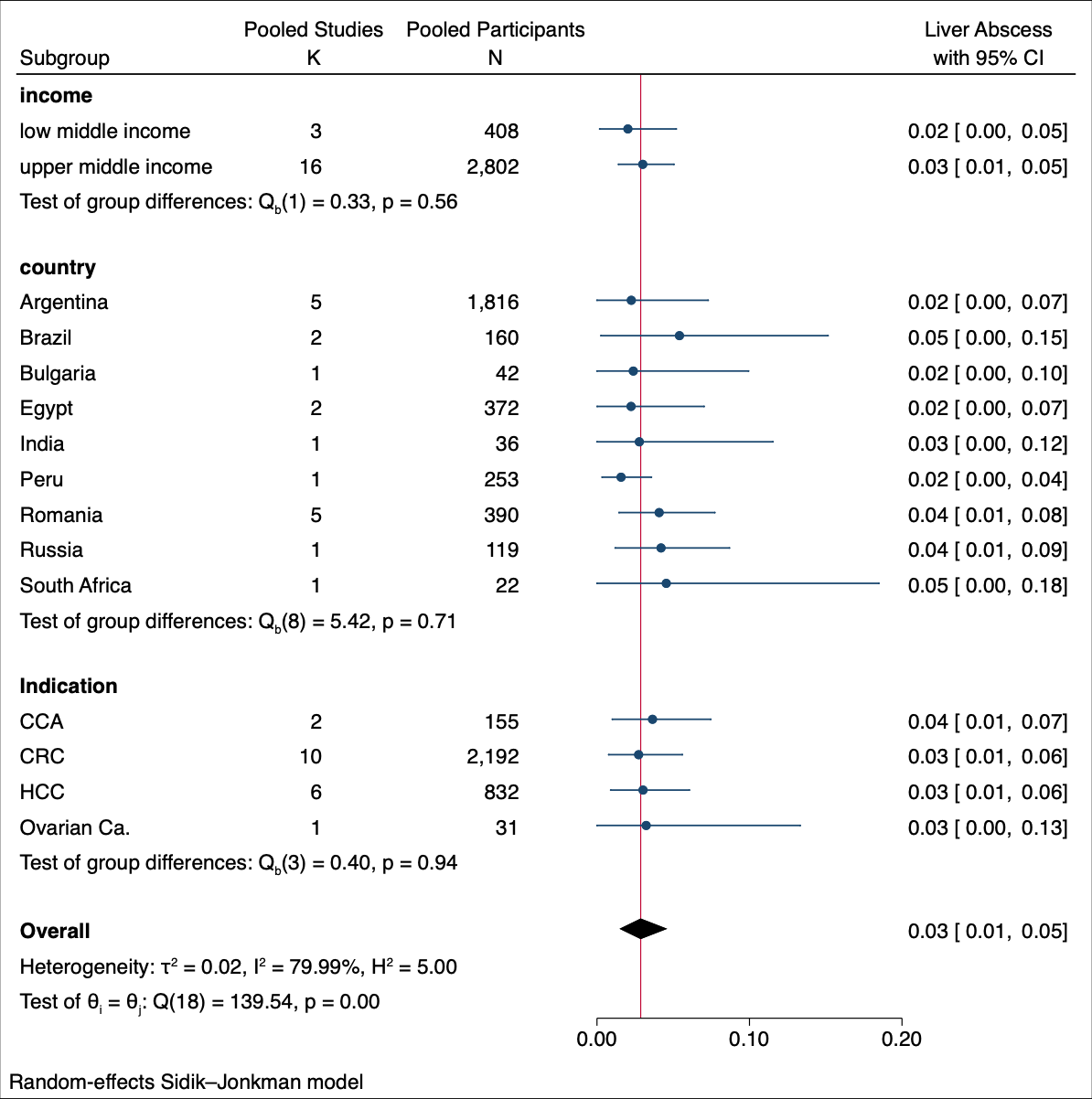


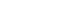


**Supplementary Forest Plot 24:** Wound Dehiscence or Infection


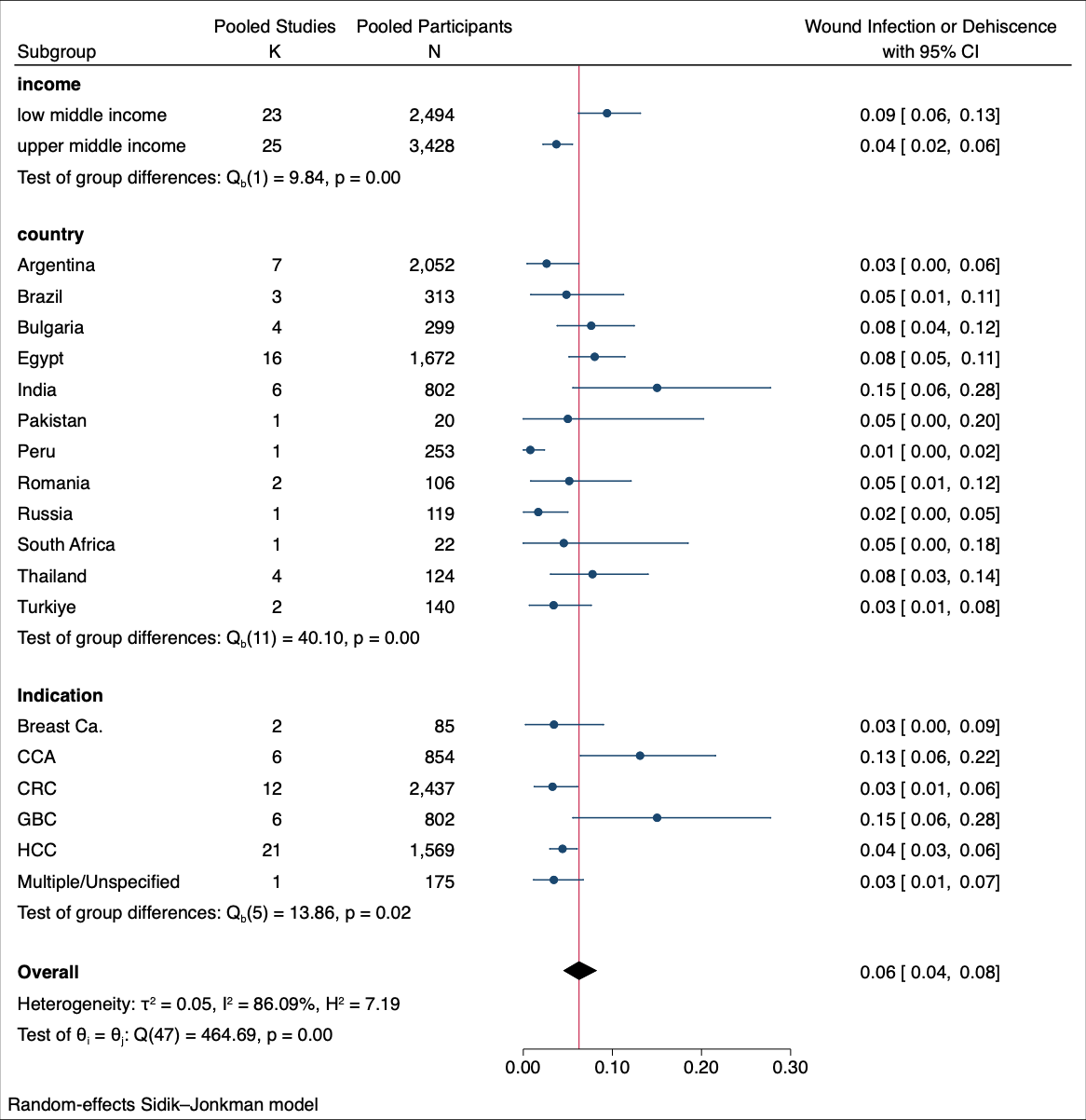


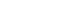


**Supplementary Forest Plot 25:** Urinary Tract Infection


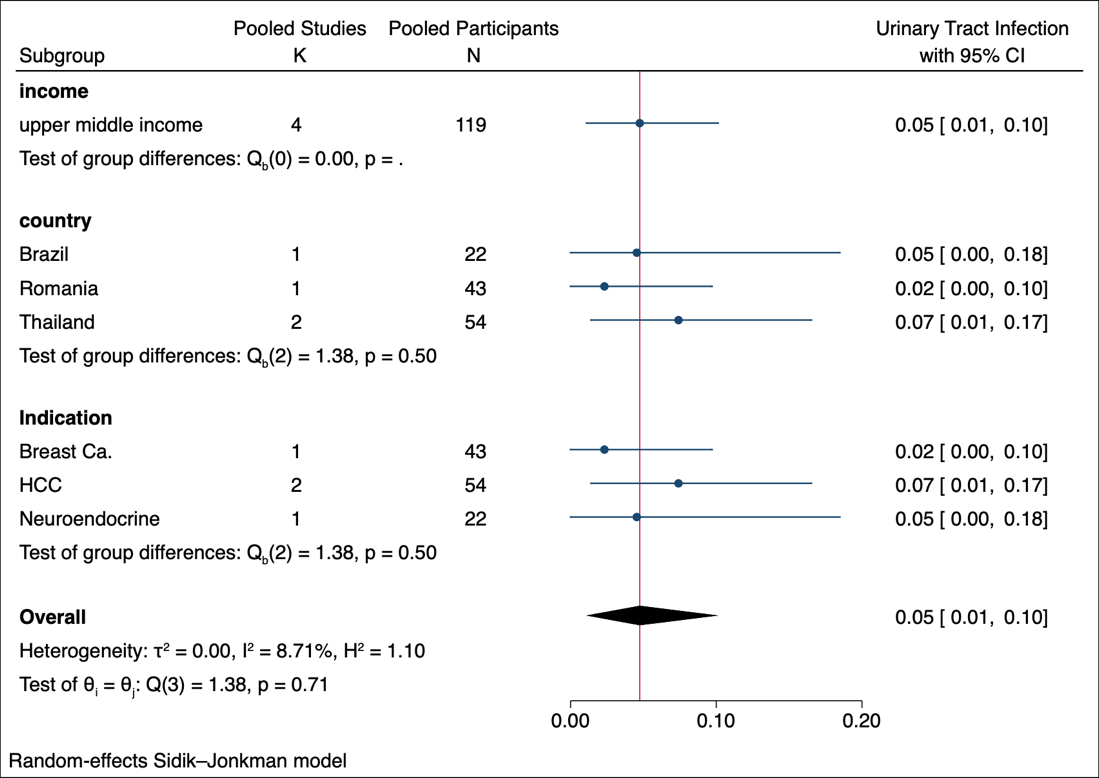


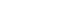


**Supplementary Forest Plot 26:** Bacteremia


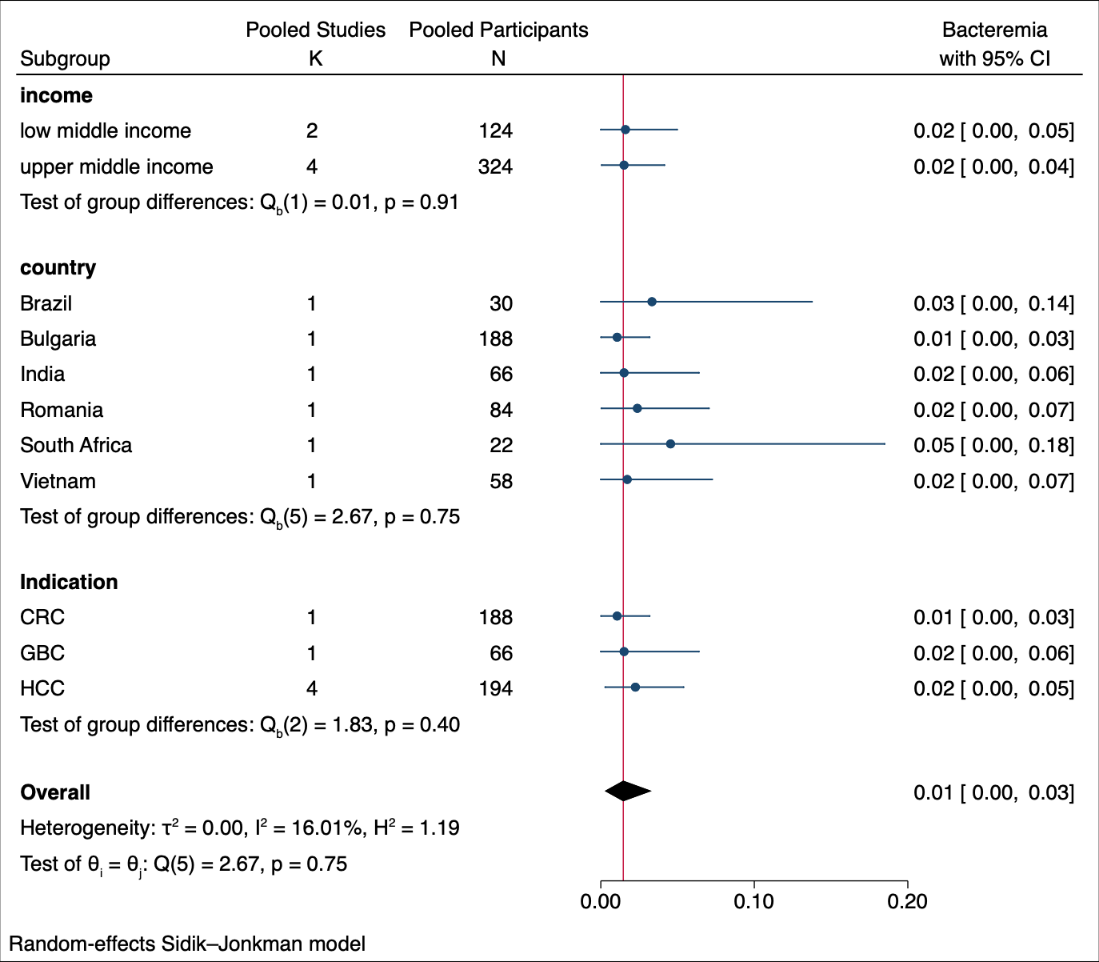


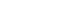


**Supplementary Forest Plot 27:** Other Infections


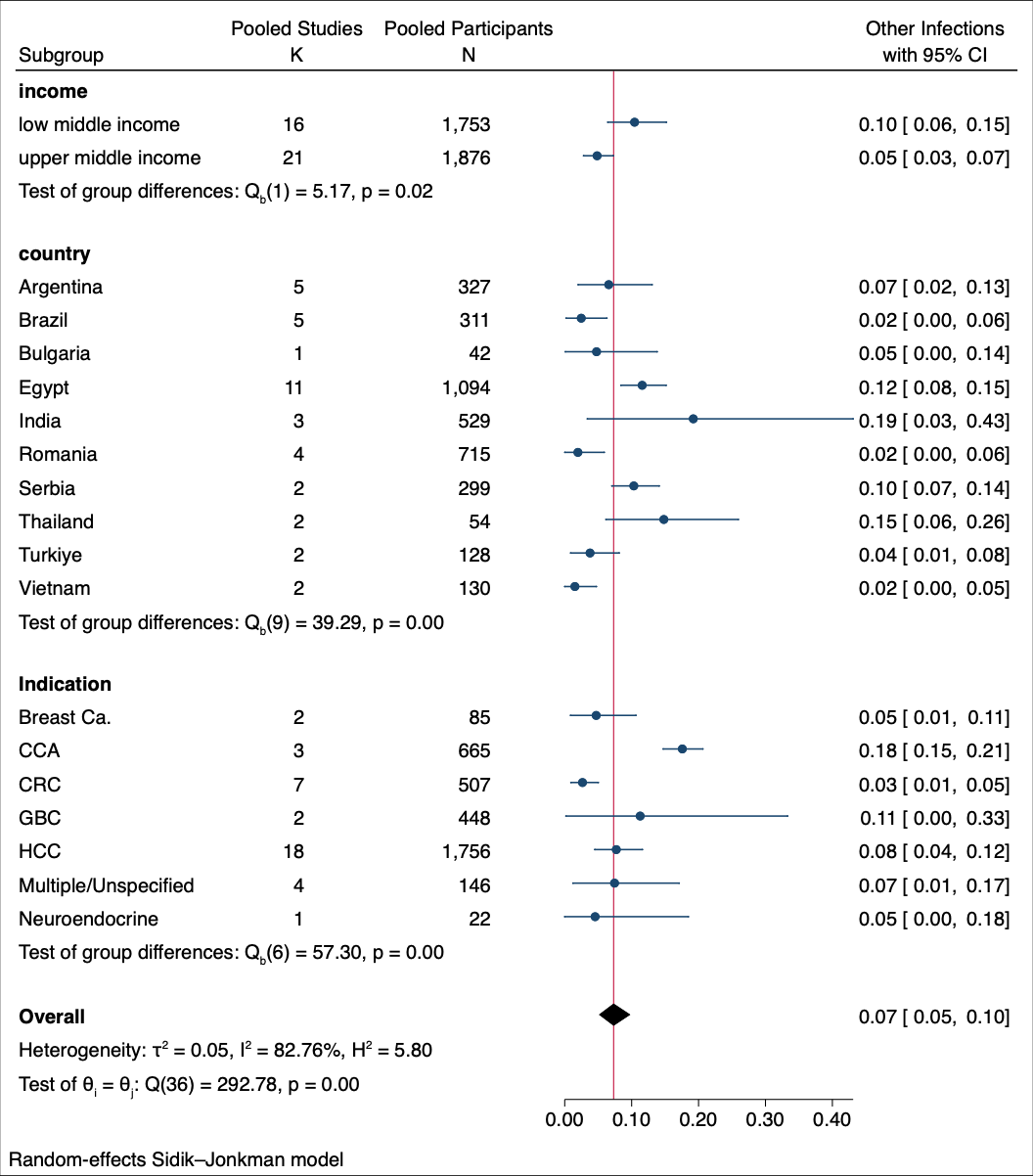


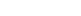


**Subsection C: Funnel Plots**

**Supplementary Funnel Plot 1:** Operative Duration


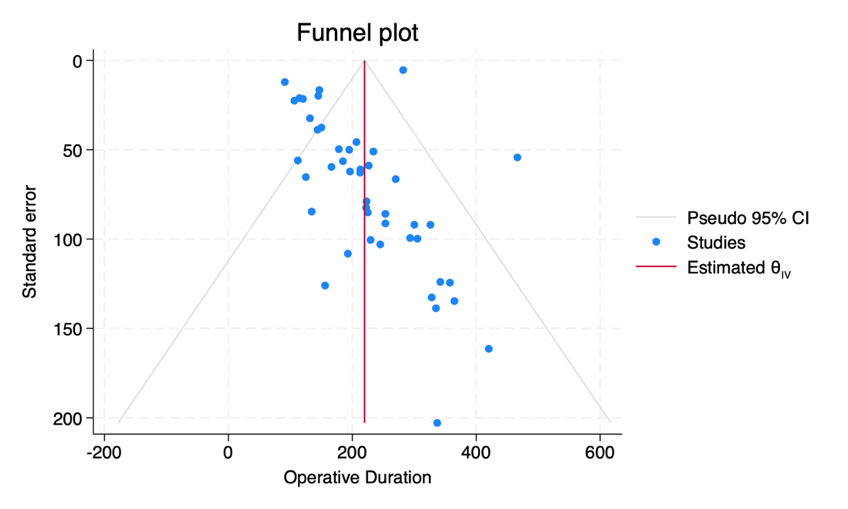


**Supplementary Funnel Plot 2:** Margin Status


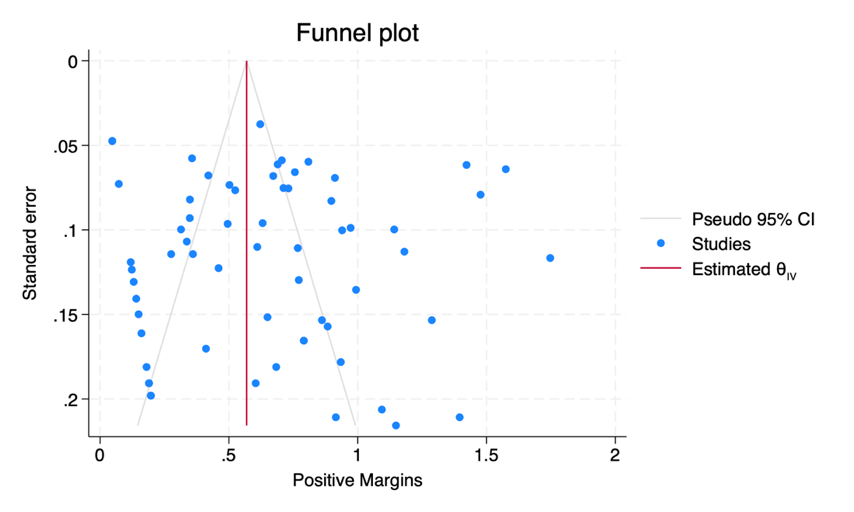


**Supplementary Funnel Plot 3:** Return to Operating Room


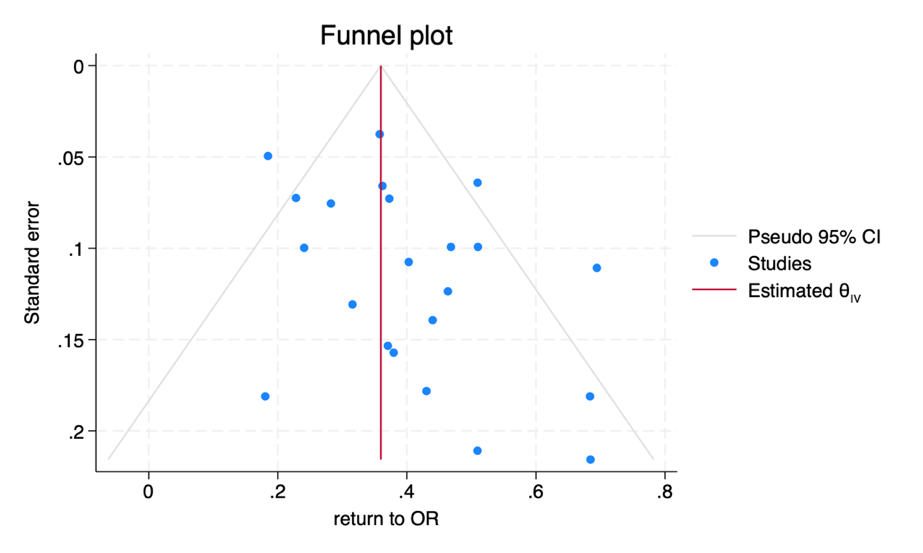


**Supplementary Funnel Plot 4:** Unplanned Postoperative Intubation


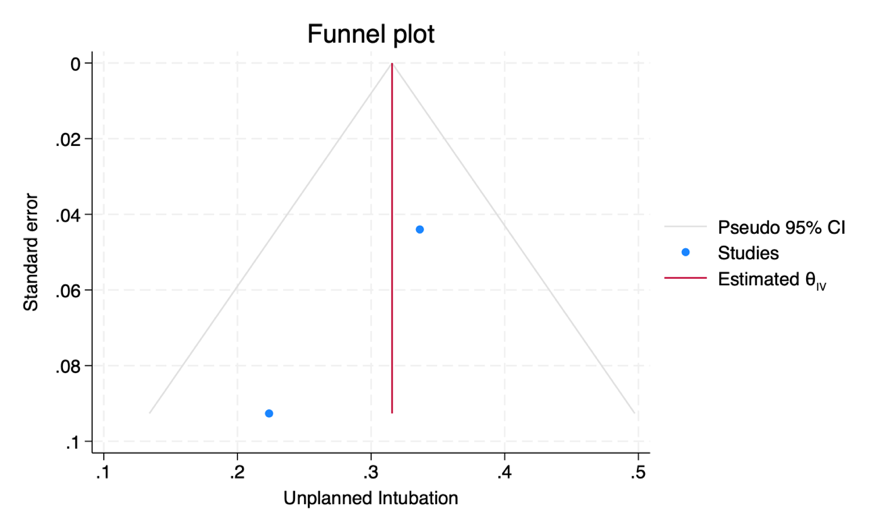


**Supplementary Funnel Plot 5:** Endoscopic or Percutaneous Intervention


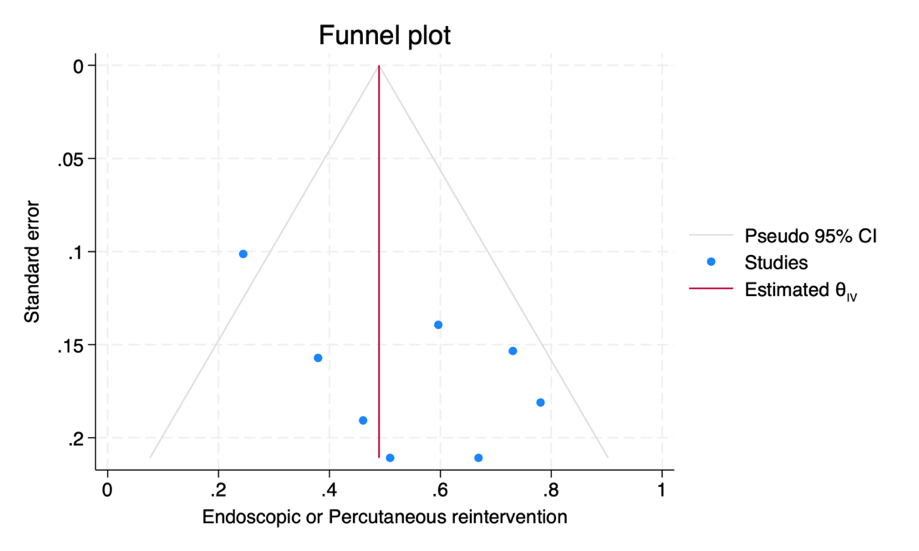


**Supplementary Funnel Plot 6:** Readmission


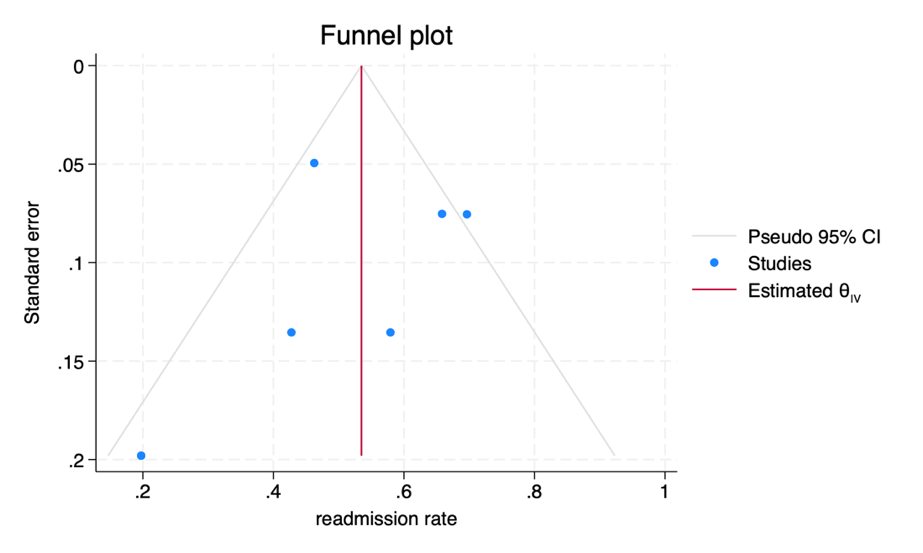


**Supplementary Funnel Plot 7:** Length of Stay


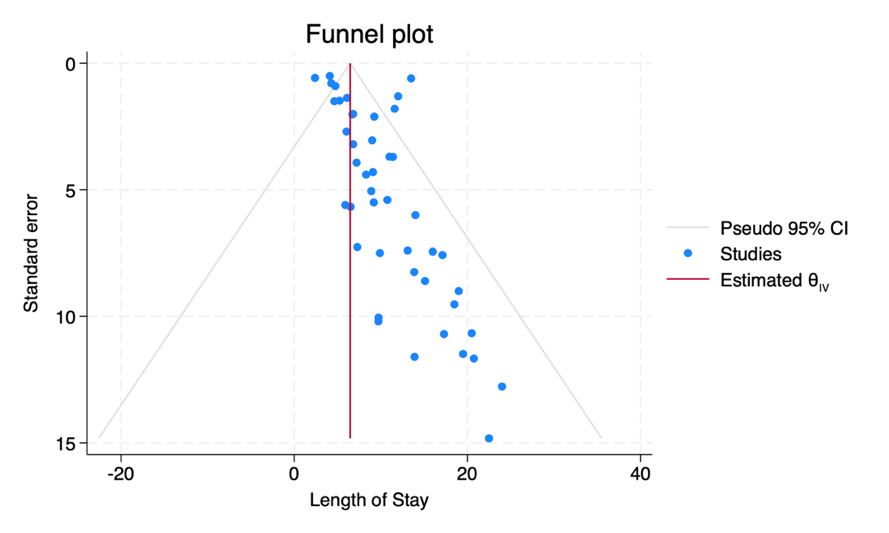


**Supplementary Funnel Plot 8:** In-Hospital Morbidity


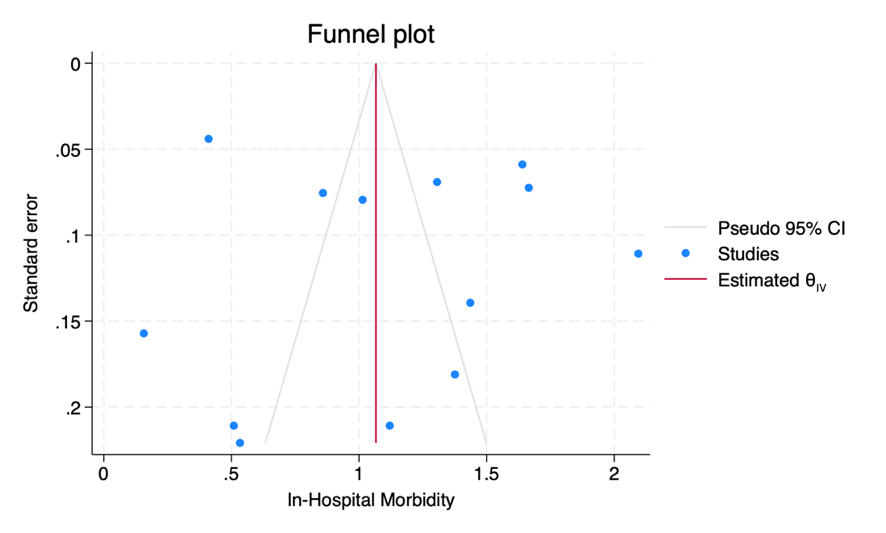


**Supplementary Funnel Plot 9:** 30-Day Morbidity


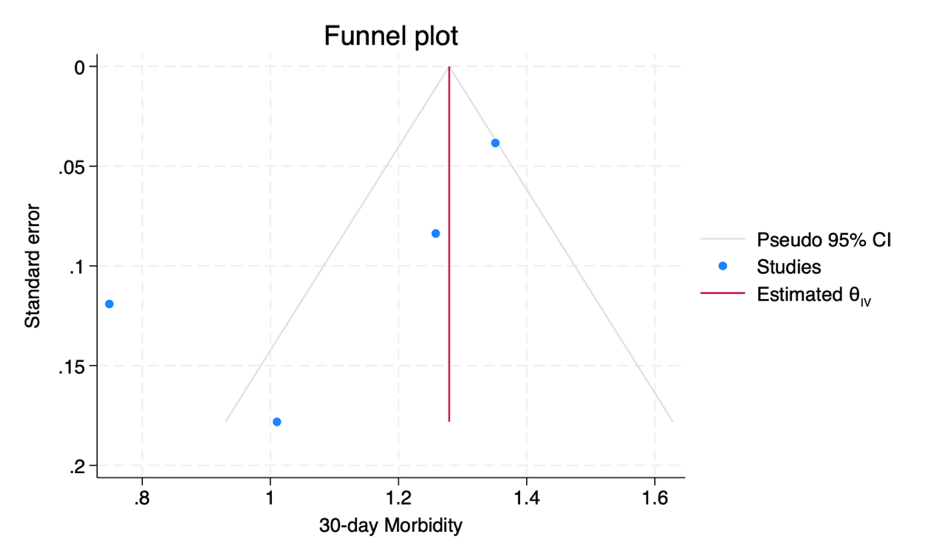


**Supplementary Funnel Plot 10:** 90-Day Morbidity


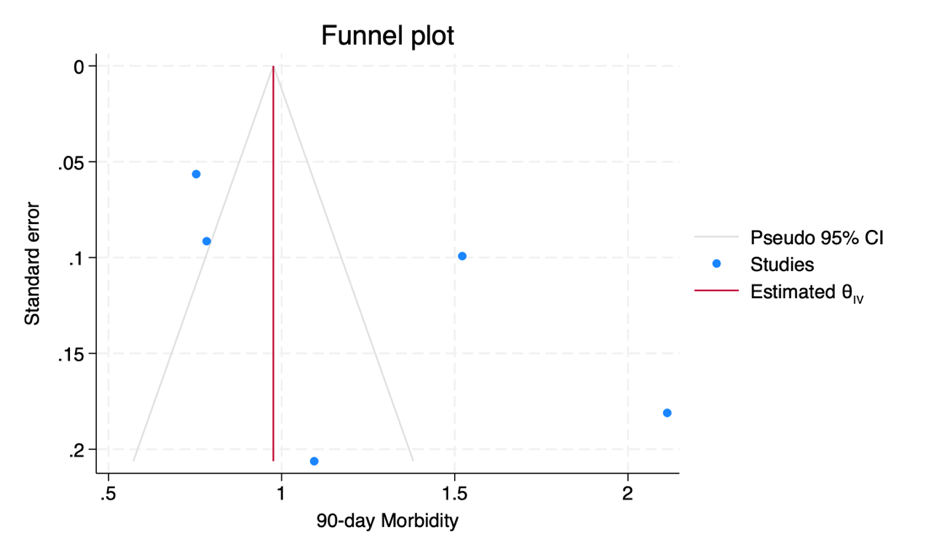


**Supplementary Funnel Plot 11:** Clavien-Dindo Grade 1 or 2


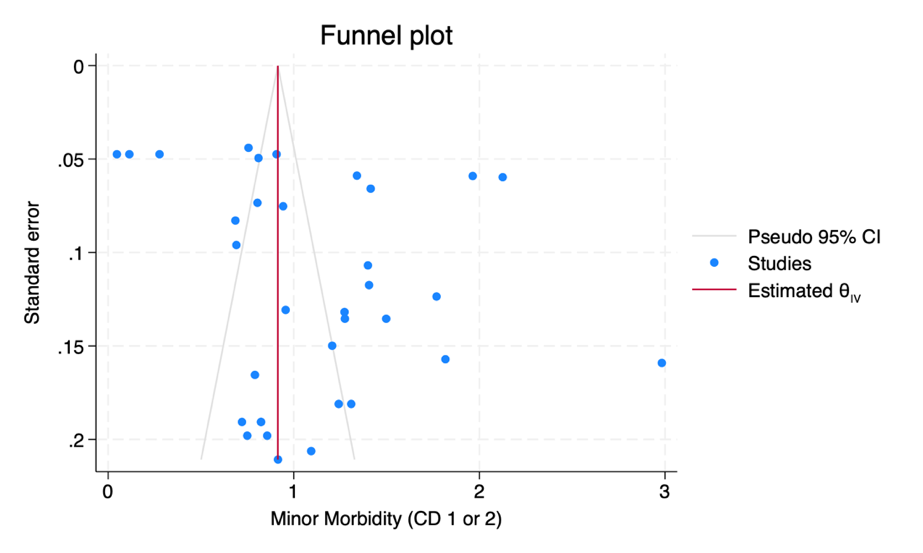


**Supplementary Funnel Plot 12:** Clavien-Dindo Grade ≥3


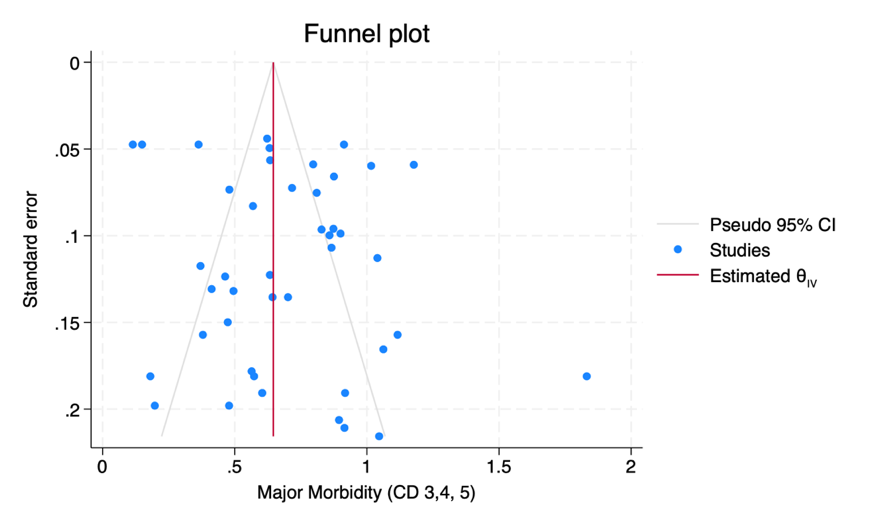


**Supplementary Funnel Plot 13:** In-Hospital Mortality


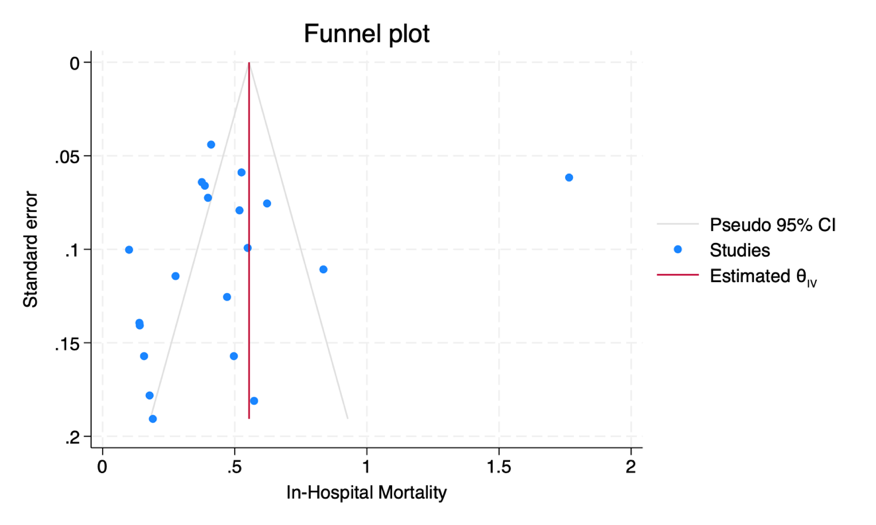


**Supplementary Funnel Plot 14:** 30-Day Mortality


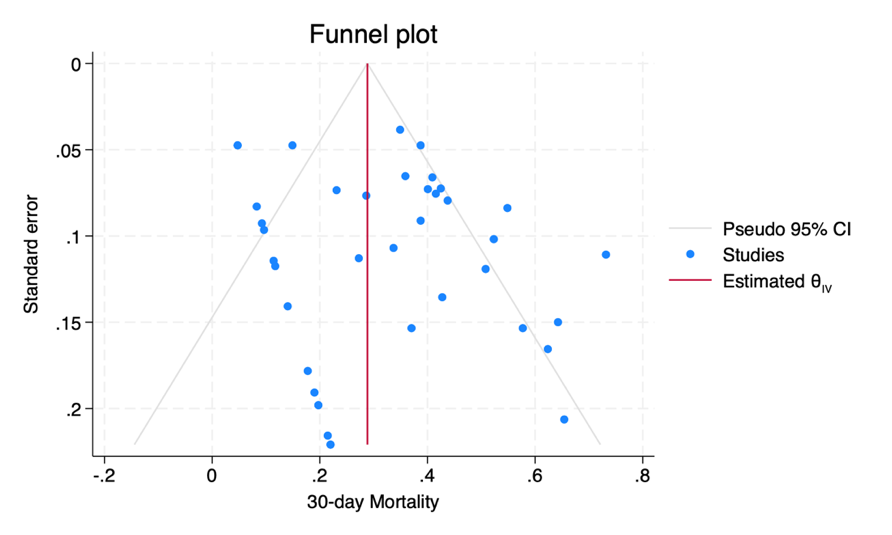


**Supplementary Funnel Plot 15:** 90-Day Mortality


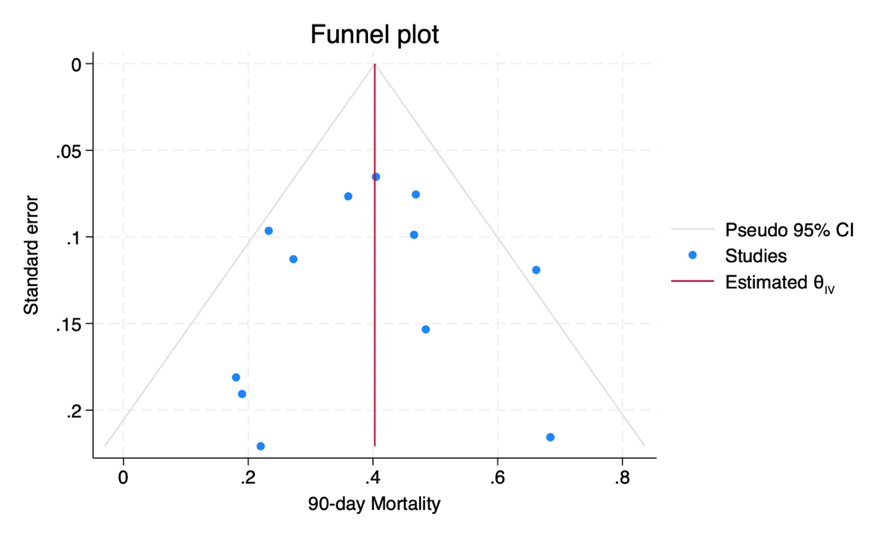


**Supplementary Funnel Plot 16:** Liver Failure or Dysfunction


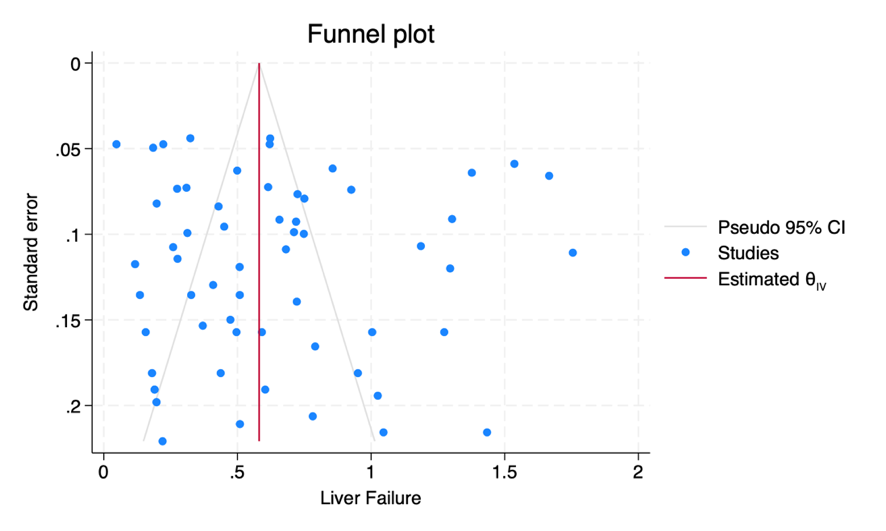


**Supplementary Forest Plot 17:** Bile Leaks


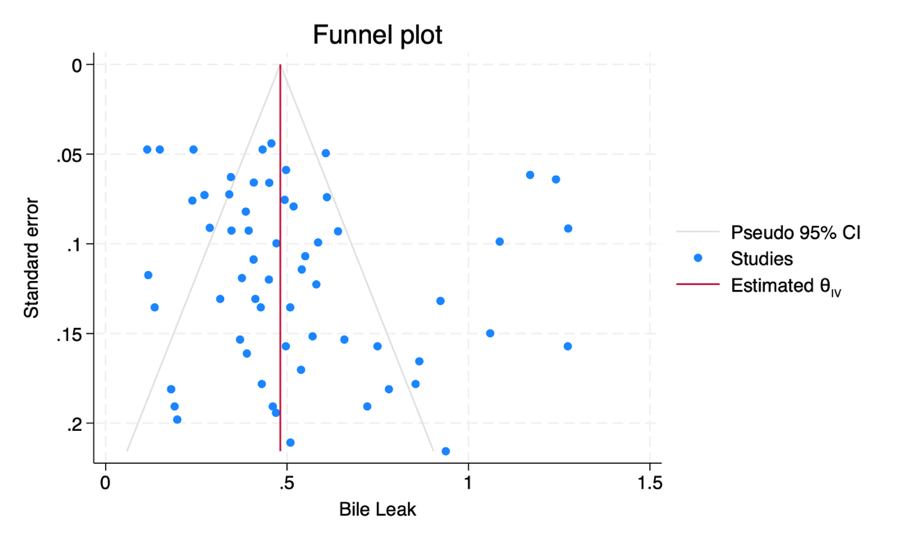


**Supplementary Funnel Plot 18:** Hemorrhage


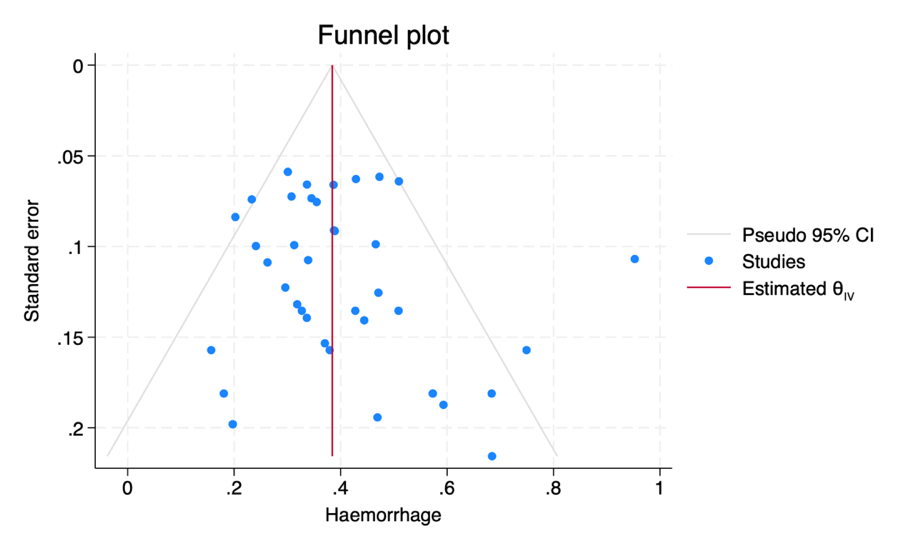


**Supplementary Funnel Plot 19:** Myocardial Infarction


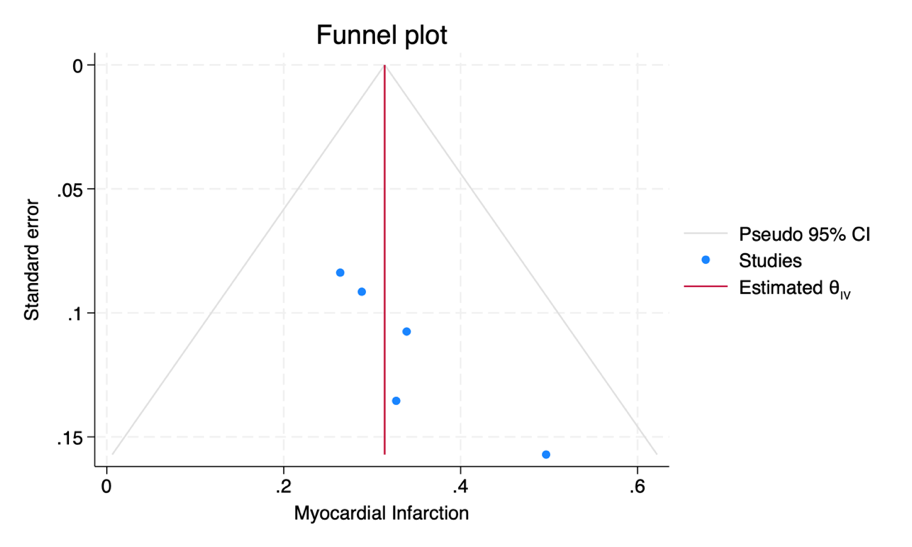


**Supplementary Funnel Plot 20:** Thrombotic or Thromboembolic Events


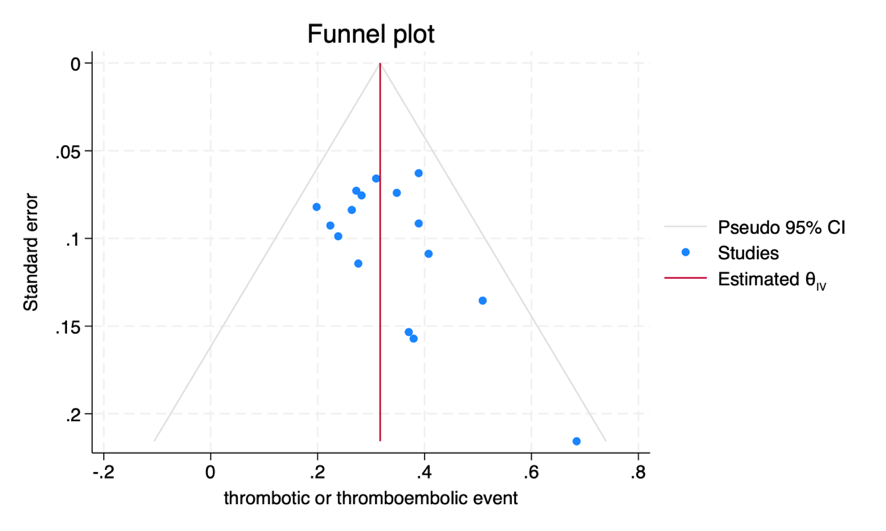


**Supplementary Funnel Plot 21:** Cerebrovascular Accidents


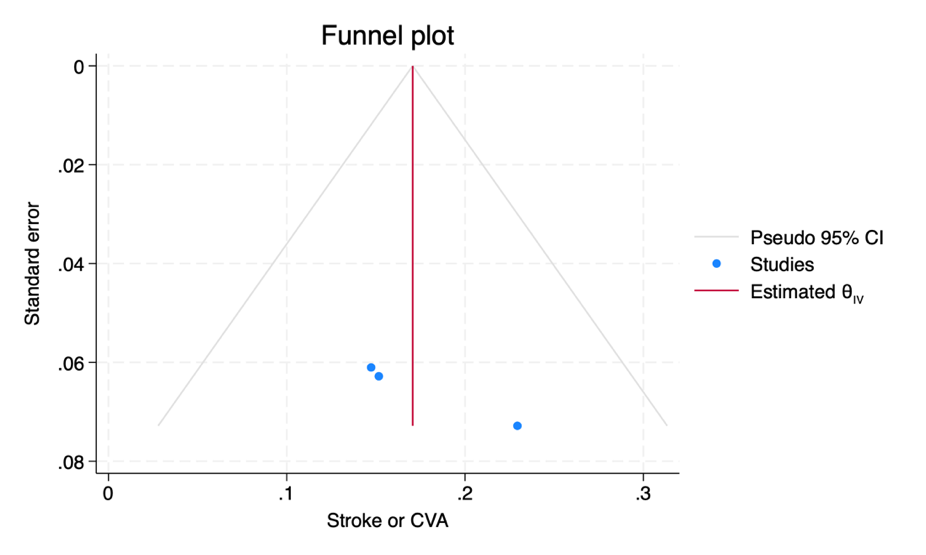


**Supplementary Funnel Plot 22:** Pneumonia


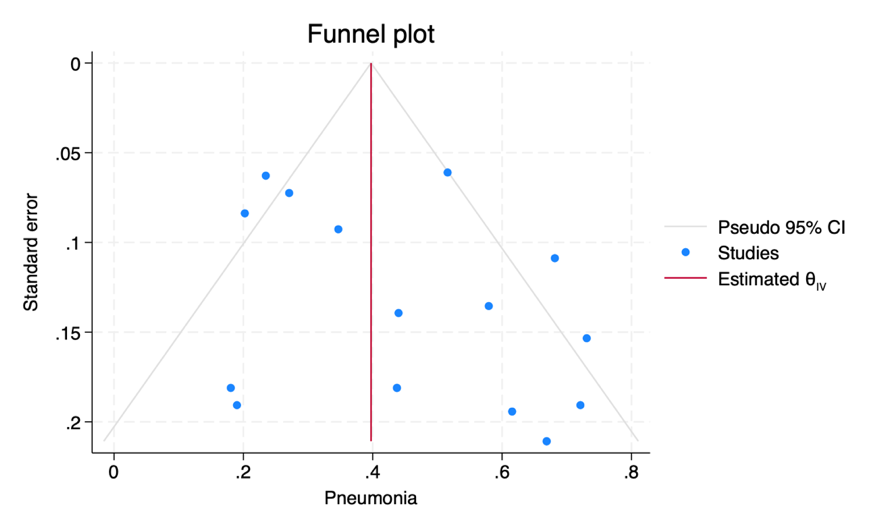


**Supplementary Funnel Plot 23:** Liver Abscess


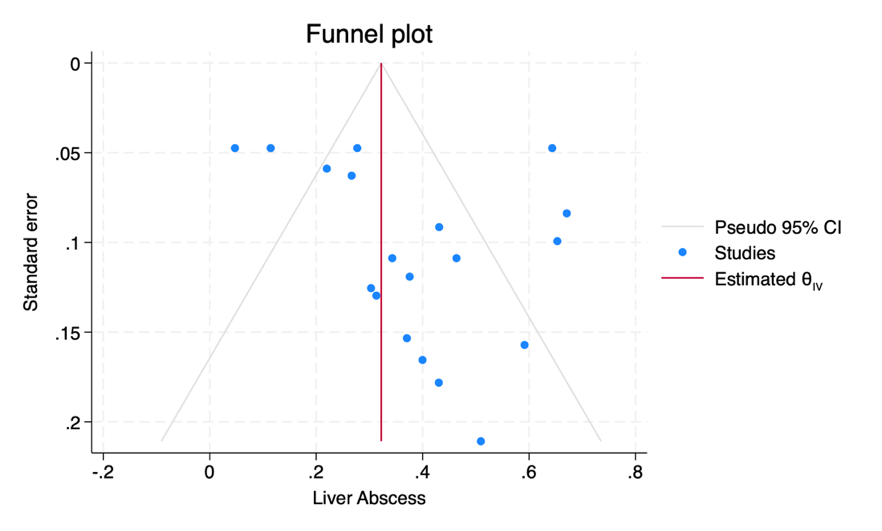


**Supplementary Funnel Plot 24:** Wound Dehiscence or Infection


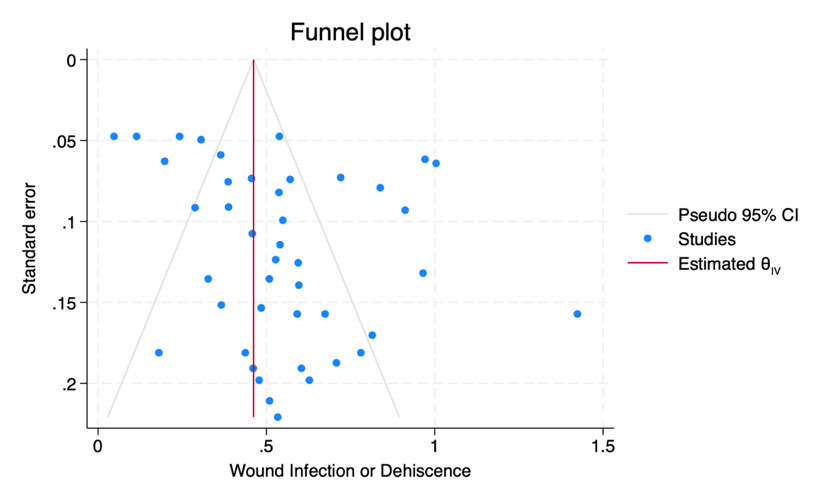


**Supplementary Funnel Plot 25:** Urinary Tract Infection


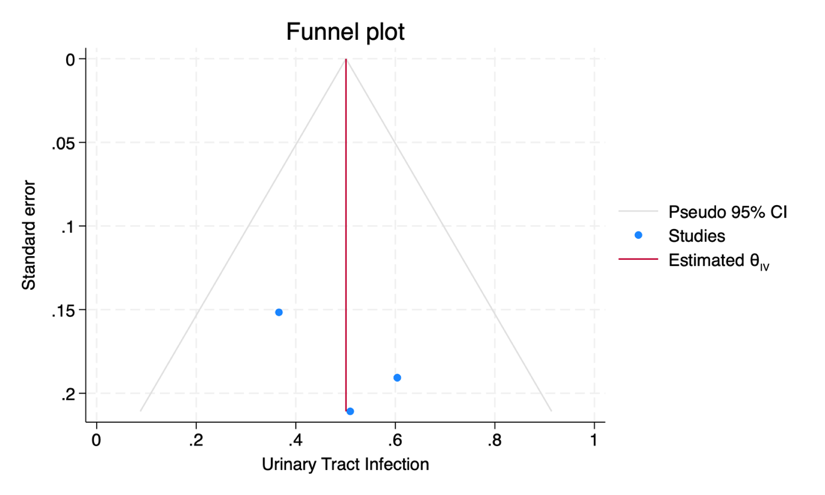


**Supplementary Funnel Plot 26:** Bacteremia


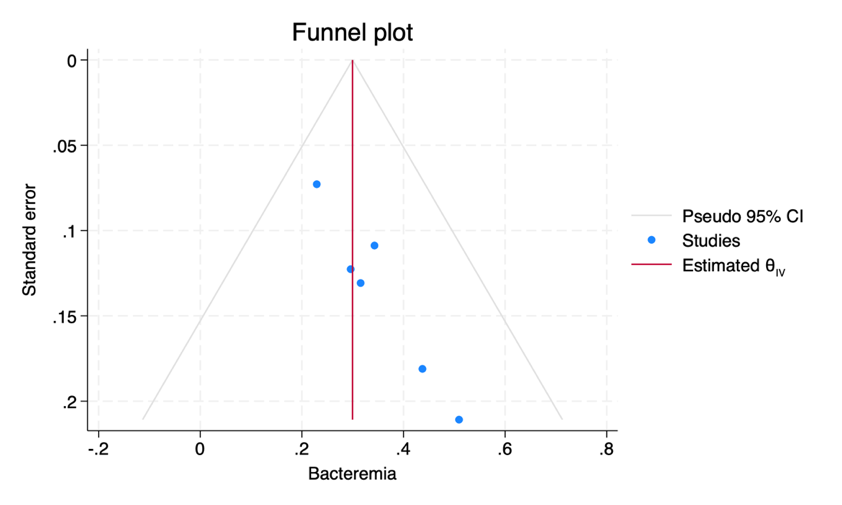


**Supplementary Funnel Plot 27:** Other Infections


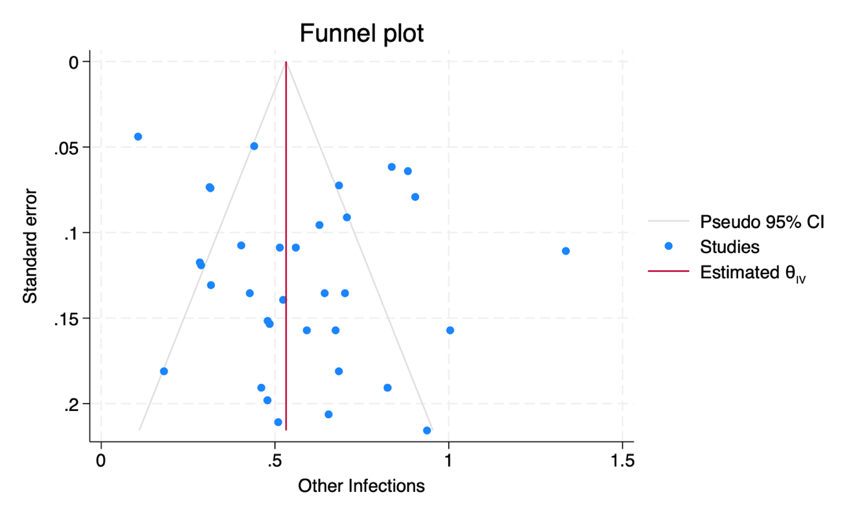


**Section 5 : Risk of Bias Assessment**

**Case Series (N= 24)**

**Question 1**: Was the study question or objective clearly stated?

**Question 2**: Was the study population clearly and fully described, including a case definition?

**Question 3**: Were the cases consecutive?

**Question 4**: Were the subjects comparable?

**Question 5**: Was the intervention clearly described?

**Question 6**: Were the outcome measures clearly defined, valid, reliable, and implemented consistently across all study participants?

**Question 7**: Was the length of follow-up adequate?

**Question 8**: Were the statistical methods well-described?

**Question 9**: Were the results well-described?

| **Study and Year** | **Domain** | | | | | | | | | **Total Score** | **Quality Rating** |
| --- | --- | --- | --- | --- | --- | --- | --- | --- | --- | --- | --- |
|  | **Q1** | **Q2** | **Q3** | **Q4** | **Q5** | **Q6** | **Q7** | **Q8** | **Q9** |  |  |
| **Abdelraouf 2014** | Y | Y | Y | Y | Y | Y | Y | N/A | Y | 8 | **Good** |
| **Ardiles 2010** | Y | Y | N | Y | Y | Y | Y | Y | Y | 8 | **Good** |
| **Aysal 2022** | Y | Y | N | Y | Y | Y | Y | Y | Y | 8 | **Good** |
| **Bacalbasa 2014** | Y | Y | N | Y | Y | Y | Y | Y | Y | 8 | **Good** |
| **Bari 2021** | Y | Y | N | Y | Y | Y | Y | Y | Y | 8 | **Good** |
| **Belev 2007** | Y | Y | N | Y | Y | Y | N | N | Y | 6 | **Fair** |
| **Bhaijee 2011** | Y | Y | N | Y | Y | Y | Y | N | Y | 7 | **Good** |
| **Daradkeh 2021** | Y | Y | N | Y | Y | Y | Y | Y | Y | 9 | **Good** |
| **De Santibanes 2015** | Y | N/A | N/A | Y | Y | Y | Y | Y | Y | 7 | **Good** |
| **Fadel 2020** | Y | Y | N | Y | Y | Y | Y | Y | Y | 8 | **Good** |
| **Fontana 2014** | Y | Y | N/A | Y | Y | Y | N/A | Y | N | 6 | **Fair** |
| **Herman 2014** | Y | Y | Y | Y | Y | N/A | Y | Y | N | 7 | **Good** |
| **Herman 2016** | Y | Y | N/A | Y | Y | Y | Y | Y | Y | 8 | **Good** |
| **Ho 2021** | Y | Y | N | Y | Y | Y | N | Y | Y | 7 | **Good** |
| **Machado 2017** | Y | Y | N | Y | Y | Y | Y | N | Y | 7 | **Good** |
| **Makdissi 2021** | Y | Y | N | Y | Y | Y | Y | N | Y | 7 | **Good** |
| **Ninh 2021** | Y | Y | N | Y | Y | Y | Y | Y | Y | 8 | **Good** |
| **Pandey 2018** | Y | Y | Y | Y | Y | Y | Y | Y | Y | 9 | **Good** |
| **Patkar 2019** | Y | Y | N | N | Y | Y | Y | Y | Y | 7 | **Good** |
| **Patkar 2020** | Y | Y | Y | N | Y | Y | Y | Y | Y | 8 | **Good** |
| **Patkar 2021** | Y | Y | N | N | Y | Y | Y | Y | Y | 7 | **Good** |
| **Petrovic 2010** | Y | Y | Y | N/A | Y | Y | Y | Y | Y | 8 | **Good** |
| **Pottakkat 2013** | Y | Y | N/A | Y | Y | Y | Y | Y | Y | 8 | **Good** |
| **Sapmaz 2020** | Y | Y | N | N | Y | Y | Y | N | Y | 6 | **Fair** |
| *Y: Yes; N: No; N/A: Not Applicable* | | | | | | | | | | | |

**Observational Cohort and Cross-Sectional Studies (N= 100)**

**Question 1**: Was the research question or objective in this paper clearly stated?

**Question 2**: Was the study population clearly specified and defined?

**Question 3**: Was the participation rate of eligible persons at least 50%?

**Question 4**: Were all the subjects selected or recruited from the same or similar populations (including the same time period)? Were inclusion and exclusion criteria for being in the study prespecified and applied uniformly to all participants?

**Question 5**: Was a sample size justification, power description, or variance and effect estimates provided?

**Question 6**: For the analyses in this paper, were the exposure(s) of interest measured prior to the outcome(s) being measured?

**Question 7**: Was the timeframe sufficient so that one could reasonably expect to see an association between exposure and outcome if it existed?

**Question 8**: For exposures that can vary in amount or level, did the study examine different levels of the exposure as related to the outcome (e.g., categories of exposure, or exposure measured as continuous variable)?

**Question 9**: Were the exposure measures (independent variables) clearly defined, valid, reliable, and implemented consistently across all study participants?

**Question 10:** Was the exposure(s) assessed more than once over time?

**Question 11:** Were the outcome measures (dependent variables) clearly defined, valid, reliable, and implemented consistently across all study participants?

**Question 12:** Were the outcome assessors blinded to the exposure status of participants?

**Question 13:** Was loss to follow-up after baseline 20% or less?

**Question 14:** Were key potential confounding variables measured and adjusted statistically for their impact on the relationship between exposure(s) and outcome(s)?

| **Study and Year** | **Domain** | | | | | | | | | | | | | | **Total Score** | **Quality Rating** |
| --- | --- | --- | --- | --- | --- | --- | --- | --- | --- | --- | --- | --- | --- | --- | --- | --- |
|  | **Q1** | **Q2** | **Q3** | **Q4** | **Q5** | **Q6** | **Q7** | **Q8** | **Q9** | **Q10** | **Q11** | **Q12** | **Q13** | **Q14** |  |  |
| **Abdel-Wahab 2010** | Y | Y | Y | N | N/A | Y | Y | Y | Y | N/A | Y | N/A | Y | Y | 10 | **Fair** |
| **Abreu 2020** | Y | N | Y | Y | N | N/A | Y | N/A | Y | N | Y | N/A | N | Y | 7 | **Fair** |
| **Agarwal 2015** | Y | Y | N | Y | N | Y | Y | N/A | Y | N/A | Y | N/A | N | Y | 8 | **Fair** |
| **Ainthachot 2022** | Y | Y | Y | Y | N | Y | Y | N/A | Y | N/A | Y | N | N/A | Y | 9 | **Fair** |
| **Akcam 2019** | Y | Y | N/A | Y | N | Y | Y | N/A | N | N/A | Y | N | N | Y | 7 | **Fair** |
| **Aksoy 2020** | Y | Y | Y | Y | N | Y | Y | N/A | Y | N/A | Y | N/A | Y | Y | 10 | **Fair** |
| **Alexandrescu 2012** | Y | Y | Y | Y | N | Y | Y | N/A | Y | N/A | Y | N/A | N | Y | 9 | **Fair** |
| **Alexandrescu 2017** | Y | Y | Y | Y | N | Y | Y | N/A | Y | N/A | Y | N/A | N/A | Y | 9 | **Fair** |
| **Alvarez 2015** | Y | Y | Y | Y | N | Y | Y | N/A | Y | N/A | Y | N/A | N | Y | 9 | **Fair** |
| **Aristizabal 2018** | Y | Y | Y | N | N | Y | N | N/A | N/A | N | Y | N/A | Y | Y | 7 | **Fair** |
| **Bacalbasa 2015** | Y | Y | N/A | Y | N | Y | Y | Y | Y | N/A | Y | N/A | Y | Y | 10 | **Fair** |
| **Batra 2016** | Y | Y | Y | Y | N | Y | Y | N/A | Y | N/A | Y | N | Y | Y | 10 | **Fair** |
| **Bhandare 2018** | Y | Y | Y | Y | N | Y | Y | Y | Y | N | Y | N/A | N/A | Y | 10 | **Fair** |
| **Bogdanovic 2020** | Y | Y | Y | N | N/A | Y | N/A | N/A | Y | Y | Y | N/A | Y | N/A | 8 | **Fair** |
| **Bonadio 2019** | Y | Y | N/A | Y | N | Y | Y | Y | Y | N/A | Y | N/A | N/A | Y | 9 | **Fair** |
| **Bredt 2014** | Y | Y | N/A | Y | N | Y | Y | Y | Y | N/A | Y | N/A | N/A | Y | 9 | **Fair** |
| **Burlaka 2020** | N | Y | N/A | Y | N | Y | Y | N/A | Y | N/A | Y | N/A | N/A | N/A | 6 | **Fair** |
| **Chaudhari 2018** | Y | Y | Y | Y | N | Y | Y | Y | Y | N/A | Y | N | Y | Y | 11 | **Good** |
| **Chinburen 2015** | Y | Y | Y | Y | N | Y | Y | N/A | N | N | Y | N/A | Y | Y | 9 | **Fair** |
| **Chotirosniramit 2020** | Y | Y | Y | Y | Y | N/A | Y | N/A | Y | N | Y | N/A | Y | Y | 10 | **Fair** |
| **Civil 2020** | Y | Y | Y | Y | N/A | Y | Y | N/A | Y | N/A | Y | N/A | Y | N/A | 9 | **Fair** |
| **Cokmert 2014** | Y | Y | Y | N | N/A | Y | Y | N/A | Y | Y | Y | N/A | Y | N/A | 9 | **Fair** |
| **Costa 2022** | Y | Y | N/A | N | N | Y | Y | N/A | Y | N/A | Y | N | N/A | N | 6 | **Fair** |
| **deSantibanes 2010** | Y | Y | Y | Y | N/A | Y | Y | N/A | Y | N | Y | N/A | Y | Y | 10 | **Fair** |
| **Diaconescu 2017** | Y | Y | Y | Y | N | Y | Y | N/A | Y | N/A | Y | N | Y | N | 9 | **Fair** |
| **Dulundu 2017** | Y | Y | N/A | Y | N | Y | Y | N/A | Y | N/A | Y | N/A | N/A | Y | 8 | **Fair** |
| **Dumitrascu 2016** | Y | Y | Y | Y | N/A | Y | Y | N/A | Y | N/A | Y | N/A | Y | Y | 10 | **Fair** |
| **Dumitrascu 2017** | Y | N | Y | N | N/A | Y | Y | Y | Y | N/A | Y | N/A | Y | N/A | 8 | **Fair** |
| **Efanov 2020** | Y | Y | Y | Y | Y | Y | Y | Y | Y | N/A | Y | N/A | Y | Y | 12 | **Good** |
| **El-Gendi 2013** | Y | Y | Y | Y | N | Y | Y | Y | Y | N/A | Y | N/A | Y | Y | 11 | **Good** |
| **Elshaarawy 2021** | Y | Y | N/A | Y | N | Y | Y | N/A | Y | N | Y | N | N | Y | 8 | **Fair** |
| **Galun 2012** | Y | Y | Y | N | N/A | Y | Y | Y | Y | N/A | Y | N/A | N | N/A | 8 | **Fair** |
| **Galun 2018** | Y | N | Y | N | N/A | Y | Y | Y | Y | Y | Y | N/A | Y | N/A | 9 | **Fair** |
| **Galun 2021** | Y | Y | Y | Y | N | Y | Y | N/A | Y | N/A | Y | N/A | N | Y | 9 | **Fair** |
| **Goel 2022** | Y | Y | Y | Y | N | Y | Y | N/A | Y | N/A | Y | N | Y | N | 9 | **Fair** |
| **Govil 2016** | Y | Y | Y | N | N/A | Y | Y | N/A | Y | N | Y | N | Y | N/A | 8 | **Fair** |
| **Goyal 2021** | Y | Y | N/A | Y | N | Y | Y | N/A | Y | N/A | Y | N/A | N/A | Y | 8 | **Fair** |
| **Grigorie 2017** | Y | Y | Y | N | N/A | Y | Y | Y | Y | N/A | Y | N/A | Y | N/A | 9 | **Fair** |
| **Gupta 2022** | Y | Y | Y | Y | N | Y | Y | N/A | Y | N/A | Y | N | Y | Y | 10 | **Fair** |
| **Hegazy 2019** | Y | Y | Y | Y | Y | N/A | Y | Y | Y | Y | Y | Y | N/A | Y | 12 | **Good** |
| **Iancu 2008** | Y | Y | Y | N | Y | N/A | Y | Y | Y | N/A | Y | N/A | Y | Y | 10 | **Fair** |
| **Ibraheem 2022** | Y | Y | Y | Y | N | Y | Y | N/A | Y | N/A | Y | N | Y | N | 9 | **Fair** |
| **Jayme 2021** | Y | Y | Y | Y | N/A | Y | Y | N/A | Y | N | Y | N/A | Y | Y | 10 | **Fair** |
| **Joshi 2021** | Y | Y | Y | Y | N | Y | Y | N | Y | N/A | Y | N/A | Y | Y | 10 | **Fair** |
| **Kalayarasan 2013** | Y | Y | Y | N | N | Y | Y | N/A | N | N/A | Y | N | N | N | 6 | **Fair** |
| **Kavlakoglu 2011** | Y | N | N/A | N/A | N | Y | Y | Y | Y | N/A | Y | N/A | Y | Y | 8 | **Fair** |
| **Khuntikeo 2008** | Y | Y | Y | Y | N | Y | Y | N/A | Y | N/A | Y | Y | Y | N/A | 10 | **Fair** |
| **Kostov 2009** | Y | Y | N/A | Y | N | Y | Y | Y | Y | N | Y | N/A | Y | N/A | 9 | **Fair** |
| **Kostov 2013** | Y | Y | Y | Y | N | Y | Y | Y | Y | N | Y | N/A | Y | Y | 11 | **Good** |
| **Kruger 2018** | Y | N | N | N | N | Y | Y | Y | Y | N/A | Y | N | Y | Y | 8 | **Fair** |
| **Kumar 2019** | Y | Y | Y | Y | N | Y | Y | Y | N | Y | Y | N | Y | Y | 11 | **Good** |
| **Leeratanakachorn 2021** | Y | Y | Y | Y | N | Y | Y | N/A | Y | N/A | Y | N/A | Y | N | 9 | **Fair** |
| **Long 2013** | Y | Y | Y | N | N | Y | Y | Y | Y | N/A | Y | N | N | N/A | 8 | **Fair** |
| **Lopes 2016** | Y | N | Y | N | N | Y | Y | N/A | Y | N/A | Y | N | Y | N | 7 | **Fair** |
| **Luna-Abanto 2020** | Y | N | Y | N | N | Y | Y | Y | Y | N/A | Y | N | Y | N | 8 | **Fair** |
| **Mannai 2010** | Y | Y | Y | Y | N | Y | Y | N/A | Y | N/A | Y | N | Y | N | 9 | **Fair** |
| **Marques 2018** | Y | Y | Y | Y | N/A | Y | Y | N/A | Y | N | Y | N/A | Y | N/A | 9 | **Fair** |
| **Maurette 2017** | Y | N | Y | N | N | Y | Y | N/A | Y | N/A | Y | N | Y | N | 7 | **Fair** |
| **Meira Junior 2022** | Y | Y | N/A | Y | N | Y | Y | Y | Y | N | Y | N | N/A | N | 8 | **Fair** |
| **Mogahed 2021** | Y | Y | Y | Y | N/A | Y | Y | Y | Y | N/A | Y | N/A | Y | Y | 11 | **Good** |
| **Molek 2021** | Y | Y | Y | Y | N/A | Y | Y | N/A | Y | N/A | Y | N/A | Y | Y | 10 | **Fair** |
| **Nari 2018** | Y | Y | N/A | Y | N | Y | Y | N | Y | Y | Y | N/A | N/A | N | 8 | **Fair** |
| **Negi 2011** | Y | Y | Y | Y | N/A | Y | Y | N/A | Y | N | Y | N/A | Y | Y | 10 | **Fair** |
| **Nicolas 2022** | Y | Y | N/A | Y | N | Y | Y | Y | Y | Y | Y | N | N/A | N | 9 | **Fair** |
| **Panwar 2016** | Y | Y | Y | Y | N | Y | Y | N/A | Y | N | Y | N | Y | N | 9 | **Fair** |
| **Parau 2015** | Y | Y | Y | Y | Y | Y | Y | Y | Y | N/A | Y | Y | Y | Y | 13 | **Good** |
| **Patkar 2018** | Y | Y | Y | Y | N/A | Y | Y | N/A | Y | N/A | Y | N/A | Y | N/A | 9 | **Fair** |
| **Popescu 2005** | N | Y | Y | N | N | Y | Y | N | Y | N/A | Y | N/A | Y | N/A | 7 | **Fair** |
| **Popescu 2012** | Y | Y | Y | Y | Y | N/A | Y | N/A | Y | N | Y | N/A | Y | Y | 10 | **Fair** |
| **Quesada-Soto 2017** | Y | Y | Y | Y | N | Y | Y | N | N | N | Y | N/A | N/A | N | 7 | **Fair** |
| **Qureshi 2020** | Y | Y | Y | Y | N | Y | Y | N | Y | Y | Y | N/A | N | Y | 10 | **Fair** |
| **Rachdi 2019** | Y | Y | Y | Y | N | Y | Y | N | Y | Y | Y | N/A | N | N | 9 | **Fair** |
| **Rammohan 2015** | Y | Y | Y | Y | N | Y | Y | N | Y | N | N | N/A | N/A | N | 7 | **Fair** |
| **Ribeiro 2012** | Y | Y | Y | Y | N | Y | Y | N/A | Y | N/A | Y | N | Y | N | 9 | **Fair** |
| **Ribeiro 2013** | Y | Y | Y | Y | N | Y | Y | N | N | N | Y | N/A | N/A | N | 7 | **Fair** |
| **Ruiz 2016** | Y | Y | N/A | Y | N | Y | Y | N | N | N/A | Y | N/A | N/A | N | 6 | **Fair** |
| **Ruiz 2022** | Y | Y | N/A | Y | N | Y | Y | Y | Y | Y | Y | N | N/A | N | 9 | **Fair** |
| **Rungsakulkij 2018** | Y | Y | N/A | Y | N | Y | Y | N | Y | N/A | Y | N/A | N/A | N | 7 | **Fair** |
| **Saglam 2022** | Y | Y | Y | Y | N | Y | Y | N/A | Y | N/A | Y | N | N/A | N | 8 | **Fair** |
| **Said 2021** | Y | Y | N/A | Y | N | Y | Y | Y | Y | Y | Y | N | Y | N | 10 | **Fair** |
| **Saritas 2020** | Y | Y | N/A | Y | N | Y | Y | Y | Y | N/A | Y | N/A | N/A | N | 8 | **Fair** |
| **Sawangkajohn 2020** | Y | Y | N/A | Y | N | Y | Y | N | Y | N | Y | N | N/A | N | 7 | **Fair** |
| **Senbel 2017** | Y | Y | Y | Y | N | Y | Y | N | Y | Y | Y | N | N/A | N | 9 | **Fair** |
| **Shehta 2020** | Y | Y | N/A | Y | N | Y | Y | N/A | Y | N/A | Y | N/A | N/A | N | 7 | **Fair** |
| **Shehta 2021** | Y | Y | N/A | Y | N/A | Y | Y | N/A | Y | N/A | Y | N/A | N/A | Y | 8 | **Fair** |
| **Shehta 2021** | Y | Y | N/A | N | N/A | Y | Y | N | Y | N/A | Y | N/A | N/A | Y | 7 | **Fair** |
| **Soliman 2017** | Y | Y | N/A | Y | N | Y | Y | N | Y | N | N | N/A | N/A | Y | 7 | **Fair** |
| **Sriputtha 2013** | Y | Y | Y | Y | Y | Y | Y | N/A | Y | N/A | Y | N/A | Y | Y | 11 | **Good** |
| **Taesombat 2020** | Y | Y | N/A | N | N | Y | Y | N | Y | N | Y | N | N/A | Y | 7 | **Fair** |
| **Taesombat 2020** | Y | Y | N/A | Y | N | Y | Y | N | Y | N | Y | N/A | N/A | N | 7 | **Fair** |
| **Takorov 2016** | Y | Y | N/A | N/A | N | Y | Y | N | N | N | Y | N | N/A | N | 5 | **Poor** |
| **Techathuvanan 2015** | Y | Y | N/A | Y | N | Y | Y | Y | Y | N | N | N/A | N/A | Y | 8 | **Fair** |
| **Tohra 2021** | Y | Y | N/A | Y | N | Y | Y | N/A | Y | N | N | N | N | N | 6 | **Fair** |
| **Tomas 2020** | Y | Y | N/A | N | N | Y | Y | N | Y | N/A | Y | N/A | N/A | N | 6 | **Fair** |
| **Valadares 2015** | Y | Y | Y | Y | N | Y | Y | N | Y | N | Y | N/A | N/A | N | 8 | **Fair** |
| **VazdaSilva 2020** | N | Y | Y | N | N/A | Y | Y | N/A | Y | N | Y | N | Y | N/A | 7 | **Fair** |
| **Wahab 2012** | Y | Y | N/A | Y | N | Y | Y | N | Y | N | Y | N/A | N/A | N/A | 7 | **Fair** |
| **Wahab 2014** | Y | Y | N/A | N | N | Y | Y | N/A | N/A | N | N | N | N/A | N | 4 | **Poor** |
| **Younes 2020** | Y | Y | N/A | Y | N | Y | Y | N | Y | N/A | Y | N/A | N/A | N | 7 | **Fair** |
| **Zakaria 2020** | Y | Y | Y | Y | N | Y | Y | N/A | Y | N/A | Y | N | N/A | N | 8 | **Fair** |
| *Y: Yes; N: No; N/A: Not Applicable* | | | | | | | | | | | | | | | | |

**Case-Control Studies (N= 5)**

**Question 1**: Was the research question or objective in this paper clearly stated and appropriate?

**Question 2**: Was the study population clearly specified and defined?

**Question 3**: Did the authors include a sample size justification?

**Question 4**: Were controls selected or recruited from the same or similar population that gave rise to the cases (including the same timeframe)?

**Question 5**: Were the definitions, inclusion and exclusion criteria, algorithms or processes used to identify or select cases and controls valid, reliable, and implemented consistently across all study participants?

**Question 6**: Were the cases clearly defined and differentiated from controls?

**Question 7**: If less than 100 percent of eligible cases and/or controls were selected for the study, were the cases and/or controls randomly selected from those eligible?

**Question 8**: Was there use of concurrent controls?

**Question 9**: Were the investigators able to confirm that the exposure/risk occurred prior to the development of the condition or event that defined a participant as a case?

**Question 10:** Were the measures of exposure/risk clearly defined, valid, reliable, and implemented consistently (including the same time period) across all study participants?

**Question 11:** Were the assessors of exposure/risk blinded to the case or control status of participants?

**Question 12:** Were key potential confounding variables measured and adjusted statistically in the analyses? If matching was used, did the investigators account for matching during study analysis?

| **Study and Year** | **Domain** | | | | | | | | | | | | **Total Score** | **Quality Rating** |
| --- | --- | --- | --- | --- | --- | --- | --- | --- | --- | --- | --- | --- | --- | --- |
|  | **Q1** | **Q2** | **Q3** | **Q4** | **Q5** | **Q6** | **Q7** | **Q8** | **Q9** | **Q10** | **Q11** | **Q12** |  |  |
| **Abdelwahab 2014** | Y | Y | N | Y | Y | Y | N/A | N/A | N/A | Y | N/A | Y | 7 | **Fair** |
| **Abdel Wahab 2015** | Y | Y | N | N/A | Y | Y | N/A | N | N/A | N/A | N | Y | 5 | **Fair** |
| **Elsanousi 2018** | Y | Y | N | Y | N/A | Y | N/A | N/A | Y | Y | N/A | N/A | 6 | **Fair** |
| **Makhlouf 2020** | Y | Y | N | N | Y | Y | N/A | Y | Y | Y | N | N | 7 | **Fair** |
| **Nag 2021** | Y | Y | Y | Y | Y | Y | N/A | N | Y | Y | N | Y | 9 | **Good** |
| *Y: Yes; N: No; N/A: Not Applicable* | | | | | | | | | | | | | | |

**Quality assessment for Randomized Control Trials (N=6)**

**Question 1**: Was the study described as randomized, a randomized trial, a randomized clinical trial, or an RCT?

**Question 2**: Was the method of randomization adequate (i.e., use of randomly generated assignment)?

**Question 3**: Was the treatment allocation concealed (so that assignments could not be predicted)?

**Question 4**: Were study participants and providers blinded to treatment group assignment?

**Question 5**: Were the people assessing the outcomes blinded to the participants' group assignments?

**Question 6**: Were the groups similar at baseline on important characteristics that could affect outcomes (e.g., demographics, risk factors, co-morbid conditions)?

**Question 7**: Was the overall drop-out rate from the study at endpoint 20% or lower of the number allocated to treatment?

**Question 8**: Was the differential drop-out rate (between treatment groups) at endpoint 15 percentage points or lower?

**Question 9**: Was there high adherence to the intervention protocols for each treatment group?

**Question 10:** Were other interventions avoided or similar in the groups (e.g., similar background treatments)?

**Question 11:** Were outcomes assessed using valid and reliable measures, implemented consistently across all study participants?

**Question 12:** Did the authors report that the sample size was sufficiently large to be able to detect a difference in the main outcome between groups with at least 80% power?

**Question 13:** Were outcomes reported or subgroups analyzed prespecified (i.e., identified before analyses were conducted)?

**Question 14:** Were all randomized participants analyzed in the group to which they were originally assigned, i.e., did they use an intention-to-treat analysis?

| **Study and Year** | **Domains** | | | | | | | | | | | | | | **Total score** | **Quality Rating** |
| --- | --- | --- | --- | --- | --- | --- | --- | --- | --- | --- | --- | --- | --- | --- | --- | --- |
|  | **Q1** | **Q2** | **Q3** | **Q4** | **Q5** | **Q6** | **Q7** | **Q8** | **Q9** | **Q10** | **Q11** | **Q12** | **Q13** | **Q14** |  |  |
| **Abd El-Kader 2018*** | Y | N | N | N | N/A | Y | N | N | Y | Y | Y | N | N/A | Y | 6 | **Fair** |
| **El-Gendi 2018** | Y | Y | Y | Y | N/A | Y | Y | Y | Y | N/A | Y | N/A | N/A | Y | 10 | **Fair** |
| **El-Gendi 2018** | Y | Y | Y | N/A | Y | Y | Y | Y | Y | N/A | Y | N/A | Y | Y | 11 | **Good** |
| **Khalil 2018** | Y | N/A | N/A | N/A | N/A | Y | N | Y | Y | Y | Y | N | N/A | Y | 7 | **Fair** |
| **Saber 2020** | Y | Y | Y | Y | Y | Y | Y | Y | Y | N/A | Y | Y | N/A | Y | 12 | **Good** |
| **Tongsiri 2020** | Y | Y | Y | Y | Y | Y | Y | Y | Y | Y | Y | Y | N | Y | 13 | **Good** |
| *Quasi-Experimental Study  *Y: Yes; N: No; N/A: Not Applicable* | | | | | | | | | | | | | | | | |
